# A spliceosomal twin intron (stwintron) participates in both exon skipping and evolutionary exon loss

**DOI:** 10.1038/s41598-019-46435-x

**Published:** 2019-07-09

**Authors:** Napsugár Kavalecz, Norbert Ág, Levente Karaffa, Claudio Scazzocchio, Michel Flipphi, Erzsébet Fekete

**Affiliations:** 1 Department of Biochemical Engineering, University of Debrecen, Debrecen, 4032 Hungary; 2 Department of Microbiology, Imperial College London, London, SW7 2AZ UK; 3 Institut de Biologie Intégrative de la Cellule, Centre National de la Recherche Scientifique – Unité Mixte de Recherche 9198, Gif-sur-Yvette, 91405 France

**Keywords:** Molecular evolution, RNA splicing

## Abstract

Spliceosomal twin introns (stwintrons) are introns where any of the three consensus sequences involved in splicing is interrupted by another intron (internal intron). In *Aspergillus nidulans*, a donor-disrupted stwintron (intron-1) is extant in the transcript encoding a reticulon-like protein. The orthologous transcript of *Aspergillus niger* can be alternatively spliced; the exon downstream the stwintron could be skipped by excising a sequence that comprises this stwintron, the neighbouring intron-2, and the exon bounded by these. This process involves the use of alternative 3′ splice sites for the internal intron, the resulting alternative intervening sequence being a longer 3′-extended stwintron. In 29 species of *Onygenales*, a multi-step splicing process occurs in the orthologous transcript, in which a complex intervening sequence including the stwintron and neigbouring intron-2, generates by three splicing reactions a “second order intron” which must then be excised with a fourth splicing event. The gene model in two species can be envisaged as one canonical intron (intron-1) evolved from this complex intervening sequence of nested canonical introns found elsewhere in *Onygenales*. Postulated splicing intermediates were experimentally verified in one or more species. This work illustrates a role of stwintrons in both alternative splicing and the evolution of intron structure.

## Introduction

In Eukaryotes, the primary transcripts of nuclear genes are frequently interrupted by non-coding sequences (introns) which must be excised to generate a translatable mRNA. A dedicated organelle, the spliceosome, is responsible for this process^1^. The spliceosome is a nuclear ribonucleoprotein complex which includes small nuclear RNAs indispensable for its function^2^. These snRNAs interact with three canonical intronic sequence elements, the “donor” at the 5′ splice site, the “acceptor” at the 3′ splice site and the internal sequence element defining the lariat branchpoint adenosine. Two subsequent trans-esterification reactions involving these three sequence elements are necessary for intron excision, which results in the precise fusion of the two bordering exon sequences and the release of the integral intron sequence in the form of a lariat. The major (U2) and the minor (U12) spliceosomes employ functionally analogous but structurally distinct snRNAs to facilitate recognition of different splice sites but the principles of the two-step splicing reaction are essentially the same for both.

Alternative splicing of spliceosomal introns has been described as a means to generate protein diversity^3^. In human, it is estimated that the transcripts of ~ 95% of all multi-exon genes can be alternatively spliced^4^. Different mechanisms – exon skipping, intron retention, the use of alternative 5′ or 3′ splice sites, and mutually exclusive excision of overlapping introns – can lead to different ORFs, derived from the same primary transcript. Moreover, alternative splicing may result in an un-translatable mRNA or lead to a premature termination codon, and as such could constitute a mechanism of regulation of gene expression at the post-transcriptional level, coupling alternative splicing with nonsense-mediated mRNA decay^5^. The question arises to what extent alternative splicing would occur as a common, physiological means of regulation of eukaryotic gene expression or whether the formation of most “nonfunctional” RNA species is merely an inevitable consequence of the inaccuracy inherent to intron excision by the spliceosome^6^.

The intron-exon structure of transcripts of nuclear genes is phylogenetically dynamic as extant introns can be lost and new introns can arise throughout evolution^7^. Complex intervening sequences have been described which consist of more than one U2 intron^8^. One type of complex intervening sequence is made up of abutting U2 introns which are excised by recursive splicing^9,10^. The second type consists of intronic sequences in which (one) U2 intron(s) is/are nested within (an)other U2 intron(s) which can be, but not necessarily are, removed sequentially^11–13^. We have described spliceosomal twin introns (“stwintrons”) in fungi^14–17^. These are complex intervening sequences where the “external” intron can only be removed *after* the excision of the “internal” intron nested within. Stwintrons are thus formally the spliceosomal analogues of the original group II/III twin introns in the *Euglena gracilis* plastid DNA^18^. Nevertheless, stwintrons are basically one particular type of complex intervening sequence consistent of nested U2 introns and there are no reasons to suspect that they would not occur in other eukaryotic kingdoms.

**Figure 1:**
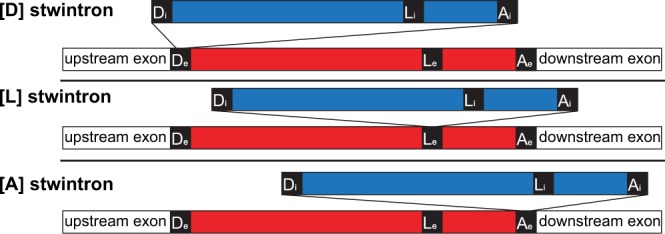
Structure of the three classes of spliceosomal twin introns. The three canonical sequence motifs essential for intron excision by the U2 spliceosome are based on a statistical consensus for each of them in *A*. *nidulans*^23^: 5′-donor [5′-GURWGY], motif around the lariat branchpoint adenosine [5′-RYURAY], and 3′-acceptor [5′-YAG]). In stwintrons, a U2 intron can be located within any of these three canonical intronic sequence elements of the (disrupted) external intron. As a consequence, proper removal of the whole complex intervening sequence can only be accomplished when the internal intron (blue) is excised prior to the external intron (red). We call the three classes of stwintron, [D]-, [L]-, and [A] stwintrons, respectively.

The internal U2 intron of a stwintron can be located within any of the three canonical intronic sequences essential for splicing of the (disrupted) external intron. We have characterised two of the three possible classes of stwintrons (Fig. 1), those where the internal intron interrupts the donor (named [D] stwintrons) or the acceptor ([A] stwintrons) of the external intron^14–17^. We have not published evidence for the existence of the third class, in which the internal intron disrupts the sequence element around the lariat branchpoint adenosine of the external intron ([L] stwintrons).

In higher metazoa (vertebrata in particular) 5′- and 3′ splice sites in the primary transcript are initially paired across each exon (“exon definition”), enabling the existence of large introns alternating with much smaller exons^19,20^. Nevertheless, short introns exist in, e.g., *Drosophila melanogaster*^21^ and zebrafish^22^ and their proper excision is preceded by interactions across the intron (“intron definition”). Moreover, the canonical U2 units of complex intervening sequences in higher metazoa (abutted U2 introns; nested U2 introns)^9,10,12,13^ are subject to intron defining interactions. In ascomycete fungi^23^, almost all introns are small – often <100 nt – and this implies an intron definition mechanism of splicing^24^. In fission yeast (*Schizosaccharomyces pombe*), it was handsomely demonstrated that splice site pairing is essentially confined to intron definition^25,26^. The need for consecutive U2 splicing reactions to excise a stwintron, necessarily “inside out”, demonstrates that splice sites pair via intron definition in filamentous *Ascomycota* (*Pezizomycotina*), too. High-throughput transcriptome analysis has nevertheless shown that alternative splicing occurs frequently in fungi (^27–29^, amongst others). The availability of complete genome sequences of more than a thousand species of *Ascomycota* provides an opportunity to investigate functional aspects of intron gain or -loss across a whole phylum. Here, we describe a new [D5,6] stwintron in the model organism *Aspergillus nidulans*, where the internal intron is situated within the donor element of the external intron, between the fifth and the sixth nucleotide (nt). This stwintron is phylogenetically older than those published previously: it is present across the nine classes of the *Pezizomycotina* subphylum of which member species have been genome-sequenced. We demonstrate that this stwintron can be involved in skipping the downstream exon from the transcript through alternative use of 3′ splice sites for its internal U2 intron. In specific taxa, the same stwintron is embedded in an even more complex intervening sequence that requires four splicing reactions to be excised.

## Results and Discussion

### A novel [D5,6] stwintron is extant in *A*. *nidulans*

**Figure 2:**
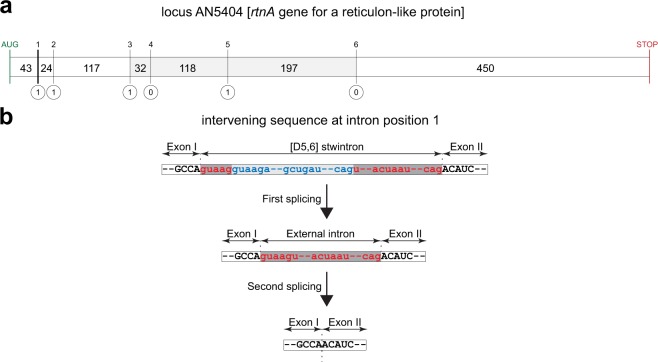
A new stwintron of the [D] class discovered in *A*. *nidulans*. (**a**) The intron-exon structure of the *A*. *nidulans* gene at locus AN5404. Above the bar, the intron positions between the seven exons are numbered 1 to 6, while the phase of the intervening sequences (IS) are given underneath the bar. The start codon is indicated in green and the stop codon in red. The size of the exons is given in nt; for the terminal exons, the given size is the coding section (i.e., exon 1: from AUG to the first IS; exon 7: downstream of the sixth IS until and including the UAA stop codon). This gene model is experimentally confirmed (GenBank MK410458). The intron positions 3, 4, 5, and 6 (i.e., the sizes of exons IV – V – VI) are well conserved in *Pezizomycotina*. In the large majority of taxa, the phase of the intervening sequences at positions 1 and 2 is one. (**b**) Schematic representation of the structure of the phase-1 [D5,6] stwintron in the transcript of the gene at locus AN5404 and its two-step excision by consecutive standard U2 splicing reactions. Exonic sequences are printed in capitals. The internal intron is represented by the light grey bar and its 5′-donor-, lariat branchpoint domain- and 3′-acceptor sequences are printed in blue lettering. The external intron is marked by the darker grey bar; its 5′-donor-, lariat branchpoint domain- and 3′-acceptor sequences are printed in red lettering. Note that the internal intron is nested in the donor element of the external intron between G5 and U6.

**Figure 3:**
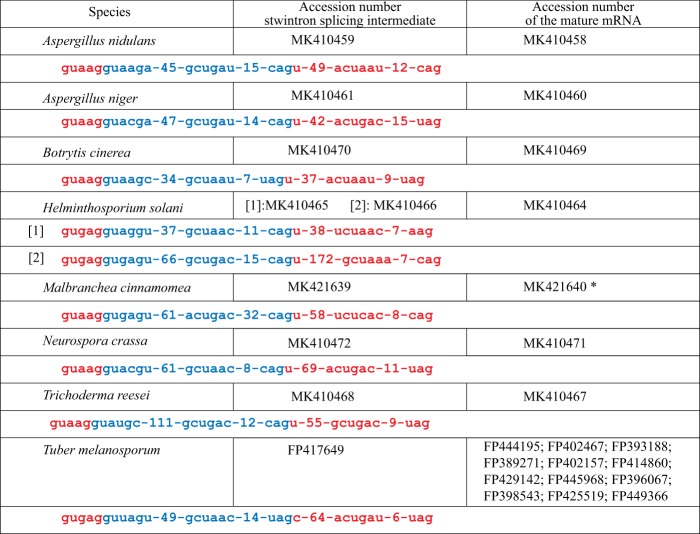
Experimentally confirmed [D5,6] stwintrons in the *rtnA* transcript in five classes of *Pezizomycotina*. The conserved splice site elements of the internal intron (i.e., 5′ donor; element around the lariat branchpoint adenosine; 3′ acceptor) are given in blue letter and those of the external intron are given in red letter. The spacing between the conserved sequence elements is given in nt. In *M*. *cinnamomea*, the [D5,6] stwintron is nested within a bigger complex intervening sequence (see Results and Discussion). Accession MK421640 concerns the RNA species from which the stwintron has been excised, but unlike those for the other species listed, it does not correspond to the mature mRNA of the *M*. *cinnamomea rntA* gene.

We identified a potential [D5,6] stwintron at locus AN5404 of the *A*. *nidulans* genome (http://fungidb.org/fungidb/app/record/gene/AN5404)^30^ in which the internal intron splits the external intron between the fifth and the sixth nucleotide (nt) of the donor sequence (5′-GUAAG|U). The stwintron is the most 5′ intervening sequence in a gene comprising additional five canonical U2 introns (Fig. 2a). We have cloned and sequenced cDNAs of the fully spliced mature mRNA of the gene at locus AN5404 (GenBank MK410458). We detected the postulated splicing intermediate, where the internal intron (75 nt) was excised and where the external intron (76 nt) is still present (GenBank MK410459). The typical two-step excision of this [D5,6] stwintron is shown in Fig. 2b. Figure 3 shows the structure of the AN5404 [D5,6] stwintron in the primary transcript, together with the accession numbers of the sequences of the mature mRNA and of the splicing intermediate.

Locus AN5404 is predicted to encode a reticulon-like protein of 326 amino acids (UniProt C8VGL9; GenBank CBF81965). It is assigned by automated annotation to protein family PF02453 which comprises “proteins of unknown function which associate with the endoplasmic reticulum”. Reticulon-like proteins (Rtn) occur widespread across *Eukarya*^31^. The *A*. *nidulans* RtnA protein is predicted to comprise four transmembrane domains and one coiled-coil domain. The coiled domain (residues 250–300) is clearly detected in the alignment of the 779 orthologues used to infer the protein phylogeny (see below). The termini of the human RTN4 variants were shown to be intrinsically disordered^32^. Orthologues of the reticulon-like protein in the seven species of filamentous fungi experimentally assessed (*A*. *nidulans* and six others, see below) are likewise predicted to feature intrinsically disordered regions at both the N- and C-termini.

### Occurrence of the [D5,6] stwintron in AN5404 orthologues in the *Pezizomycotina* subphylum

We have searched the databases for orthologue genes of the *A*. *nidulans* reticulon-like gene (*rtnA*, locus AN5404) in *Ascomycota*. The [D5,6] stwintron is present in almost all genome-sequenced species of the *Pezizomycotina* subphylum, including in the early divergent classes of the *Pezizomycetes* and the *Orbiliomycetes*. We mined 754 of orthologue genes (and the stwintrons within) from species of *Pezizomycotina* (see Supplementary Table S1). A number of taxa are predicted to have a second [D5,6] stwintron at the position of the second intervening sequence in *A*. *nidulans*. This is the case for many species within the classes of the *Dothideomycetes*, *Xylonomycetes* and *Lecanoromycetes*.

We have experimentally confirmed the [D5,6] stwintron in *Aspergillus niger* (*Eurotiomycetes* class), *Botrytis cinerea* (*Leotiomycetes*), *Trichoderma reesei* and *Neurospora crassa* (the latter two, *Sordariomycetes*) (Fig. 3). In *Helminthosporium solani* (*Dothideomycetes*), we have shown the existence of the two [D5,6] stwintrons predicted in the *rtnA* transcript. In addition, the National Center of Biotechnology Information (NCBI) EST database provided conclusive evidence for the [D5,6] stwintron in *Tuber melanosporum* (*Pezizomycetes*) as we found an expressed sequence tag (EST) covering the transcript of the reticulon-like gene (Accession FP417649) from which the predicted internal intron was absent, while the external intron was still present.

In Supplementary Figure S1, a maximum likelihood phylogeny illustrates the evolutionary relations between the orthologous RtnA proteins, broadly in accordance with taxonomy at the level of classes and orders. Some species (<3%) lack the internal intron of the stwintron; a standard intron is extant at the corresponding position. The patchy distribution of internal intron absence indicates multiple, independent (stw)intron loss events. Although the [D5,6] stwintron at the second *A*. *nidulans* intron position is present in *Pleosporales*, *Hysteriales* and *Botryosphaeriales*, that intron position is not occupied in other *Dothideomycetes* taxa (*Capnodiales*, *Myriangiales*, *Aureobasidium*). The *rtnA* genes of *Sordariomycetes* do not have introns at the third, fourth and fifth *A*. *nidulans* positions (cf. Fig. 2a) but harbour an intron at a new, class-specific position, 14 nt downstream the fourth *A*. *nidulans* intron position. Furthermore, *Orbiliomycetes* and *Pezizomycetes* have an additional intron 70 nt downstream the sixth intron position in *A*. *nidulans*, at a position which is not occupied in species of the *Leotiomyceta* superclass. The most 3′ intron positions (i.e., those within the DNA coding for the autoannotated PF02453 domain) strongly suggest that the reticulon-like genes in several species belonging to the two other *Ascomycota* subphyla are *bona fide* orthologues of *A*. *nidulans rtnA*. Nevertheless, no species of the *Taphrinomycotina* or *Saccharomycotina* subphyla include a [D5,6] stwintron in their reticulon-like gene. We could trace structurally homologous reticulon-like genes in nondikarya taxa *Glomeromycota*, *Mucoromycotina*, and *Mortierellomycotina* – all without stwintron – but not in *Basidiomycota*, the sister phylum of the *Ascomycota*. Once formed, the [D5,6] stwintron appears to be evolutionary stable in the *Pezizomycotina* subphylum and it is the most ancient stwintron described to date.

### The [D5,6] stwintron and the loss of its 3′ neighbouring exon

**Figure 4:**
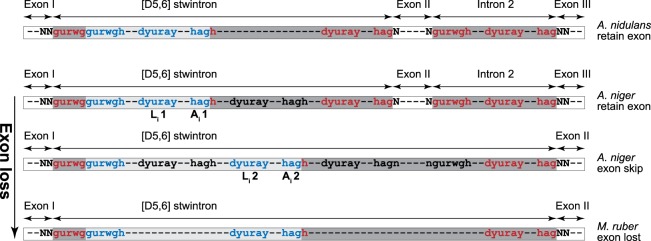
Role for the [D5,6] stwintron in a mechanism of exon skipping and evolutionary exon loss. A model for a mechanism of exon skipping involving a [D] stwintron upstream and a standard U2 intron downstream the skipped exon is presented. Alternative use of 3′ splice sites for the internal intron of the [D5,6] enables the pairing of the 5′ donor of the stwintron’s external intron with the 3′ splice sites of the neighbouring standard intron (intron 2), extending the stwintron to 3′ and incorporating exon II and intron 2 into the excised sequences. The three *Aspergillaceae* species shown are representative of each of the three situations. The *A*. *nidulans* transcript cannot be alternatively spliced due to the absence of alternative 3′ splice sites for the internal intron of its stwintron and always retains exon II (top scheme). The *A*. *niger* transcript, however, can be alternative spliced to include or not, exon II in the mature mRNA (two middle schemes). Finally, in the *Monascus ruber* transcript, loss of *bona fide* internal splice sites has led to intronisation of this “skippable” exon (bottom scheme). In the three species, the length of exon upstream the intron at *A*. *nidulans* position 3 is conserved (i.e., Exon III in the two *Aspergilli*, Exon II in *M*. *ruber*). The internal U2 intron(s) of the stwintron(s) are colour coded in blue letters on a light grey background, the external U2 intron(s) and the standard intron at *A*. *nidulans* position 2 in red letters on a dark grey background. Only the sequences of the canonical conserved splice motifs – 5′-donor [5′-GURWGH], motif around the branchpoint adenosine [5′-DYURAY], 3′-acceptor [5′-HAG]) – are given. Noncoding sequences are in lower case letters. The sequences corresponding to the alternative 3′ acceptors and associated branchpoint elements for the internal intron of the stwintron in *A*. *niger* are annotated as “**L**,**1**” and “**A**,**1**” for the primary stwintron, and “**L**,**2**” and “**A**,**2**” for the 3′ extended secondary stwintron.

The phase-1 intervening sequences at *A*. *nidulans* positions 1 (i.e., the stwintron) and -2 occur in the DNA coding for the N-terminal intrinsically disordered region of the RtnA protein, which is poorly conserved in amino acid sequence and length across the *Pezizomycotina* subphylum. This apparently relaxed context allows natural variations of the local intron-exon structure of the gene. In multiple lineages in the classes of the *Eurotiomycetes*, *Sordariomycetes*, *Orbiliomycetes* and *Pezizomycetes*, the intron at *A*. *nidulans* position 2 has disappeared together with the exon upstream of it. Among the experimentally investigated fungi (Fig. 3) this situation occurs in *T*. *reesei*. The exon downstream the stwintron is 15 nt in the black *Aspergilli* and 24 nt long in all other *Aspergilli* and *Penicillia* (see Supplementary Fig. S2). However, in other genera of the *Aspergillaceae* family, such as *Monascus* and *Xeromyces*, the second exon and the second intron are absent from the *rtnA* gene while one 3′-extended [D5,6] stwintron appears to replace them. In all genome-sequenced species of the *Trichocomaceae* family, the second intron and exon are also absent. In Supplementary Fig. S2, the various lengths of the exon between the stwintron and the intron in position 2 (NB. Here and below we refer to introns and exons in different species by their position in *A*. *nidulans*, cf. Fig. 2a) are given for all analysed species of the *Eurotiales* and *Onygenales* sister orders. In all species of *Eurotiales*, the size of the exon upstream of the position-conserved intron corresponding to intron 3 in *A*. *nidulans*, is (also) conserved. This suggests that the loss of exonic sequences is due to intronisation^33^ of the exon (II) originally between the stwintron and the downstream standard intron corresponding to *A*. *nidulans* intron positions 1 and 2 in an ancestor species. Loss of exonic sequences (in e.g., *Trichocomaceae*, *Monascus* and *Xeromyces*) may have occurred by incorporation of the canonical intron corresponding to that at *A*. *nidulans* position 2 into the external intron of the [D5,6] stwintron, the exon II sequences thus becoming intronic. This evolutionary process can be considered as a phylogenetic analogue (at the DNA level) of an alternative splicing event involving intervening sequences 1 (the [D5,6] stwintron) and -2, resulting in skipping of exon II (Fig. 4).

### Exon skipping in the *rtnA* transcript of *Aspergillus niger*

Below we describe the involvement of the [D5,6] stwintron in alternative splicing of the *rtnA* transcript, leading to skipping of exon II. The alternative splicing event responsible would occur if, after the excision of the internal intron of the [D5,6] stwintron, the second splicing reaction involved the donor of the external intron and acceptor of the downstream canonical intron (intron 2) (Fig. 4). The situation could be rationalised by postulating two alternative [D5,6] stwintrons, one as described above (the primary stwintron; splicing reactions strictly observing intron definition) and the second comprising intervening sequence 1 (i.e., the primary stwintron), exon II and intron 2, to be called the secondary stwintron. The excision of this secondary stwintron would result in the fusion of exons I and III after two splicing reactions, with the consequent loss of exon II.

**Figure 5:**
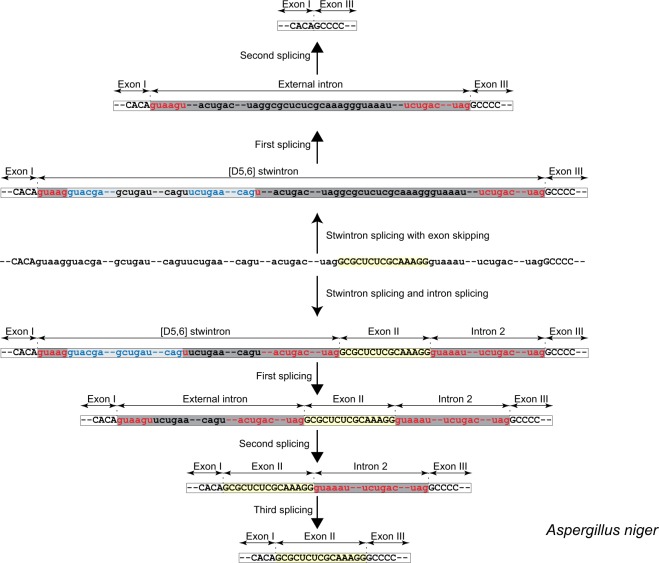
Alternative splicing of the *Aspergillus niger rtnA* transcript. A mechanism of exon skipping involving the [D5,6] stwintron upstream and a standard U2 intron downstream the skipped exon (cf. Fig. 4) is illustrated in *A*. *niger*. Introns and exons are color coded as in Fig. 2b. The relevant part of the primary transcript is depicted in the centre of figure as it can be spliced alternatively, either retaining the exon II (15 nt; highlighted in yellow background) or skipping it. Retention of exon II requires three consecutive standard splicing reactions, all observing splice site pairing by intron definition (downward from the primary transcript). Exon skip (upward from the primary transcript) necessitates two standard splicing reactions, those excising the secondary stwintron, as intron 2 no longer exists as a distinctive intervening sequence when the exon II is skipped. Alternative use of 3′ splice sites for the internal intron of the [D5,6] enables the pairing of the 5′ donor of the stwintron’s external intron with the 3′ splice sites of the neighbouring standard intron (intron 2), extending the stwintron to 3′ and incorporating exon II and intron 2 into the excised sequences (see Results and Discussion section).

We inspected the relevant stwintron sequences in species of *Aspergillaceae* to select an amenable species in which the internal intron of the [D5,6] stwintron could theoretically be excised using an alternative 3′-acceptor downstream the one used to excise the internal intron of the (primary) stwintron. This situation occurs in *A*. *niger*, where a 5′-CAG is located 26 nt downstream of the CAG acceptor of the internal intron of the primary stwintron (Fig. 5). The alternative 3′ splice site could be associated with a distinctive branchpoint element of its own. The secondary internal intron would thus be 29 nt longer at its 3′, and its removal would result in a canonical donor element (5′-GUAAG|U) that cannot effectually pair with the acceptor of the external intron of the primary stwintron, its putative branchpoint element being closer to this alternative donor than to its associated acceptor. The donor of the external intron could however pair with the next available acceptor and associated branchpoint element downstream – those of the standard intron 2 – and the external intron of the secondary [D5,6] stwintron thus would include the sequences of exon II and intron 2. We expect the canonical donor of the secondary external intron (5′-GUAAG|U) to compete with the donor of the standard intron 2, the imperfect (5′-GUAAAU), for the same 3′ splice sites (Fig. 5). The ability to compete with the downstream 5′-donor of intron 2 may be influenced by the association of a protein complex – for instance, the Exon Junction Complex^34,35^ – with the donor of the external intron of the [D5,6] at the exon junction of the internal intron of the stwintron. Employing the same oligonucleotide primers used for the detection of the splicing intermediate of the primary [D5,6] stwintron (cf. Fig. 3), we found a minority of *A*. *niger* cDNA clones (<5%) that lacked the 105-nt long alternative internal intron we predicted (GenBank MK410463). Using a couple of exonic PCR primers, we found cDNA from fully spliced mRNA from which the exon II sequences (15 nt) were indeed absent (GenBank MK410462). The alternative splicing of the *A*. *niger rtnA* transcript was collaborated by extant RNA reads from the NCBI’s Sequence Read Archive (SRA)^36^ (not shown).

### Exon skipping in the *rtnA* transcript of *Neurospora crassa*

**Figure 6:**
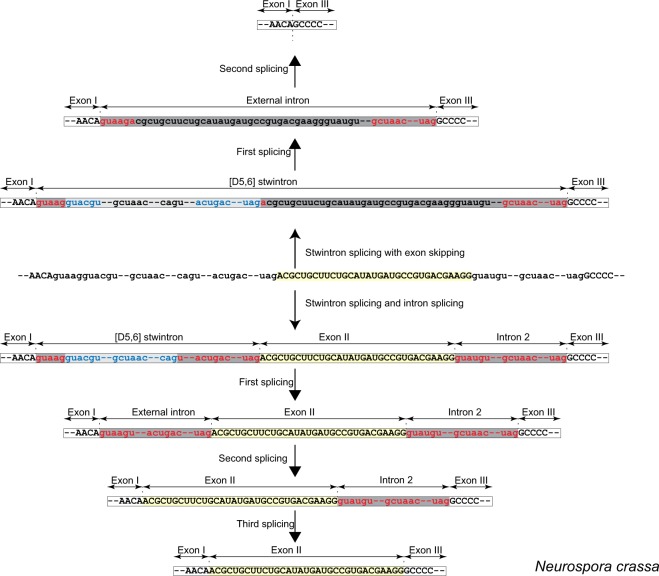
Alternative splicing of the *Neurospora crassa rtnA* transcript. A mechanism of exon skipping involving the [D5,6] stwintron upstream and a standard U2 intron downstream the skipped exon (cf. Fig. 4) is illustrated in *N*. *crassa*. The length of the skipped exon II is 33 nt in *N*. *crassa*. All annotations are like those described in the legend to Fig. 5.

The NCBI databases contain ten ESTs for *N*. *crassa rtnA* where exon II (33 nt) is retained but also three ESTs where it has been skipped (Accession numbers of the latter: GH144932, GH135593 & GH146396). The existence of these ESTs strongly suggest that the *rtnA* transcript is subject to alternative splicing. The likely intermediate of the secondary stwintron excision could be detected amongst the *N*. *crassa* RNA reads in the Sequence Read Archive at NCBI^36^. The 5′ of this alternative internal intron sequence is the donor of the internal intron of the primary [D5,6] stwintron (see Fig. 3) and the 3′ is the acceptor of the external intron of the primary [D5,6] stwintron (Fig. 6), a variation of the situation revealed in *A*. *niger* but with the same end result, skipping of exon II. Supplementary Table S2 provides a list of relevant sequence reads. Its excision reconstitutes a new donor (5′-GUAAG|A) that may pair with the next available acceptor and associated branchpoint element downstream, those of intron 2 in the *rtnA* transcript, to excise the 3′ extended secondary external intron and consequently, would result in exon skipping.

Our results show genuine alternative splicing of the *rtnA* primary transcript in *A*. *niger* and *N*. *crassa*, and strongly suggest a key role of the [D5,6] stwintron in this process.

### Stepwise formation of a functional intron by multiple splicing events in *Onygenales*

The reticulon-like gene in all but two (see below) genome-sequenced species of *Onygenales* (sister order to the *Eurotiales*, the latter includes the *Aspergilli*) harbours a complex intervening sequence at the first intron position (NB. Numbering as in *A*. *nidulans*). In the primary transcript, the [D5,6] stwintron is present and a canonical U2 intron is seen at the second position. The basic gene model for *rtnA* in *Onygenales* is highly reminiscent of that of all the *Aspergilli* (cf. Fig. 2a), with the last four introns at strictly conserved positions, although the stwintron and the second intron are not necessarily phase-1. For almost half of the species, the length of the small exon II is not a multiple of three (see Supplementary Fig. S2). Moreover, 12 of the species where the length of exon II is a multiple of three, the exon contains an in-frame stop codon. In many *Onygenales* species, there is no in-frame start codon upstream of the reticulon domain after excision of the [D6.5] stwintron and the standard intron 2.

**Figure 7:**
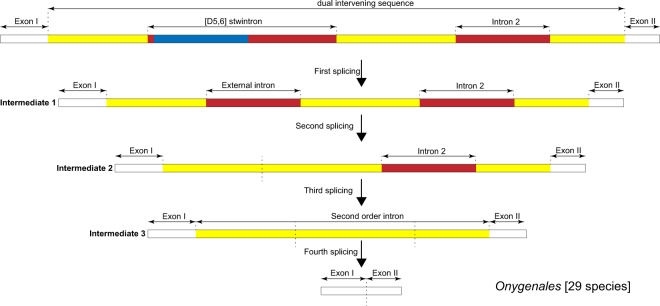
Excision of the first intervening sequence in the primary transcript of the *Onygenales rtnA* genes requires four consecutive U2 splicing reactions. The “dual intervening sequence” includes the [D5,6] stwintron and intron 2 nested within a discontinuous fourth U2 intron – the “second order” intron. The order of U2 removal is depicted schematically from top (primary transcript) to bottom (mature mRNA). The internal U2 intron of the [D5,6] is highlighted in blue and would be the first to be excised to result in splicing intermediate 1. Subsequently, the external U2 intron, highlighted in red, would be removed to yield splicing intermediate 2. After removal of the complete stwintron, the standard intron downstream in the primary transcript (intron 2; also highlighted in red) would be excised and splicing intermediate 3 would be formed. At this stage, the “second order” intron is continuous and enabled, and its excision finally would yield the mature mRNA. The sequences that constitute the second order U2 intron, discontinuous in the primary transcript and the first two splicing intermediates, are highlighted in yellow. Note that the order of the first three standard U2 splicing reactions may deviate from the depicted path.

Two species, *Byssoonygena ceratinophila* and *Ophidiomyces ophiodiicola*, however, have five rather than six introns, all of them canonical. The first intron in these two species is a phase-1 U2 intron which could have resulted from a fusion of the intervening sequences at the first and second position (see below), while the four introns 3′ to it are in the same positions as in the *Aspergilli*. We postulate that in all other *Onygenales* species, a complex intervening sequence can be envisaged encompassing a fully functional [D5,6] stwintron (position 1) as well as the canonical U2 intron downstream (position 2) of it, plus the sequences separating these as well as sequences abutting them at 5′ and 3′, respectively (Fig. 7). The three latter “intronised” sequences would thus arise from ancestral coding sequences. We shall call this extraordinary intervening sequence, a “dual intervening sequence” as it would require multiple, consecutive U2 splicing reactions to be properly excised. In *B*. *ceratinophila* and *O*. *ophiodiicola*, the “dual intervening sequence” may have morphed into one canonical (U2) intron probably after losing its internal “intron-exon” structure, yet retaining its phase. This hypothesis is supported by publicly available expression data in various species of *Onygenales* where the mature mRNA is not interrupted by the “dual intervening sequence” predicted above. These EST and TSA data from NCBI’s Expressed Sequence Tags- and Transcriptome Shotgun Assembly databases, are listed in Supplementary Table S3 and include a TSA for *B*. *ceratinophila rtnA* mRNA that confirms the newly formed standard intron at the first intron position in that species.

We demonstrated experimentally the existence of the new “dual complex intervening sequence” in the reticulon-like gene in *Malbranchea cinnamomea*^37^ (GenBank MK421638). Figure 7 shows schematically that in 29 species of *Onygenales* (including *M*. *cinnamomea*), the excision of the [D5,6] stwintron and the canonical U2 intron at *A*. *nidulans* position 2 necessarily occurs before the formation of a new functional intron by sequential U2 splicing reactions. We have called this new U2 intron, a “second order” intron. In the primary transcript, the “second order” intron would be discontinuous and split in three pieces: its 5′-donor located upstream of the [D5,6] stwintron, its canonical sequence element around the branchpoint adenosine between the [D5,6] stwintron and intron 2, and its 3′-acceptor behind intron 2. Once enabled, the “second order” intron would need to be excised by a fourth (standard) splicing reaction to yield the mature mRNA. In this multi-step splicing sequence, all four consecutive standard splicing reactions are preceded by the definition of a small canonical U2 intron, the default mechanism of splice site selection in *Ascomycota*.

We have experimentally confirmed the existence of all three proposed splicing intermediates and the mRNA end product in *M*. *cinnamomea* (see Supplementary Fig. S3; GenBank MK421639; MK421640; MK421641; MK410473). Variations in the order of U2 splicing are possible, for instance, the standard intron at position 2 (the third U2 intron excised as depicted in Supplementary Fig. S3) could be excised before the removal of the external intron of the [D5,6] stwintron to its 5′ (the second U2 intron excised as depicted in Supplementary Fig. S3) or even before the removal of the internal intron of the stwintron (the first splicing reaction in Supplementary Fig. S3). We cannot exclude that the order of splicing is (occassionally) different as we have not experimentally assessed the alternative patterns, but the last excision must always be that of the “second order” U2 intron. To the best of our knowledge, exon definition^19,20^ has never been described in ascomycete fungi. In the case of the *Onygenales rtnA* transcript, exon defining splice site selection cannot provide an alternative route to excise the entire “dual complex intervening sequence” in one splicing reaction since the three intronic splicing elements of the “second order” U2 intron (5′-donor, element including the lariat branchpoint adenosine, 3′-acceptor) are separated from one another by the canonical U2 introns nested within.

Moreover, we found the proposed “second order” intron as one continuous sequence in three ESTs from *Coccidioides immitis* and *C*. *posadasii* (GenBank GH365468, GH423005; GH438822). In the first two ESTs, all other four canonical introns at the downstream positions (i.e., introns 3–6) are absent, suggesting that the “second order” intron is the last to be removed despite it being (part of) the most 5′ intervening sequence. The third EST (GH438822) still harbours introns 4 and 5, in addition to the “second order” intron. Our results demonstrate the existence of a complex intervening sequence, the “dual intervening sequence”, that can only be properly removed by four consecutive U2 splicing reactions each strictly complying with intron defining splice site selection. U2 introns are not necessarily excised co-transcriptionally, because the “second order” intron in *Onygenales rtnA* does not exist as a functional intron in the primary transcript.

## Conclusions

Analysis of *rtnA* [D5,6] stwintrons in 788 species of fungi revealed novel functional aspects of spliceosomal twin introns. In the order of the *Onygenales*, a new intron has evolved by intronisations around and in between the pre-extant stwintron and the downstream intron-2 in their reticulon-like gene. This new intervening sequence is complex, as the stwintron and the second intron within remain functional and have to be excised, before the remaining intronic sequences could be excised as one standard U2 intron. Our results confirm that U2 introns are not necessarily excised co-transcriptionally.

In various other *Pezizomycotina* taxa, the exon downstream of the [D5,6] stwintron is absent from the *rtnA* gene, with the stwintron seemingly extended 3′ to include the sequences of that exon and of the canonical U2 intron behind it. The apparent intronisation of these exonic sequences can be rationalised as a phylogenetic analogue of the physiological phenomenon of exon skipping in an ancestor species where one of the alternative splice options is lost. The stwintron is implicated in a mechanism of this mode of alternative splicing by its ability to use an alternative acceptor for its internal U2 intron, as we show is the case in *A*. *niger* and *N*. *crassa*, extending the internal intron such that the 5′ donor of the external intron can no longer effectually pair with its conventionally used acceptor (and associated lariat branch point adenosine). Instead, this distal 5′-donor competes for the 3′ splice site of the downstream standard intron.

## Methods

### Fungal strains, cultivation and nucleic acid isolation

The fungi employed in this study and the respective growth media used for biomass formation and nucleic acid extraction are listed online in Supplementary Table S4. All strains were grown in 500-mL Erlenmeyer flasks with 100 mL of growth medium seeded with vegetative spore inocula, in a rotary shaker (Infors HT Multitron) at 200 rotations per min for 24 h. Mycelia were harvested by filtration over sterile Miracloth (Calbiochem). The biomass was washed with distilled water, frozen and ground to powder under liquid nitrogen. For the extraction of genomic DNA and total RNA from deep frozen mycelial powder, Macherey-Nagel NucleoSpin kits (NucleoSpin Plant II and NucleoSpin RNA Plant, respectively) were used.

### Reverse transcription PCR (RT-PCR) and sequencing

Reverse transcription was performed with Oligo(dT) as a primer and 1 μg of total RNA as the template using the First Strand cDNA Synthesis Kit (Thermo Scientific). PCR reactions were done with 4 μL of single strand cDNA template and gene-specific oligonucleotide primers (see Supplementary Table S5) using DreamTaq DNA Polymerase (Thermo Scientific). After initial denaturation at 95 °C for 2 min, 40 amplification cycles of 95 °C for 30 s, 56 °C for 1 min, and 72 °C for 1 min were executed, followed by one post-cyclic elongation at 72 °C for 5 min. Amplified DNA fragments were separated in native agarose gels.

To confirm the existence of the predicted stwintron splicing intermediates, we used primer pairs that do not amplify DNA off mature mRNA template. One of the primers eclipses an intron-exon junction of the putative external intron and contains few nt at its 3′ end that do not basepair when the external intron is absent. This protocol usually yields two PCR fragments of defined sizes of which the smaller one corresponds to the splicing intermediate. Both fragments were processed and sequenced. Thereto cDNA was purified with NucleoSpin Gel & PCR Clean-up (Macherey-Nagel) and subsequently cloned using pGEM-T Easy Vector System I (Promega). Three independent plasmid clones were sequenced over both strands using universal primers (Eurofins Genomics, Ebersberg, Germany). All RT-PCR experiments were done in duplicate, starting with biomass from two independent liquid cultures. Sequences were deposited at GenBank under accession numbers GenBank MK410458–MK410473 and MK421638–MK421641. The same material is also available as Supplementary Sequence Data.

### Identification of putative *A*. *nidulans* stwintrons of the [D5,6] type

The principles of the stwintron sequence motif search in (fungal) whole genome sequence datasets were detailed previously^16^. Recapitulating, we defined five degenerated sequence motifs for the donor-, acceptor- and lariat branch point sequence elements within a stwintron, including the two hybrid motifs characteristic for the stwintron type (i.e., consistent of nucleotides (nt) of the external- as well as of the internal intron). These motifs are based on a statistical consensus for the three conserved intron elements in *A*. *nidulans*^23^ (i.e., donor, 5′-GTRWGY; branch point motif, 5′-RYTRAY; and acceptor, 5′-YAG), although one can use a more relaxed consensus, for instance, at the first position of the 6-nt element around the lariat branch point adenosine (D instead of R), or the first position of the 3-nt acceptor (H instead of Y). Furthermore, we defined distance ranges separating these five motifs conforming four principles rooted in our experience in manually calling intron-exon structure in filamentous fungi: [1], The minimum length of an *A*. *nidulans* intron is 42 nt; [2], The minimum distance between the lariat branch point element and the acceptor element is 4 nt; [3], the distance between the donor element at 5′ and the lariat branch point element is always bigger (and in the majority of cases, considerably bigger) than the distance between the latter and the acceptor at 3′; [4] *bona fide* 5′- and 3′-splice sites are paired across the intron (intron definition) – basically, the paired splice sites are those nearest to one another to facilitate excision of the smallest possible intron^25^ (albeit always subject to principles [1], [2] and [3]). The mean intron length is reported to be 73 nt in *A*. *nidulans* while canonical introns longer than 160 nt are rare^23^. In accordance, we set the distance range between the donor- and lariat branch point elements from 25 to 120 nt. The first stwintrons we reported on were encountered serendipitously^14,15^. However, these simple, degenerated sequence motifs we designed allowed us to actively search for candidate stwintrons in whole genome sequences: a screen for [D1,2] stwintrons led to the identification of two *bona fide* stwintrons^16,17^.

At the onset of the current work, we searched for stwintrons of the [D5,6] type, i.e., where the internal intron is nested within the canonical donor element of the external intron between the latter’s fifth and sixth nt (5′-GTRWG|H). The eight super scaffolds (GenBank BN001301–BN001308) from the re-assembly and re-annotation of the 248 original sequence contigs of the *A*. *nidulans* genome^38^, correspond to the eight chromosomes. De facto, we screened with the following sequence motif using the Sequence Manipulation Suite^39^:

GTRWGGTRWGH(25,120)DYTRAY(4,24)HAGH(25,120)DYTRAY(4,24)HAG

Candidate stwintrons were each manually curated before being subjected to experimental verification, taking into account criteria, like: [1] Whether the sequence separates (known or putative) coding sequences; [2] Whether there are expression data available confirming (or dismissing) its existence; [3] Whether the stwintron can also be predicted (at the same position) in any orthologue gene in related species, like species of the same genus or family, for which genome sequences are publicly available.

### Mining of Ascomycete orthologues of the *A*. *nidulans* reticulon-like (*rtnA*) gene

TBLASTN screens were performed in public DNA databases at the website of the NCBI using the on-line facilities^40^. The 326 amino acids-long protein encoded at *A*. *nidulans* locus AN5404, the seven-exon gene model of which was verified experimentally (GenBank MK410458), was employed as query. We did not use automated annotation results or deduced protein databases; the intron-exon structure was manually called from the collected DNA sequences. The orthologous genes encoding the reticulon-like protein are identified by the contig accession numbers and the coordinates of the coding region (ATG to STOP) provided online in Supplementary Table S1, along with the coordinates of the [D5,6] stwintron(s) and the *Onygenales* “dual intervening sequence” (where appropriate).

### Secondary structure predictions of the reticulon-like protein (RtnA)

Transmembrane helices were predicted online using the TMHMM (version 2) server^41^. Structural disorder was predicted with DISOPRED3^42^ and coiled-coil structures with PCOILS^43^.

### Maximum likelihood phylogenetic analysis

To infer phylogenetic trees, 779 of the collected putative reticulon-like proteins were aligned with MAFFT^44^ (Multiple Alignment using Fast Fourier Transform; version 7), applying the L-INS-i algorithm trained on recognising one conserved domain. To root the trees, nine putative reticulon-like proteins from *Mortierellomycotina* species were included. The MAFFT alignment was trimmed with Block Mapping and Gathering with Entropy software^45^ (BMGE version 1.12), varying the similarity matrix and the block size. A maximum likelihood tree was then calculated by PhyML version 3.0 with automatic substitution model selection^46^ for each of the trimmed alignments generated: in each case, the software selected the LG + G + I substitution model. Branch support was assessed with approximate likelihood ratio tests^47^. Trees were drawn from the Newick output with FigTree (version 1.4.3: http://tree.bio.ed.ac.uk/software/figtree).

### Accession numbers

Accession numbers for sequences determined during this study: GenBank MK410458–MK410473 & MK421638–MK421641.

## Supplementary information

Supplementary Information

## References

[1] Lee Y, Rio DC (2015). Mechanisms and regulation of alternative pre-mRNA splicing. Annu. Rev. Biochem..

[2] Papasaikas P, Valcárcel J (2016). The spliceosome: the ultimate RNA chaperone and sculptor. Trends Biochem. Sci..

[3] Nilsen TW, Graveley BR (2010). Expansion of the eukaryotic proteome by alternative splicing. Nature.

[4] Pan Q, Shai O, Lee LJ, Frey BJ, Blencowe BJ (2008). Deep surveying of alternative splicing complexity in the human transcriptome by high-throughput sequencing. Nat. Genet..

[5] McGlincy NJ, Smith CW (2008). Alternative splicing resulting in nonsense-mediated mRNA decay: what is the meaning of nonsense?. Trends Biochem. Sci..

[6] Saudemont B (2017). The fitness cost of mis-splicing is the main determinant of alternative splicing patterns. Genome Biol..

[7] Carmel L, Wolf YI, Rogozin IB, Koonin EV (2007). Three distinct modes of intron dynamics in the evolution of eukaryotes. Genome Res..

[8] Hafez M, Hausner G (2015). Convergent evolution of twintron-like configurations: One is never enough. RNA Biol..

[9] Burnette JM, Miyamoto-Sato E, Schaub MA, Conklin J, Lopez AJ (2005). Subdivision of large introns in Drosophila by recursive splicing at nonexonic elements. Genetics.

[10] Georgomanolis T, Sofiadis K, Papantonis A (2016). Cutting a long intron short: recursive splicing and its implications. Front. Physiol..

[11] Parra MK, Tan JS, Mohandas N, Conboy JG (2008). Intrasplicing coordinates alternative first exons with alternative splicing in the protein 4.1R gene. EMBO J..

[12] Suzuki H, Kameyama T, Ohe K, Tsukahara T, Mayeda A (2013). Nested introns in an intron: Evidence of multi-step splicing in a large intron of the human dystrophin pre-mRNA. FEBS Lett..

[13] Gazzoli I (2016). Non-sequential and multi-step splicing of the dystrophin transcript. RNA Biol..

[14] Flipphi M, Fekete E, Ág N, Scazzocchio C, Karaffa L (2013). Spliceosome twin introns in fungal nuclear transcripts. Fungal Genet. Biol..

[15] Ág N, Flipphi M, Karaffa L, Scazzocchio C, Fekete E (2015). Alternatively spliced, spliceosomal twin introns in *Helminthosporium solani*. Fungal Genet. Biol..

[16] Fekete E (2017). A mechanism for a single nucleotide intron shift. Nucleic Acids Res..

[17] Flipphi M (2017). Emergence and loss of spliceosomal twin introns. Fungal Biol. Biotechnol..

[18] Copertino DW, Hallick RB (1993). Group II and group III introns of twintrons: potential relationships with nuclear pre-mRNA introns. Trends Biochem. Sci..

[19] Berget SM (1995). Exon recognition in vertebrate splicing. J. Biol. Chem..

[20] De Conti L, Baralle M, Buratti E (2013). Exon and intron definition in pre-mRNA splicing. WIREs RNA.

[21] Talerico M, Berget SM (1994). Intron definition in splicing of small *Drosophila* introns. Mol. Cell. Biol..

[22] Fernandez JP (2018). RES complex is associated with intron definition and required for zebrafish early embryogenesis. PLoS Genet..

[23] Kupfer DM (2004). Introns and splicing elements of five diverse fungi. Eukaryot. Cell.

[24] Collins L, Penny D (2005). Investigating the intron recognition mechanism in eukaryotes. Mol. Biol. Evol..

[25] Romfo CM, Alvarez CJ, van Heeckeren WJ, Webb CJ, Wise JA (2000). Evidence for splice site pairing via intron definition in *Schizosaccharomyces pombe*. Mol. Cell. Biol..

[26] Shao W, Kim HS, Cao Y, Xu YZ, Query CC (2012). A U1-U2 snRNP interaction network during intron definition. Mol. Cell. Biol..

[27] Wang B (2010). Survey of the transcriptome of *Aspergillus oryzae* via massively parallel mRNA sequencing. Nucleic Acids Res..

[28] Grützmann K (2014). Fungal alternative splicing is associated with multicellular complexity and virulence: a genome-wide multi-species study. DNA Res..

[29] Xie BB (2015). Deep RNA sequencing reveals a high frequency of alternative splicing events in the fungus *Trichoderma longibrachiatum*. BMC Genomics.

[30] Cerqueira GC (2014). The *Aspergillus* Genome Database: multispecies curation and incorporation of RNA-Seq data to improve structural gene annotations. Nucleic Acids Res..

[31] Oertle T, Klinger M, Stuermer CA, Schwab ME (2003). A reticular rhapsody: phylogenic evolution and nomenclature of the *RTN/Nogo* gene family. FASEB J..

[32] Li M, Son J (2007). The N- and C-termini of the human Nogo molecules are intrinsically unstructured: Bioinformatics, CD, NMR characterization, and functional implications. Proteins.

[33] Irimia M (2008). Origins of introns by “intronization” of exonic sequences. Trends Genet..

[34] Hauer C (2016). Exon junction complexes show a distributional bias toward alternatively spliced mRNAs and against mRNAs coding for ribosomal proteins. Cell Rep..

[35] Woodward LA, Mabin JW, Gangras P, Singh G (2017). The exon junction complex: a lifelong guardian of mRNA fate. WIREs RNA.

[36] Kodama Y, Shumway M, Leinonen R (2012). The sequence read archive: explosive growth of sequencing data. Nucleic Acids Res..

[37] Morgenstern I (2012). A molecular phylogeny of thermophilic fungi. Fungal Biol..

[38] Wortman JR (2009). The 2008 update of the *Aspergillus nidulans* genome annotation: a community effort. Fungal Genet. Biol..

[39] Stothard P (2000). The sequence manipulation suite: JavaScript programs for analyzing and formatting protein and DNA sequences. Biotechniques.

[40] Altschul SF (1997). Gapped BLAST and PSI-BLAST: a new generation of protein database search programs. Nucleic Acids Res..

[41] Krogh A, Larsson B, von Heijne G, Sonnhammer ELL (2001). Predicting transmembrane protein topology with a hidden Markov model: application to complete genomes. J. Mol. Biol..

[42] Jones DT, Cozzetto D (2015). DISOPRED3: precise disordered region predictions with annotated protein-binding activity. Bioinformatics.

[43] Gruber M, Söding J, Lupas AN (2006). Comparative analysis of coiled-coil prediction methods. J. Struct. Biol..

[44] Katoh K, Standley DM (2013). MAFFT multiple sequence alignment software version 7: improvements in performance and usability. Mol. Biol. Evol..

[45] Criscuolo A, Gribaldo S (2010). BMGE (Block Mapping and Gathering with Entropy): a new software for selection of phylogenetic informative regions from multiple sequence alignments. BMC Evol. Biol..

[46] Lefort V, Longueville JE, Gascuel O (2017). SMS: Smart Model Selection in PhyML. Mol Biol Evol..

[47] Anisimova M, Gascuel O (2006). Approximate likelihood-ratio test for branches: a fast, accurate, and powerful alternative. Syst. Biol..

