# Supplementary material for: A spliceosomal twin intron (stwintron) participates in both exon skipping and evolutionary exon loss
Source: Sci Rep. 2019 Jul 9;9:9940. doi: 10.1038/s41598-019-46435-x (PMC6616335; doi:10.1038/s41598-019-46435-x)
Supplement: Supplementary file 1 — Supplementary Information [file 41598_2019_46435_MOESM1_ESM.pdf]

Supplementary information

(Manuscript number: SREP-19-14466A; Scientific Reports 2019)

A spliceosomal twin intron (stwintron) participates in both exon skipping and evolutionary exon loss

Napsugár Kavalecz [1], Norbert Ág [1], Levente Karaffa [1], Claudio Scazzocchio [2,3], Michel Flipphi [1] & Erzsébet Fekete [1],*

[1] Department of Biochemical Engineering, University of Debrecen, Debrecen, 4032, Hungary
[2] Department of Microbiology, Imperial College London, London, SW7 2AZ, UK
[3] Institut de Biologie Intégrative de la Cellule, Centre National de la Recherche Scientifique, Unité Mixte de Recherche 9198, Gif-sur-Yvette, 91405, France

* To whom correspondence should be addressed. Email: kicsizsoka@yahoo.com

Supplementary Figure S1
Supplementary Figure S2
Supplementary Figure S3
Supplementary Table S1
Supplementary Table S2
Supplementary Table S3
Supplementary Table S4
Supplementary Table S5
Supplementary Sequence Data

Supplementary Figure S1

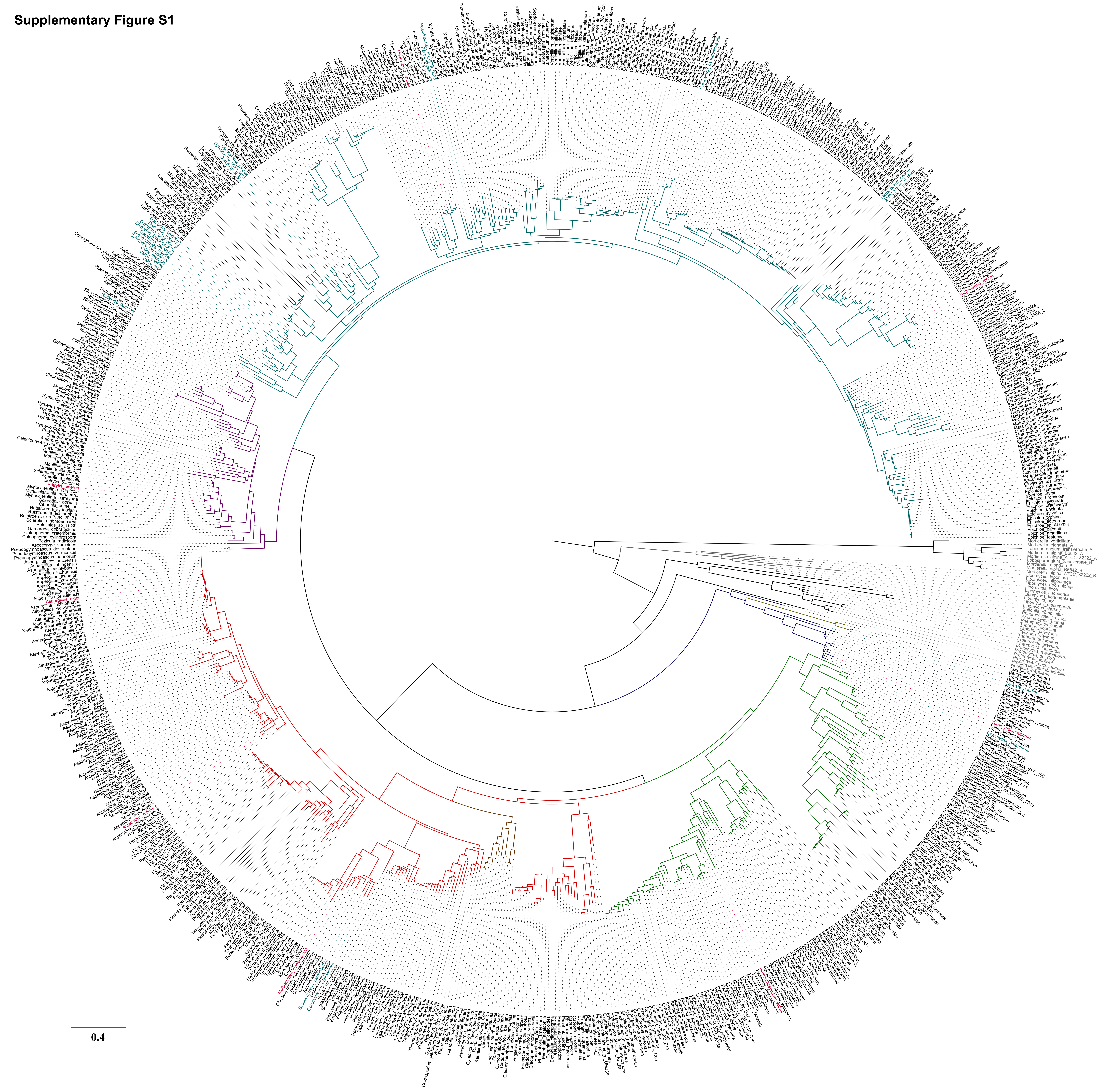

Supplementary Figure S1. Phylogenetic tree of the reticulon-like protein of the *Pezizomycotina*. The analysis included 779 proteins from species of Ascomycota for which genome sequences were freely available at NCBI (October 2018). Nine homologous proteins from species of *Mortierellomycotina* (NB. A different phylum) provide an outgroup. Alignment carried out with MAFFT - L-INS-I, using a BLOSUM 62 scoring matrix. This alignment was trimmed to 217 informative residues with BMGE using a BLOSUM 50 similarity matrix and a block size of 4. For the complete methodology, see Material and Methods section. For clarity, the node statistics (Approximate Likelihood-Ratio Tests) were omitted from the Figure. The main branches are color coded to distinguish between the classes of *Pezizomycotina*: *Pezizomycetes*, blue; *Orbiliomycetes* (three species), olive green; *Dothideomycetes*, green; *Eurotiomycetes*, red; *Lecanoromycetes*, brown; *Leotiomycetes*, purple; and *Sordariomycetes*, cyan. The branches at the basis of the Maximum Likelihood tree with orthologue proteins from sixteen *Taphrinomycotina* and nine *Lipomyces (Saccharomycotina)* species, as well as the outgroup with the *Mortierellomycotina* homologues, are drawn in grey and the species names are printed in grey tone. None of the genes in these latter three fungal taxa carry a [D5,6] stwintron. The names of the *Pezizomycotina* species for which the existence of the [D5,6] stwintron was experimentally confirmed (see Results and Discussion, Fig. 3), are highlighted in red. The species from which the stwintron in the *rtnA* gene was absent (i.e., one species of *Pezizomycetes*, one species of *Dothideomycetes*, two species of *Eurotiomycetes* and 18 species in four different orders of *Sordariomycetes*; together < 3 % of the *Pezizomycotina* species), in cyan. Note that the RtnA proteins from *Xylona heveae* and *Symbiotaphrina buchneri* (*Xylonomycetes* class), *Arthonia radiata* (*Arthoniomycetes* class), *Chaetomium thermophilum* (*Sordariomycetes*, *Sordariales*, *Chaetomiaceae*) and *Ascosphaera apis* (*Eurotiomycetes*, *Ascosphaeriales*), were not included due to possible long branch attraction effects. Nonetheless, the five encoding genes (Table S3) do feature a [D5,6] stwintron and in case of the two *Xylonomycetes*, also a second [D5,6] stwintron.

Supplementary Figure S2

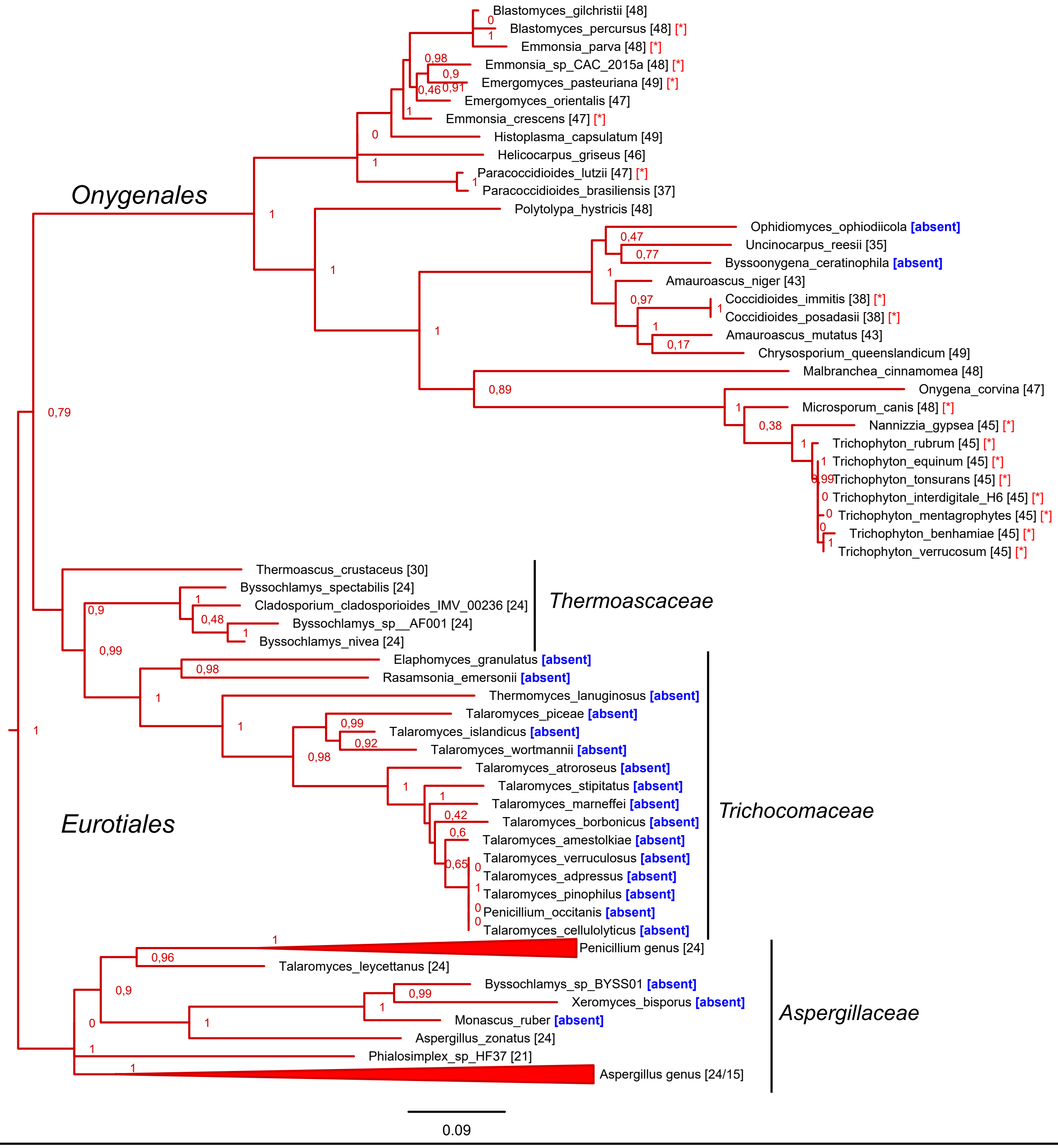

Supplementary Figure S2. Evolution of the second exon in the gene for a reticulon-like protein (RtnA) in the subclass of the *Eurotiomycetidae*. The Figure shows a phylogenetic tree of RtnA in the *Eurotiomycetidae* – comprising the sister orders *Eurotiales* and *Onygenales* – extracted from the Maximum Likelihood phylogeny of the reticulon-like protein of *Pezizomycotina* (for a circular representation of the latter, see Figure S1). Approximate Likelihood-Ratio Tests (aLTR) provide node statistics. In the order of the *Eurotiales* (lower branches), three families with genome-sequenced species are indicated. The clades for the *Aspergillus* and *Penicillium* genera, each harbouring dozens of species (see Table S2), are collapsed. Following the name of the fungus (or the genus), the size of the second exon is given between square brackets: in *A. nidulans*, this is the small exon (24 nt) between the [D5,6] stwintron and the second intron (Figure 2A). In most taxa of *Pezizomycotina* (but in not all), the size of this exon II is either a multiple of three or it ceased to exist after being absorbed into a 3' extended [D5,6] stwintron (see text). Where the exon corresponding to *Aspergillus* exon II is absent, this is indicated by **[absent]** following the species name. In all species of *Eurotiales*, the phase of the stwintron is one. In all *Penicillium* species, the size of exon II is 24 nt. In *Aspergillus* species, its length is also 24 nt, except in all species of section *Nigri* and in *A. sclerotiorum*, *A. persii* and *A. steynii*, where it is 15 nt. In the *Onygenales* order (top branch), in almost half of the species (14) the size of exon II is no longer a multiple of three. In the (15) species where the size remains a multiple of three (either 48 or 45 nt), all but 3 species feature a premature stop codon in exon II, in frame with the reticulon domain (after splicing). The presence of such stop codon has been indicated by **[*]** following the species name. In two species of *Onygenales*, *B. ceratinophila* and *O. ophiodiicola*, the exon corresponding to the *A. nidulans* exon II is absent and the stwintron (intron1) and intron 2 are replaced by a new canonical intron separating exon 1 and the new exon 2 (corresponding to exon 3 in other species). The lack of an exon corresponding to *A. nidulans* exon II in these two species has been indicated by **[absent]**.

Supplementary Figure S3

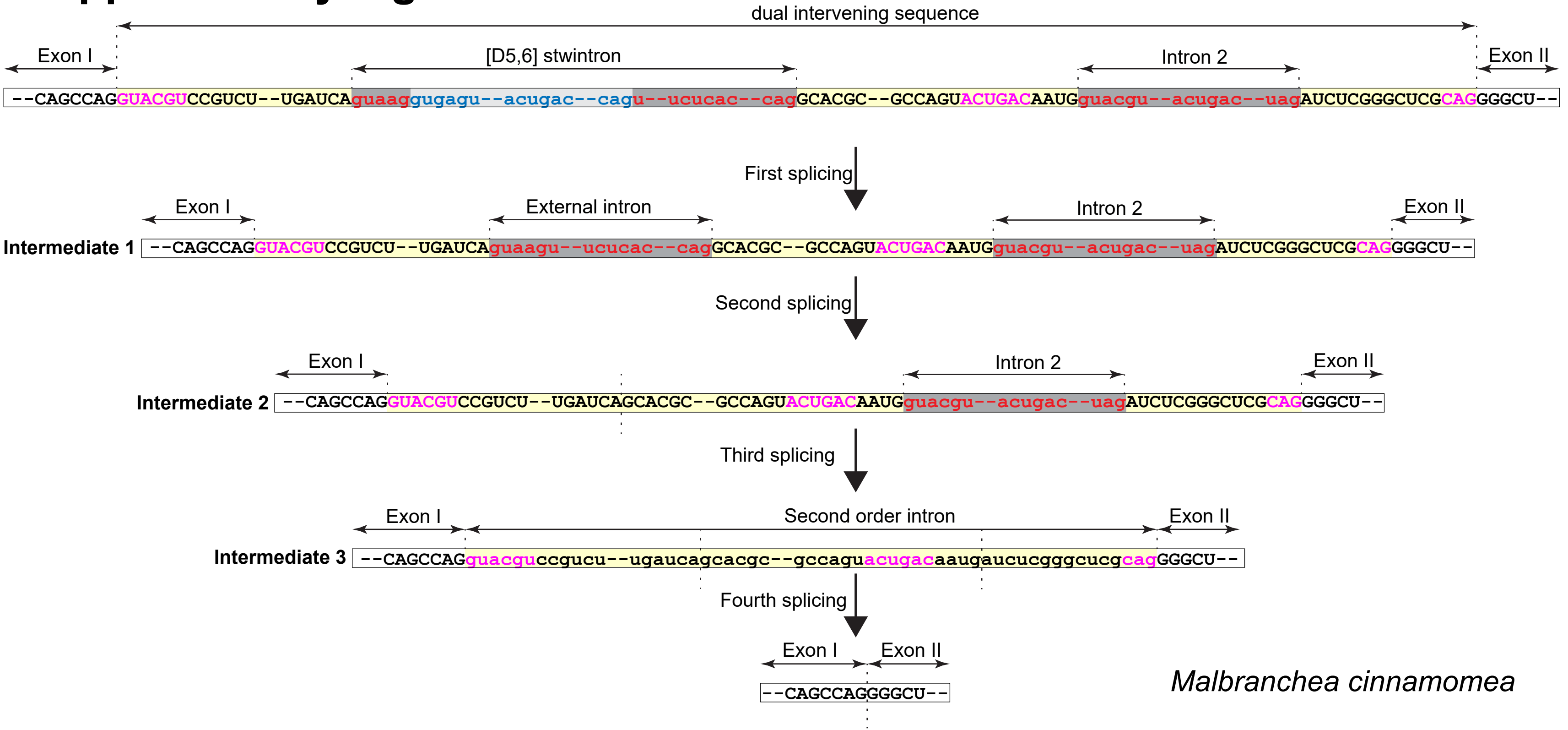

Supplementary Figure S3. Excision of the first intervening sequence in the primary transcript of the *Malbranchea cinnamomea rtnA* gene requires four consecutive U2 splicing reactions. The "dual intervening sequence" includes the [D5,6] stwintron and intron 2 nested within a discontinuous fourth U2 intron – the "second order" intron. This figure is essentially identical to Fig. 7 in the main manuscript, apart from the sequences of the relevant canonical splice elements of each constituent U2 intron (donor, element around lariat branchpoint adenosine, acceptor) specific for *Malbranchea cinnamomea*, the fungus that we verified experimentally. The sequences that constitute the second order U2 intron, discontinuous in the primary transcript and the first two splicing intermediates, are highlighted in yellow.

Supplementary Table S1. Identification of orthologous Ascomycete *rtnA* genes (accession; coordinates AUG-STOP) and the [D5,6] stwintrons and dual intervening sequences within

Name	Taxon	Accession number	Coordinates gene	Coordinates [D5,6] stwintron	Coordinates 2nd [D5,6] stwintron	Coordinates of "dual" i. s.
Aciculosporium take	Sordariomycetes	AFQZ01000098	42379-43830	42431-42581		
Acidomyces richmondensis	Dothideomycetes	JPDO01000024	c81202-79865	c81120-80865		
Acremonium chrysogenum	Sordariomycetes	JPKY01000007	150358-151655	150401-150525		
Acremonium furcatum	Sordariomycetes	BCIA01000005	c3726318-3724895	c3726242-3726063		
Albophoma yamanashiensis	Sordariomycetes	BCKH01000002	2755143-2756479	2755186-2755311		
Alternaria alternata	Dothideomycetes	LMXP01000005	414549-416011	414727-414845	414918-415018	
Alternaria arborescens	Dothideomycetes	AIIC01000013	84850-86314	85028-85146	85219-85319	
Alternaria brassicicola	Dothideomycetes	PHFN01000013	350797-352251	350975-351096	351169-351271	
Alternaria consortialis	Dothideomycetes	BCGG01000005	371995-373456	372173-372290	372363-372463	
Alternaria consortialis (paralogue)	Dothideomycetes	BCGG01000001	c1742519-1740894			
Alternaria solani	Dothideomycetes	JRWV01000099	162036-163482	162214-162331	162404-162504	
Amauroascus mutatus	Eurotiomycetes	LJPJ01001801	c67028-65472	c66897-66747		c66988-66624
Amauroascus niger	Eurotiomycetes	LJPK01000444	c2837-1168	c2675-2512		c2797-2371
Ambrosiella xylebori	Sordariomycetes	PCDO01000011	1003979-1005459	1003998-1004423		
Amorphotheca resinae	Leotiomycetes	JZSE01000081	12071-13390	12171-12277		
Annulohypoxylon stygium	Sordariomycetes	QLPL01000010	c2257187-2255801	c2257126-2256945		
Aquanectria penicillioides	Sordariomycetes	PYIU01000001	c5511170-5510165	c5511537-5511374		
Arthonia radiata	Arthoniomycetes	PSQN01000017	3463662-3465110	3463801-3463905		
Arthrobotrys oligospora	Orbiliomycetes	ADOT01000192	c155408-154274	c155344-155167		
Articulospora tetracladia	Leotiomycetes	PYIV01000008	661524-662824	661618-661730		
Ascobolus immersus	Pezizomycetes	PZQT01000108	c106216-104811	c106086-105922		
Ascochyta rabiei	Dothideomycetes	JYNV01000015	c234081-232625	c233915-233795	c233722-233612	
Ascocoryne sarcoides	Leotiomycetes	AIAA01000029	c21210-19957	c21128-21016		
Ascosphaera apis	Eurotiomycetes	AZGZ01000005	737972-739443	737570-737871		737417-737871
Aspergillus aculeatinus	Eurotiomycetes	PSTE01000021	c274739-273264	c274699-274547		
Aspergillus aculeatus	Eurotiomycetes	MRCK01000148	c348314-346840	c348274-348122		
Aspergillus arachidicola	Eurotiomycetes	NEXV01000715	91892-93356	91947-92086		
Aspergillus awamori	Eurotiomycetes	BCGT01000003	3602603-3604046	3602640-3602787		
Aspergillus bombycis	Eurotiomycetes	LYCR01000083	117569-119034	117624-117763		
Aspergillus brasiliensis	Eurotiomycetes	LJXV01000041	c287329-285886	c287292-287145		
Aspergillus brunneoviolaceus	Eurotiomycetes	PSTC01000060	c234031-232557	c233991-233839		
Aspergillus calidoustus	Eurotiomycetes	CDMC01000015	c366060-364659	c366011-365858		
Aspergillus campestris	Eurotiomycetes	MSFM01000012	c217736-216269	c217681-217553		
Aspergillus candidus	Eurotiomycetes	PKFS01000093	119713-121180	119768-119901		
Aspergillus carbonarius	Eurotiomycetes	AHIG01000404	c1897-460	c1860-1719		
Aspergillus chevalieri	Eurotiomycetes	BCIE01000017	176708-178113	176754-176892		
Aspergillus clavatus	Eurotiomycetes	AAKD03000052	15418-16972	15473-15653		
Aspergillus costaricaensis	Eurotiomycetes	PSTH01000079	516822-518266	516859-517006		
Aspergillus cristatus	Eurotiomycetes	JXNT01000001	190410-191808	190456-190594		
Aspergillus ellipticus	Eurotiomycetes	PSSY01000399	106335-107772	106372-106507		
Aspergillus eucalypticola	Eurotiomycetes	MSFU01000004	c98802-97360	c98765-98618		
Aspergillus fijiensis	Eurotiomycetes	PSTG01000054	31371-32847	31411-31563		
Aspergillus fischeri	Eurotiomycetes	AAKE03000008	1061210-1062654	1061250-1061373		
Aspergillus flavus	Eurotiomycetes	AAIH02000198	5779-7243	5834-5973		
Aspergillus fumigatus	Eurotiomycetes	AAHF01000006	2164997-2166439	2165037-2165160		
Aspergillus glaucus	Eurotiomycetes	LSTL01000222	c68703-67284	c68657-68497		
Aspergillus hancockii	Eurotiomycetes	MBFL01000118	19903-21350	19958-20103		
Aspergillus heteromorphus	Eurotiomycetes	MSFL01000005	582267-583708	582304-582444		
Aspergillus homomorphus	Eurotiomycetes	PSTJ01000050	334837-336310	334877-335032		
Aspergillus ibericus	Eurotiomycetes	PSTI01000029	173115-174552	173152-173293		
Aspergillus indologenus	Eurotiomycetes	PSTB01000054	338438-339912	338478-338630		
Aspergillus japonicus	Eurotiomycetes	PSTF01000011	30249-31723	30289-30441		
Aspergillus kawachii	Eurotiomycetes	BACL01000036	33544-34987	33581-33728		
Aspergillus lacticoffeatus	Eurotiomycetes	MSFR01000079	6071-7515	6108-6255		
Aspergillus lentulus	Eurotiomycetes	BCLY01000001	c453047-451599	c453007-452883		
Aspergillus luchuensis	Eurotiomycetes	MRBP01000040	709728-711172	709765-709912		
Aspergillus mulundensis	Eurotiomycetes	PVWQ01000013	c358257-356839	c358214-358063		
Aspergillus neoellipticus	Eurotiomycetes	QDFW01000123	c257930-256488	c257890-257767		
Aspergillus neoniger	Eurotiomycetes	MSFP01000054	c159664-158222	c159627-159480		
Aspergillus nidulans	Eurotiomycetes	BN001305	c2660331-2658925	c2660288-2660138		
Aspergillus niger	Eurotiomycetes	ACJE01000021	2069616-2071060	2069653-2069800		
Aspergillus nomius	Eurotiomycetes	JNOM01000169	c47035-45575	c46980-46841		
Aspergillus novofumigatus	Eurotiomycetes	MSZS01000003	c444372-442954	c444332-444208		
Aspergillus ochraceoroseus	Eurotiomycetes	JYKN01001674	c18459-16976	c18410-18255		
Aspergillus parasiticus	Eurotiomycetes	JMUG01001200	7523-8987	7578-7717		
Aspergillus persii	Eurotiomycetes	NGZO01000003	4183546-4185018	4183601-4183760		
Aspergillus phoenicis	Eurotiomycetes	QQUR01000001	c764463-763019	c764426-764279		
Aspergillus piperis	Eurotiomycetes	PSTD01000067	32579-34023	32616-32763		
Aspergillus pseudoterreus	Eurotiomycetes	PIJX01000035	c359904-358490	c359861-359702		
Aspergillus rambellii	Eurotiomycetes	JZBS01003801	28712-30195	28761-28917		
Aspergillus ruber	Eurotiomycetes	AWRT01000077	59620-61035	59666-59825		
Aspergillus saccharolyticus	Eurotiomycetes	MSFQ01000041	c789725-788260	c789685-789533		
Aspergillus sclerotiicarbonarius	Eurotiomycetes	PSSZ01000013	c108344-106908	c108307-108168		
Aspergillus sclerotioniger	Eurotiomycetes	MSFK01000004	c686996-685560	c686959-686818		
Aspergillus sclerotiorum	Eurotiomycetes	CBWZ010000477	c5310-3848	c5255-5096		
Aspergillus sojae	Eurotiomycetes	BACA01000038	29590-31054	29645-29784		
Aspergillus sp. MA 6037	Eurotiomycetes	QAGH01001246	873-2292	919-1060		
Aspergillus sp. MA 6041_1	Eurotiomycetes	QAGJ01000163	6664-8083	6710-6851		
Aspergillus sp. MA 6041_2	Eurotiomycetes	QAGJ01000357	c27641-26189	c27595-27440		
Aspergillus steynii	Eurotiomycetes	MSFO01000003	c535697-534225	c535642-535479		
Aspergillus sydowii	Eurotiomycetes	MRCH01000314	70253-71679	70299-70441		
Aspergillus taichungensis	Eurotiomycetes	PKFW01000005	c13218-11736	c13163-13018		
Aspergillus terreus	Eurotiomycetes	AAJN01000098	80277-81690	80320-80481		
Aspergillus thermomutatus	Eurotiomycetes	NKHU01000078	c53101-51638	c53049-52928		
Aspergillus tubingensis	Eurotiomycetes	LJXU01000006	32527-33970	32564-32711		
Aspergillus turcosus	Eurotiomycetes	NIDN01000139	59532-60978	c75958-75833		
Aspergillus unguis	Eurotiomycetes	QLOF01000004	c923018-921635	c922975-922841		
Aspergillus ustus	Eurotiomycetes	JOMC01000028	120991-122400	121040-121192		
Aspergillus uvarum	Eurotiomycetes	MSFT01000026	c397052-395577	c397012-396860		
Aspergillus vadensis	Eurotiomycetes	MSFS01000027	180186-181629	180223-180370		
Aspergillus versicolor	Eurotiomycetes	MRBN01000068	c1149214-1147789	1149168-1149027		
Aspergillus violaceofuscus	Eurotiomycetes	PSTA01000300	13621-15095	13661-13813		
Aspergillus welwitschiae	Eurotiomycetes	QQZQ01000008	c207546-206102	c207509-207362		
Aspergillus wentii	Eurotiomycetes	LJSE01000002	c374329-372882	c374292-374162		
Aspergillus westerdijkiae	Eurotiomycetes	LKBE01000014	c480040-478576	c479985-479835		
Atkinsonella hypoxylon	Sordariomycetes	JFHB01000012	322866-324250	322918-323068		
Atkinsonella texensis	Sordariomycetes	LBNC01000604	c50799-49411	c50747-50599		
Aureobasidium melanogenum	Dothideomycetes	AYEN01000042	c78779-77152	c78691-78438		
Aureobasidium namibiae	Dothideomycetes	AYEM01000016	c149683-148015	c149595-149361		
Aureobasidium pullulans	Dothideomycetes	AYEO01000026	111040-112704	111107-111361		
Aureobasidium pullulans	Dothideomycetes	AMCU01000012	c58632-57007	c58544-58291		
Aureobasidium subglaciale	Dothideomycetes	AYYB01000003	1321797-1323359	1321864-1322117		
Balansia obtecta	Sordariomycetes	JFZS01000288	13380-14789	13432-13593		
Basipetospora chlamydospora	(Sordariomycetes)	BCHP01000003	4743713-4745369	4743774-4743959		
Baudoinia panamericana	Dothideomycetes	AEIF01000002	c486788-485379	c486667-486403		
Beauveria bassiana	Sordariomycetes	ANFO01000134	35953-37241	36014-36014		
Beauveria pseudobassiana	Sordariomycetes	LUDX01000486	133756-135045	133817-133946		
Beauveria rudraprayagi	Sordariomycetes	JMNB01000177	c33261-31973	c33200-33072		
Beverwykella pulmonaria	Dothideomycetes	BCHH01000003	c3990471-3988961	c3990335-3990220	c3990135-3990025	
Bipolaris cookei	Dothideomycetes	NRSV01000023	c106601-105181	c106450-106334	c106261-106158	
Bipolaris cookei (paralogue)	Dothideomycetes	NRSV01000073	c115621-113996			
Bipolaris maydis	Dothideomycetes	AIDY01000013	c586510-585046	c586332-586214	c586141-586034	
Bipolaris maydis (parlogue)	Dothideomycetes	AIDY01000023	639207-641147			
Bipolaris oryzae	Dothideomycetes	AMCO01000286	c4425-2953	c4247-4127	c4054-3943	
Bipolaris oryzae (paralogue)	Dothideomycetes	AMCO01000127	c84046-82394			
Bipolaris sorokiniana	Dothideomycetes	AEIN01000052	c13885-12414	c13707-13583	c13510-13403	
Bipolaris sorokiniana (paralogue)	Dothideomycetes	AEIN01000125	c99820-98276			
Bipolaris victoriae	Dothideomycetes	AMCY01000012	48997-50457	49175-49293	49366-49474	
Bipolaris victoriae (paralogue)	Dothideomycetes	AMCY01000168	c2467-805			
Bipolaris zeicola	Dothideomycetes	AMCN01000190	c46258-44798	c46080-45962	c45889-45781	
Bipolaris zeicola (paralogue)	Dothideomycetes	AMCN01000421	11150-12800			
Blastomyces gilchristii	Eurotiomycetes	ACBU01001327	c11809-10057	c11695-11522		c11766-11373
Blastomyces percursus	Eurotiomycetes	LGTZ01000256	7607-9397	7721-7896		7650-8041
Blumeria graminis f. sp. hordei	Leotiomycetes	AOLT01015339	423-1620	439-550		
Blumeria graminis f. sp. tritici	Leotiomycetes	ASJL01023808	2888-4087	2904-3015		
Botryosphaeria dothidea	Dothideomycetes	MDSR01000006	c139281-137588	c139112-138955	c138894-138771	
Botrytis cinerea	Leotiomycetes	AAID02000875	c192041-190772	c191953-191837		
Botrytis paeoniae	Leotiomycetes	LBGX01000611	20422-21699	20510-20634		
Bretziella fagacearum	Sordariomycetes	MKGJ01000142	38200-39899	38219-38838		
Byssochlamys nivea	Eurotiomycetes	QEIL01000017	222520-223929	222572-222711		
Byssochlamys sp. AF001	Eurotiomycetes	PNEM01000003	c247097-245649	c247045-246907		
Byssochlamys sp. BYSS01	Eurotiomycetes	NIXA01000035	c44985-43557	c44957-44725		
Byssochlamys spectabilis	Eurotiomycetes	BAUL01000041	c201501-200066	c201449-201309		
Byssoonygena ceratinophila	Eurotiomycetes	LJPH01000488	c59242-57946			
Cadophora malorum	Leotiomycetes	FKJQ01000104	26543-27831	26640-26750		
Cadophora sp. DSE1049	Leotiomycetes	PCYN01000015	c142114-140828	c142017-141906		
Cairneyella variabilis	Leotiomycetes	AYLM01001788	41911-43204	42005-42117		
Calonectria leucothoes	Sordariomycetes	NAJI01000080	75196-76190	74851-75048		
Calonectria naviculata	Sordariomycetes	NAGG01000445	c5940-4941	c6278-6104		
Calonectria pseudonaviculata	Sordariomycetes	JYJY01000271	7737-8729	7413-7593		
Calonectria pseudoreteaudii	Sordariomycetes	MOCD01000630	c63497-62503			
Calycina herbarum	Leotiomycetes	LLEY01000018	559749-561083	559852-559974		
Capronia coronata	Eurotiomycetes	AMWN01000003	c132818-131346	c132700-132587		
Capronia epimyces	Eurotiomycetes	AMGY01000006	94231-95662	94355-94474		
Cenococcum geophilum	Dothideomycetes	LKKR01000010	c1015097-1013546	c1014964-1014846	c1014773-1014660	
Ceratocystiopsis brevicomis	Sordariomycetes	PCDN01000003	c1197341-1195931	c1197292-1197143		
Ceratocystiopsis minuta	Sordariomycetes	LZPB01000161	8808-10147	8857-9011		
Ceratocystis adiposa	Sordariomycetes	LXGU01000097	74246-75891	74265-74712		
Ceratocystis albifundus	Sordariomycetes	JSSU01001128	c2814-1234	c2795-2233		
Ceratocystis cacaofunesta	Sordariomycetes	PEJQ01000031	c182757-181273	c182738-182224		
Ceratocystis eucalypticola	Sordariomycetes	LJOA01000096	c115678-114194	c115659-115145		
Ceratocystis fagacearum	Sordariomycetes	MKGJ01000142	38200-39899	38219-38838		
Ceratocystis fimbriata	Sordariomycetes	APWK03000118	c166413-164929	c166394-165880		
Ceratocystis harringtonii	Sordariomycetes	MKGM01000318	c25710-24237	c25691-25172		
Ceratocystis manginecans	Sordariomycetes	JJRZ01000161	c63808-62324	c63789-63275		
Ceratocystis platani	Sordariomycetes	LBBL01000161	20356-21840	20375-20889		
Cercospora berteroae	Dothideomycetes	PNEN01000556	167377-169005	167489-167844		
Cercospora beticola	Dothideomycetes	LKMD01000099	299704-301294	299816-300133		
Cercospora canescens	Dothideomycetes	ANSM01002468	3144-4704	3265-3579		
Cercospora cf. sigesbeckiae	Dothideomycetes	NKQR01000059	194676-196292	194788-195131		
Cercospora nicotianae	Dothideomycetes	POSS01000014	437445-439067	437557-437906		
Cercospora sojina	Dothideomycetes	AHPQ01000070	c22809-21204	c22697-22364		
Cercospora zeina	Dothideomycetes	MVDW01000104	c23907-22317	c23777-23433		
Cetradonia linearis	Lecanoromycetes	QKZZ01001122	c20727-19364	c20594-20481		
Chaetomium cochliodes	Sordariomycetes	LSBY01000262	c15677-14282	c15622-15474		
Chaetomium globosum	Sordariomycetes	AAFU01000787	49882-51227	49937-50080		
Chaetomium thermophilum	Sordariomycetes	ADUW01000027	2073290-2074842	2073321-2073588		
Chalaropsis thielavioides	Sordariomycetes	BCGU01000003	3030451-3031933	3030470-3030971		
Chlorociboria aeruginascens	Leotiomycetes	NCSK02000022	c102251-100971	c102184-102082		
Choiromyces venosus	Pezizomycetes	PZQU01001530	64118-65593	64173-64331		
Chrysoporthe austroafricana	Sordariomycetes	JYIP01002718	c5068-3592	c5019-4817		
Chrysoporthe cubensis	Sordariomycetes	LJCY01000066	1534-2918	1583-1785		
		LJDD01002031	1-105			
Chrysosporium queenslandicum	Eurotiomycetes	LJPI01001409	142523-144036	142654-142772		142563-142882
Ciborinia camelliae	Leotiomycetes	LGKQ01000946	c1922-642	c1834-1702		
Cladonia macilenta	Lecanoromycetes	AUPP01000742	39768-41130	39901-40013		
Cladonia metacorallifera	Lecanoromycetes	AXCT02000485	101285-102647	101418-101530		
Cladonia uncialis	Lecanoromycetes	NAPT01000240	c25342-23974	c25209-25097		
Cladophialophora bantiana	Eurotiomycetes	JYBT01000035	c168879-167427	c168746-168628		
Cladophialophora carrionii_1	Eurotiomycetes	AOFF01000016	c268734-267223	c268613-268504		
Cladophialophora carrionii_2	Eurotiomycetes	LGRB01000014	1059594-1061106	1059715-1059825		
Cladophialophora immunda	Eurotiomycetes	JSEJ01000122	c4752-3301	c4619-4495		
Cladophialophora psammophila	Eurotiomycetes	AMGX01000015	c76813-75354	c76680-76561		
Cladophialophora yegresii	Eurotiomycetes	AMGW01000005	c3468360-3466831	c3468239-3468127		
Cladosporium cladosporioides IMV 00236	(Eurotiomycetes)	MSJH02000794	7010-8453	7062-7201		
Cladosporium cladosporioides TYU	Dothideomycetes	NOXB01000011	289427-291162	289515-289840		
Cladosporium phlei	Dothideomycetes	QZFA01000157	37714-39344	37814-38119		
Cladosporium sp. SL-16	Dothideomycetes	PEGC01000055	c283079-281325	c282988-282654		
Cladosporium sphaerospermum	Dothideomycetes	MSJI02000839	32884-34681	32981-33415		
Cladosporium sphaerospermum	Dothideomycetes	AIIA02000121	c30669-29060	c30578-30255		
Claviceps fusiformis	Sordariomycetes	AFRA01000097	c50027-48500	c4997-49815		
Claviceps paspali	Sordariomycetes	AFRC01000364	c9278-7741	c9226-9057		
Claviceps purpurea	Sordariomycetes	CAGA01000010	c391161-389588	c391109-390941		
Clohesyomyces aquaticus	Dothideomycetes	MCFA01000266	c11059-9510	c10902-10743	c10682-10567	
Clonostachys rosea	Sordariomycetes	JYFM02000288	910594-911910	910652-910788		
Clonostachys rosea	Sordariomycetes	JYFM02000288	910594-911910	910652-910788		
Coccidioides immitis	Eurotiomycetes	AAEC03000001	c795281-793728	c795133-795003		c795244-794891
Coccidioides posadasii	Eurotiomycetes	ACFW01000012	c233288-231730	c233138-233005		c233251-232893
Coleophoma crateriformis	Leotiomycetes	PDLN01000002	428267-429568	428385-428497		
Coleophoma cylindrospora	Leotiomycetes	PDLM01000004	445671-446972	445789-445901		
Colletotrichum acutatum	Sordariomycetes	LUXP01000016	2223198-2224584	2223274-2223424		
Colletotrichum chlorophyti	Sordariomycetes	MPGH01000008	1150740-1152121	1150816-1150977		
Colletotrichum coccodes	Sordariomycetes	LECP01000087	c458404-457025	c458328-458176		
Colletotrichum falcatum	Sordariomycetes	LPVI01004095	12969-14330	13045-13175		
Colletotrichum fioriniae	Sordariomycetes	JARH01000666	c68135-66748	c68059-67909		
Colletotrichum fructicola	Sordariomycetes	MVNS01000004	c140415-139051	c140339-140177		
Colletotrichum gloeosporioides	Sordariomycetes	ANPB01002759	c15515-14151	c15439-15277		
Colletotrichum godetiae	Sordariomycetes	LZRM01000945	15458-16842	15534-15683		
Colletotrichum graminicola	Sordariomycetes	ACOD01000135	c26960-25578	c26884-26730		
Colletotrichum higginsianum	Sordariomycetes	LTAN01000005	c3120649-3119257	c3120573-3120414		
Colletotrichum incanum	Sordariomycetes	LFIW01001981	c123723-122338	c123647-123494		
Colletotrichum lentis	Sordariomycetes	NWBT01000045	2409375-2410767	2409451-2409609		
Colletotrichum lindemuthianum	Sordariomycetes	MASO02000362	c33439-32042	c33363-33186		
Colletotrichum musae	Sordariomycetes	NWMS01001108	c4973-3606	c4897-4735		
Colletotrichum nymphaeae	Sordariomycetes	JEMN01001318	42651-44037	42727-42877		
Colletotrichum orbiculare	Sordariomycetes	AMCV01005706	28900-30294	28976-29156		
Colletotrichum orchidophilum	Sordariomycetes	MJBS01000079	74506-75885	74582-74729		
Colletotrichum salicis	Sordariomycetes	JFFI01002634	c2553-1171	c2477-2329		
Colletotrichum sansevieriae	Sordariomycetes	NJHP01000795	13709-15087	13785-13944		
Colletotrichum simmondsii	Sordariomycetes	JFBX01000170	58855-60241	58931-59081		
Colletotrichum sp. JS-367	Sordariomycetes	QCWU01000004	c4440460-4439101	c4440384-4440227		
Colletotrichum sublineola	Sordariomycetes	JMSE01000926	75307-76686	75383-75537		
Colletotrichum tofieldiae	Sordariomycetes	LFHS01000324	c186463-185079	c186387-186234		
Colletotrichum truncatum	Sordariomycetes	NBAU02000002	1860414-1861802	1860490-1860652		
Coniella lustricola	Sordariomycetes	NSBW01000214	1391-2999	1440-1646		
Coniochaeta hoffmannii	Sordariomycetes	NXFW01000110	c54787-53353	c54726-54545		
Coniochaeta ligniaria	Sordariomycetes	MNPN01000031	c554608-553214	c554547-554380		
Coniochaeta pulveracea	Sordariomycetes	QVQW01000008	c330354-328960	c330260-330109		
Coniosporium apollinis	Dothideomycetes	AJKL01000123	79981-81652	80114-80254	80327-80431	
Cordyceps brongniartii	Sordariomycetes	AZHA01000008	c241972-240683	c241911-241783		
Cordyceps cicadae	Sordariomycetes	AEIW01001579	c47540-46239	c47479-47338		
Cordyceps confragosa	Sordariomycetes	AZHF01000001	3167712-3169000	3167773-3167904		
Cordyceps farinosa	Sordariomycetes	LUFE01000004	1124803-1126077	1124864-1124996		
Cordyceps militaris	Sordariomycetes	AEVU01000218	c235572-234327	c235511-235396		
Cordyceps pruinosa	Sordariomycetes	LUFD01002986	18258-19559	18319-18452		
Cordyceps sp. RAO-2017	Sordariomycetes	NJEV01000477	c2278-907	c2211-2056		
Cordyceps tenuipes	Sordariomycetes	LUFF01000188	c1201233-1199966	c1201172-1201046		
Corinectria fuckeliana	Sordariomycetes	QGQC01000411	c25493-24102	c25435-25276		
Corynespora cassiicola	Dothideomycetes	JAQF01000084	20-1580	210-328	401-514	
Cryomyces antarcticus	Dothideomycetes	AYQD01001440	1560-3475			
Cryphonectria radicalis	Sordariomycetes	LATA01000163	c19144-17653	c19095-18863		
Curvularia geniculata	Dothideomycetes	PQMW01000126	c47561-46098	c47389-47268	c47195-47083	
Curvularia geniculata (paralogue)	Dothideomycetes	PQMW01000007	222577-224176			
Curvularia lunata	Dothideomycetes	JFHG01000898	213-1679	385-506	579-691	
Curvularia lunata (paralogue)	Dothideomycetes	JFHG01001504	c23067-21462			
Curvularia papendorfii	Dothideomycetes	JXCC01000036	c132861-131399	c132690-132569	c132496-132384	
Curvularia papendorfii (paralogue)	Dothideomycetes	JXCC01000260	c30843-29241			
Curvularia sp. IFB-Z10	Dothideomycetes	JPSZ01000006	c638831-637371	c638653-638535	c638462-638350	
Curvularia sp. IFB-Z10 (paralogue)	Dothideomycetes	JPSZ01000007	787754-789431			
Cyphellophora europaea	Eurotiomycetes	AOBU01000020	c456100-454768	c455982-455885		
Cytospora leucostoma	Sordariomycetes	LKEB01000004	c404728-403422			
Dactylellina haptotyla	Orbiliomycetes	AQGS01000291	c52048-50770	c51939-51792		
Dactylonectria macrodidyma	Sordariomycetes	JYGD01000510	c348025-346659	c347967-347809		
Daldinia eschscholzii	Sordariomycetes	AIID01007367	c2081-714	c2026-1850		
Daldinia sp. EC12	Sordariomycetes	MDGZ01000247	200499-201859	200554-200729		
Davidsoniella virescens	Sordariomycetes	LJZU01000015	c35433-33907	c35414-34959		
Diaporthe ampelina	Sordariomycetes	LWAD01000202	6796-8103			
Diaporthe aspalathi	Sordariomycetes	LJJS01001786	118749-120067			
Diaporthe helianthi	Sordariomycetes	MAVT02000038	13601-14912			
Diaporthe longicolla	Sordariomycetes	AYRD02012372	c297663-296381			
Diaporthe longicolla	Sordariomycetes	AYRD02012372	c127577-126295			
Didymobotryum rigidum	Sordariomycetes	BCKI01000001	c3993031-3991694	c3992982-3992820		
Diplocarpon rosae_1	Leotiomycetes	MVNX01000021	c317394-316029	c317288-317155		
Diplocarpon rosae_2	Leotiomycetes	MVNX01000102	188567-189920	188673-188794		
Diplodia corticola	Dothideomycetes	MNUE01000012	574809-576551	575002-575159	575220-575343	
Diplodia sapinea	Dothideomycetes	AXCF01000109	25931-27671	26124-26274	26335-26457	
Diplodia scrobiculata	Dothideomycetes	LAEG01001133	c2856-1116	c2663-2513	c2452-2330	
Diplodia seriata	Dothideomycetes	LAQI01000081	364777-366517	364970-365120	365181-365303	
Dothistroma pini	Dothideomycetes	MMSO01000910	107690-109091	107790-108070		
Dothistroma septosporum	Dothideomycetes	AIEN01000038	39905-41306	40005-40284		
Drechmeria coniospora	Sordariomycetes	LAYC01000001	c2127360-2125965	c2127293-2127146		
Duddingtonia flagrans	Orbiliomycetes	MRVF01000003	c225394-224236	c225336-225151		
Elaphomyces granulatus	Eurotiomycetes	NPHW01003253	18322-19738	18371-18579		
Elsinoe australis	Dothideomycetes	NHZQ01000072	c78557-77135	c78451-78289		
Elsinoe sp. CQ-2017a	Dothideomycetes	NKHZ01000081	671716-673179	671834-671993		
Elsinoe sp. CQ-2017a	Dothideomycetes	NKHZ01000081	671716-673179	671834-672038		
Emergomyces orientalis	Eurotiomycetes	MOWL01000048	570531-572260	570661-570818		570574-570954
Emergomyces pasteuriana	Eurotiomycetes	LGRN01000043	23575-25373	23714-23905		23618-24053
Emmonsia crescens	Eurotiomycetes	LCZI01001228	c127020-125311	c126892-126753		c126977-126607
Emmonsia parva	Eurotiomycetes	LDEV01001769	c32525-30762	c32411-32236		c32482-32084
Emmonsia sp. CAC-2015a	Eurotiomycetes	LGUA01000800	c9089-7226	c8957-8779		c9046-8634
Endocalyx cinctus	(Sordariomycetes)	BCKC01000001	c5696668-5695202	c5696619-5696457		
Endocarpon pusillum	Eurotiomycetes	JFDM01000343	38951-40457	39096-39334		
Endoconidiophora laricicola	Sordariomycetes	LXGT01000131	c76356-74749	c76337-75811		
Endoconidiophora polonica	Sordariomycetes	LXKZ01000381	1451-3037	1470-1978		
Epichloe amarillans	Sordariomycetes	AFRF01000178	14866-16272	14918-15071		
Epichloe aotearoae	Sordariomycetes	JFGX01000060	c84813-83412	c84761-84612		
Epichloe baconii	Sordariomycetes	JFGY01000031	c19651-18246	c19599-19446		
Epichloe brachyelytri	Sordariomycetes	AFRB01000075	c13526-12117	c13474-13318		
Epichloe bromicola	Sordariomycetes	LBNH01001460	14100-15505	14152-14305		
Epichloe elymi	Sordariomycetes	AMDJ01000067	59935-61347	59987-60140		
Epichloe festucae	Sordariomycetes	AFRX02000278	c43595-42187	c43543-43387		
Epichloe gansuensis	Sordariomycetes	AFRE01000005	2955-4362	3007-3163		
Epichloe glyceriae	Sordariomycetes	AFRG01000201	c33407-31999	c33355-33199		
Epichloe sp. AL9924	Sordariomycetes	JFGW01000011	111804-113213	111856-112012		
Epichloe sylvatica	Sordariomycetes	LCTT01000047	7207-8605	7259-7405		
Epichloe typhina	Sordariomycetes	AMDI01000377	21886-23286	21938-22084		
Epichloe uncinata	Sordariomycetes	LELE01001736	c2841-1443	c2789-2643		
Epicoccum nigrum	Dothideomycetes	NCTX01000165	c16175-14721	c16030-15910	c15837-15726	
Epicoccum sorghinum	Dothideomycetes	MIEO01000285	6380-7849	6546-6667	6740-6850	
Erysiphe necator	Leotiomycetes	JNUS01000126	9550-10818	9620-9733		
Erysiphe pisi	Leotiomycetes	CACM01011860	c2092-821	c2021-1914		
Erysiphe pulchra	Leotiomycetes	PEDP01000739	c3165-1891	c3095-2983		
Escovopsis sp. AC	Sordariomycetes	NQYS01000002	c3428766-3427078	c3428717-3428084		
Escovopsis sp. Ae720	Sordariomycetes	NQYQ01000001	c1976538-1974965	c1976489-1975988		
Escovopsis sp. TC	Sordariomycetes	NIGD01000003	c4782505-4780835	c4782456-4781907		
Escovopsis weberi	Sordariomycetes	LGSR01000022	c2629956-2628276	c2629907-2629282		
Esteya vermicola	Sordariomycetes	PCDM01000001	2489655-2490752	2489695-2489885		
Eurotiomycetes sp. A	Eurotiomycetes	PCFH01000007	421891-423435	422030-422159		
Eurotiomycetes sp. B	Eurotiomycetes	PCFI01000034	68442-70042	68629-68755		
Eutypa lata	Sordariomycetes	AORF01001716	c33907-32542	c33849-33701		
Evernia prunastri	Lecanoromycetes	NKYR01000082	c133108-131471	c132987-132873		
Exophiala alcalophila	Eurotiomycetes	BCHY01000003	c1938661-1937171	c1938537-1938424		
Exophiala aquamarina	Eurotiomycetes	AMGV01000018	438939-440218	439024-439136		
Exophiala calicioides	Eurotiomycetes	BCHZ01000003	c4298392-4296968	c4298271-4298158		
Exophiala dermatitidis	Eurotiomycetes	AFPA01000163	c80827-79248	c80703-80588		
Exophiala mesophila	Eurotiomycetes	JSEI01000096	c93973-92666	c93849-93742		
Exophiala oligosperma	Eurotiomycetes	JYCA01000011	1122188-1123637	1122315-1122425		
Exophiala sideris	Eurotiomycetes	JYBR01000005	156105-157786	156283-156393		
Exophiala spinifera	Eurotiomycetes	JYBY01000001	116940-118408	117049-117182		
Exophiala xenobiotica	Eurotiomycetes	JTCI01000326	c30572-29140	c30430-30322		
Exutisphaerella laricina	Dothideomycetes	AWYE02000919	c197864-196376	c197740-197430		
Falciphora oryzae	Sordariomycetes	JNVV01000003	2096700-2098193	2096752-2096970		
Fonsecaea erecta	Eurotiomycetes	LVYI01000009	c1390452-1389001	c1390319-1390196		
Fonsecaea monophora	Eurotiomycetes	LVKK01000036	c271935-270468	c271793-271671		
Fonsecaea multimorphosa	Eurotiomycetes	JYBV01000026	80349-81796	80482-80607		
Fonsecaea nubica	Eurotiomycetes	LVCJ01000034	116167-117625	116300-116422		
Fonsecaea pedrosoi	Eurotiomycetes	JYBS01000043	c919599-918140	c919466-919344		
Fragosphaeria purpurea	Sordariomycetes	PCDL01000021	4453971-4455475	4454014-4454201		
Fungal sp. EF0021	Leotiomycetes	AIET01000648	c61092-59794	c61001-60890		
Fusarium acuminatum	Sordariomycetes	CBMG010001017	2971-4368	3029-3192		
Fusarium agapanthi	Sordariomycetes	LTDI01000153	189610-191008	189668-189835		
Fusarium algeriense	Sordariomycetes	PVPY01000492	76931-78323	76989-77149		
Fusarium asiaticum	Sordariomycetes	LHTY01000095	209318-210725	209376-209535		
Fusarium avenaceum	Sordariomycetes	JPYM01000005	247326-248727	247384-247547		
Fusarium azukicola	Sordariomycetes	MAEG01009134	c2169-765	c2111-1934		
Fusarium beomiforme	Sordariomycetes	PVQB01000114	147592-148987	147650-147816		
Fusarium brasiliense	Sordariomycetes	MAEC01003207	2442-3837	2500-2677		
Fusarium burgessii	Sordariomycetes	PVQA01000276	76638-78030	76696-76856		
Fusarium circinatum	Sordariomycetes	AYJV02000021	120237-121633	120295-120462		
Fusarium culmorum	Sordariomycetes	CBMH010001477	11532-12939	11590-11749		
Fusarium cuneirostrum	Sordariomycetes	MAEA01003220	2460-3864	2518-2695		
Fusarium equiseti	Sordariomycetes	MTPY01000098	c354314-352909	354256-354097		
Fusarium euwallaceae	Sordariomycetes	NHTE02000009	c2338031-2336668	c2337973-2337804		
Fusarium fracticaudum	Sordariomycetes	PDNT01000002	c1824924-1823528	c1824866-1824699		
Fusarium fujikuroi	Sordariomycetes	ANFV01000123	c37045-35625	c36987-36797		
Fusarium graminearum	Sordariomycetes	AACM02000348	c114062-112655	c114004-113845		
Fusarium hostae	Sordariomycetes	NJCQ01001007	c115466-114067	c115408-115240		
Fusarium langsethiae	Sordariomycetes	JXCE01000041	83695-85102	83753-83912		
Fusarium longipes	Sordariomycetes	PXOG01000135	81105-82501	81163-81321		
Fusarium mangiferae	Sordariomycetes	FCQH01000010	c1821896-1820489	c1821838-1821661		
Fusarium meridionale	Sordariomycetes	LHUA01000479	47982-49389	48040-48199		
Fusarium nygamai	Sordariomycetes	LBNR01000005	c290118-288720	c290060-289893		
Fusarium oxysporum	Sordariomycetes	MALV01000136	c64133-62734	c64075-63907		
Fusarium phaseoli	Sordariomycetes	MAEB01000902	c13536-12132	c13478-13301		
Fusarium pininemorale	Sordariomycetes	NFZR01000002	3163428-3164824	3163486-3163653		
Fusarium poae	Sordariomycetes	LYXU01000004	5444369-5445775	5444427-5444584		
Fusarium praegraminearum	Sordariomycetes	LXHY01000019	152289-153691	152347-152506		
Fusarium proliferatum	Sordariomycetes	FJOF01000002	3024004-3025409	3024062-3024237		
Fusarium pseudograminearum	Sordariomycetes	AFNW01000016	103175-104582	103233-103392		
Fusarium sambucinum	Sordariomycetes	LSRD01000018	189935-191344	189993-190153		
Fusarium solani	Sordariomycetes	NGZQ01000004	c2248736-2247328	c2248678-2248509		
Fusarium sp. FIESC_12	Sordariomycetes	PXXK01000224	c72796-71391	c72738-72579		
Fusarium sp. FIESC_28	Sordariomycetes	QRXC01000136	c549506-548109	c549448-549289		
Fusarium sp. FSSC_6	Sordariomycetes	LNBZ01000030	c2149052-2147644	c2148994-2148825		
Fusarium sp. JS1030	Sordariomycetes	JMIW01000091	c672154-670736	c672096-671920		
Fusarium sp. JS626	Sordariomycetes	JWIV01000059	664507-665908	664565-664728		
Fusarium sp. Na10	Sordariomycetes	NJCS01000224	c65834-64438	c65776-65609		
Fusarium sporotrichioides	Sordariomycetes	PXOF01000109	c63909-62502	c63851-63692		
Fusarium temperatum	Sordariomycetes	LJGR01000002	3043895-3045291	3043953-3044120		
Fusarium tricinctum	Sordariomycetes	PTXX01000001	101865-103266	101923-102086		
Fusarium tucumaniae	Sordariomycetes	MAED01002833	c2538-1135	c2480-2304		
Fusarium udum	Sordariomycetes	NIFK01000002	3311708-3313116	3311766-3311938		
Fusarium verticillioides	Sordariomycetes	AAIM02000077	c492297-490898	c492239-492071		
Fusarium virguliforme	Sordariomycetes	AEYB01000498	6283-7687	6341-6518		
Gaeumannomyces sp. JS-464	Sordariomycetes	NGZR01000001	7232279-7233779	7232331-7232547		
Gaeumannomyces tritici	Sordariomycetes	ADBI01000276	c55832-54342	c55780-55552		
Gamarada debralockiae	Leotiomycetes	NXFV01000304	23423-24733	23517-23635		
Geosmithia flava	Sordariomycetes	FYEV01001791	308181-309758	308224-308419		
Geosmithia morbida	Sordariomycetes	FNXU01000001	c1726048-1724529	c1726005-1725825		
Geosmithia putterillii	Sordariomycetes	FYEU01000249	c34717-33275	c34674-34449		
Glarea lozoyensis	Leotiomycetes	AGUE01000016	340386-341696	340504-340617		
Gliomastix tumulicola	Sordariomycetes	BCHX01000003	c1675440-1674107	c1675373-1675226		
Glonium stellatum	Dothideomycetes	LKAO01002559	11053-12602	11186-11302	11375-11488	
Golovinomyces cichoracearum	Leotiomycetes	MCBQ01020451	c3376-2081	c3291-3176		
Graphilbum fragrans	Sordariomycetes	LLKO01000019	c410919-409656	c410870-410565		
Grosmannia clavigera	Sordariomycetes	ACXQ02000023	986165-987461	986217-986484		
Grosmannia penicillata	Sordariomycetes	PCDK01000035	979201-980486	979244-979492		
Gyalolechia flavorubescens	Lecanoromycetes	AUPK01000073	c55038-53547	c54896-54787	c54726-54605	
Hawksworthiomyces lignivorus	Sordariomycetes	NTMA01000003	c985094-983766	c985051-984773		
Helicocarpus griseus	Eurotiomycetes	PDNB01000045	83555-85135	83670-83809		83598-83951
Helminthosporium solani	Dothideomycetes	AWWW01000087	40401-42000	40507-40629	40702-40991	
Helotiales sp. F229	Leotiomycetes	MXAW01000005	1576516-1577839	1576616-1576732		
Helotiales sp. T6G9	Leotiomycetes	NXFV01000304	23423-24733	23517-23635		
Herpotrichiellaceae sp.	Eurotiomycetes	AMYF01000073	45908-47334	46023-46123		
Hirsutella minnesotensis	Sordariomycetes	JPUM01000102	c185687-184385	c185623-185492		
Hirsutella thompsonii	Sordariomycetes	APKB01000075	c145291-144000	c145227-145099		
Histoplasma capsulatum	Eurotiomycetes	AAJI01002417	c63906-62216	c63793-63625		c63863-63479
Hortaea werneckii_1	Dothideomycetes	MUNK01000208	5146-6558	5261-5516		
Hortaea werneckii_2	Dothideomycetes	MUNK01000033	c96024-94609	c95909-95651		
Huntiella bhutanensis	Sordariomycetes	MJMS01000081	c66625-65076	c66609-66029		
Huntiella decipiens	Sordariomycetes	NETU01000271	64111-65664	64127-64710		
Huntiella moniliformis	Sordariomycetes	JMSH01000017	141566-143116	141582-142169		
Huntiella omanensis	Sordariomycetes	JSUI01006519	c66302-64747	c66286-65701		
Huntiella savannae	Sordariomycetes	LCZG01000032	74611-76163	74627-75210		
Hymenoscyphus fraxineus	Leotiomycetes	FXXO01000789	16805-18103	16896-17013		
Hymenoscyphus fructigenus	Leotiomycetes	LKUV01000062	c45915-44581	c45812-45690		
Hymenoscyphus infarciens	Leotiomycetes	LLCB01000319	43007-44347	43110-43233		
Hymenoscyphus laetus	(Dothideomycetes)	LLCA01000004	331593-332940	331669-331790	331863-331973	
Hymenoscyphus repandus	Leotiomycetes	LLCE01000067	c99472-98175	c99372-99260		
Hymenoscyphus salicellus	Leotiomycetes	LLCD01004139	c2816-1490	c2722-2599		
Hymenoscyphus scutula	Leotiomycetes	LKTO01000029	c49368-48041	c49274-49151		
Hypocrella siamensis	Sordariomycetes	JMQE01001689	50057-51433	50109-50238		
Hypoxylon pulicicidum	Sordariomycetes	PDUJ01000002	c1915720-1914403	c1915659-1915515		
Hypoxylon sp. CI-4A	Sordariomycetes	MDGY01000079	c45047-43719	c44986-44846		
Hypoxylon sp. CO27-5	Sordariomycetes	MDCL01000052	193440-194763	193501-193651		
Hypoxylon sp. E7406B	Sordariomycetes	JYCQ01000010	c485240-483917	c485179-485032		
Hypoxylon sp. EC38	Sordariomycetes	MDCK01000025	c97218-95882	c97157-96998		
Hysterium pulicare	Dothideomycetes	AJFK01000319	c48668-47137	c48532-48414	c48341-48227	
Ilyonectria destructans	Sordariomycetes	MPHF01000041	163186-164546	163244-163408		
Ilyonectria mors-panacis	Sordariomycetes	PPHJ01000015	894298-895724	894356-894521		
Isaria farinosa	Sordariomycetes	JMNC01000803	34958-36270	35019-35157		
Isaria fumosorosea	Sordariomycetes	AZHB01000013	328675-329949	328736-328874		
Juglanconis juglandina	Sordariomycetes	MUZJ01011366	1656-3099	1705-1893		
Juglanconis oblonga	Sordariomycetes	MUZH01003637	c2312-869	c2263-2075		
Juglanconis sp. DMW523	Sordariomycetes	MUZK01002086	c2251-809	c2202-2014		
Knoxdaviesia capensis	Sordariomycetes	LNGK01000001	1006291-1008385	1006319-1007043		
Knoxdaviesia proteae	Sordariomycetes	LNGL01000029	c96499-94412	c96471-95737		
Knufia petricola	Eurotiomycetes	NMQP01000070	c43820-42261	c43666-43561		
Kretzschmaria deusta	Sordariomycetes	MLHU03000530	c9287-7734	c9232-8887		
Lasallia hispanica	Lecanoromycetes	PKMA01000328	c39519-37943	c39374-39262	c39189-39080	
Lasallia pustulata	Lecanoromycetes	FWEW01000076	c13640-12064	c13495-13383	c13310-13201	
Lasiodiplodia theobromae	Dothideomycetes	MDYX01000019	532420-534146	532604-532760	532821-532945	
Lecanicillium fungicola	Sordariomycetes	FWCC01000054	26652-27955	26713-26848		
Lecanicillium psalliotae	Sordariomycetes	PHFE01000107	c1755249-1753980	c1755188-1755056		
Lecanicillium sp. LEC01	Sordariomycetes	NIWZ02000007	44902-46329	44969-45125		
Lecanicillium sp. MT-2017a	Sordariomycetes	PXYM01000380	313002-314447	313069-313263		
Lecanosticta acicola	Dothideomycetes	AWYC02001171	c2322-749	c2201-1776		
Lepidopterella palustris	Dothideomycetes	LKAR01000068	c93103-91560	c92970-92852	c92779-92665	
Leptographium lundbergii	Sordariomycetes	LDEF01000009	c113170-111867	c113118-112839		
Leptographium penicillatum	Sordariomycetes	MLJV01000079	54462-55747	54505-54753		
Leptographium procerum	Sordariomycetes	JRUC01000472	c18668-17390	c18616-18341		
Leptosphaeria biglobosa	Dothideomycetes	FO905648	c371766-370261	c371570-371449	c371376-371275	
Leptosphaeria maculans	Dothideomycetes	FP929138	1290692-1292211	1290888-1291015	1291088-1291191	
Leptoxyphium fumago	Dothideomycetes	LSHF01000067	c86865-85430	c86777-86446		
Lipomyces arxii	Saccharomycotina	PPJT01000025	c150108-148881			
Lipomyces doorenjongii	Saccharomycotina	PPJU01000019	c47680-46837			
Lipomyces japonicus	Saccharomycotina	PPJV01000031	c133557-132264			
Lipomyces kononenkoae	Saccharomycotina	PPJW01000022	109847-111134			
Lipomyces lipofer	Saccharomycotina	PPJX01000048	c101794-100457			
Lipomyces mesembrius	Saccharomycotina	PPJS01000050	91266-92498			
Lipomyces oligophaga	Saccharomycotina	PPJR01000005	931312-932229			
Lipomyces starkeyi	Saccharomycotina	LSGR01000110	c256819-255558			
Lipomyces suomiensis	Saccharomycotina	PPJQ01000021	223901-224934			
Lobosporangium transversale_1	Mortierellomycotina	MCFF01000001	c542760-541602			
Lobosporangium transversale_2	Mortierellomycotina	MCFF01000014	c82047-80272			
Lomentospora prolificans	Sordariomycetes	NLAX01000010	c4457176-4455673	c4457112-4456916		
Macrophomina phaseolina	Dothideomycetes	AHHD01000253	10792-12515	10964-11128	11189-11312	
Madurella mycetomatis	Sordariomycetes	LCTW02000021	31617-32936	31669-31826		
Magnaporthales sp. P1609	Sordariomycetes	PELF01000003	2384106-2385545	2384164-2384334		
Magnaporthe grisea	Sordariomycetes	LOEM01000323	91650-93105	91708-91872		
Magnaporthe oryzae	Sordariomycetes	AACU03000146	c230533-229083	c230475-230314		
Magnaporthiopsis incrustans	Sordariomycetes	PYSR01000126	c74227-72686	c74175-73933		
Magnaporthiopsis poae	Sordariomycetes	ADBL01000200	9502-11021	9554-9769		
Magnaporthiopsis rhizophila	Sordariomycetes	PYSS01000003	c283237-281710	c283185-282962		
Malbranchea cinnamomea CBS 343.55	Eurotiomycetes	Malci1_Scaffold0039	159564-161280	159694-159882		159596-160059
Malbranchea cinnamomea FCH 10.5	Eurotiomycetes	FQSS02000663	c52626-50929	c52497-52315		c52595-52138
Marssonina brunnea	Leotiomycetes	AFXC01000366	97214-98551	97326-97442		
Marssonina coronariae	Leotiomycetes	MZNU01000099	164504-165857	164610-164731		
Meliniomyces bicolor	Leotiomycetes	LXPI01000273	c800015-798712	c799921-799807		
Meliniomyces variabilis	Leotiomycetes	LXPR01000014	c1049990-1048711	c1049896-1049782		
Metarhizium acridum	Sordariomycetes	ADNI01000873	9588-10977	9640-9787		
Metarhizium album	Sordariomycetes	AZHE01000008	c490446-489051	c490394-490246		
Metarhizium anisopliae	Sordariomycetes	AZNF01000001	1682685-1684063	1682737-1682881		
Metarhizium brunneum	Sordariomycetes	AZNG01000001	1060574-1061953	1060626-1060770		
Metarhizium guizhouense	Sordariomycetes	AZNH01000002	c1495435-1494048	c1495383-1495239		
Metarhizium majus	Sordariomycetes	AZNE01000043	c52365-50978	c52313-52169		
Metarhizium rileyi	Sordariomycetes	AZHC01000005	c752825-751415	c752773-752632		
Metarhizium robertsii	Sordariomycetes	ADNJ02000002	4833382-4834760	4833434-4833578		
Microcyclospora pomicola	Dothideomycetes	NNNY01000001	2304688-2306206	2304749-2304997		
Microcyclospora tardicrescens	Dothideomycetes	PXOE01000104	166536-168048	166597-166840		
Microcyclosporella mali	Dothideomycetes	NNNU01000001	c958832-957243	c958741-958274		
Microdochium bolleyi	Sordariomycetes	LSSP01000008	235547-236915	235599-235806		
Microsporum canis	Eurotiomycetes	ABVF01000235	c63220-61467	c63064-62905		c63183-62776
Moelleriella libera	Sordariomycetes	AZGY01000002	c1227586-1226245	c1227534-1227397		
Monascus ruber	Eurotiomycetes	FXMQ01000137	c69126-67649	c69098-68866		
Monilinia aucupariae	Leotiomycetes	NGKF01000100	c3341-2040	c3253-3116		
Monilinia fructicola	Leotiomycetes	PDME01001380	1172-2453	1260-1384		
Monilinia fructigena	Leotiomycetes	PDMD01000655	1520-2799	1608-1729		
Monilinia laxa	Leotiomycetes	PDMC01000166	c67258-65982	c67170-67050		
Monilinia polystroma	Leotiomycetes	PDUE01000181	c91422-90142	c91334-91212		
Morchella conica	Pezizomycetes	PZQV01000936	32878-34294	32945-33070		
Morchella eximia	Pezizomycetes	QMFK01001552	c8114-6703	c8047-7926		
Morchella importuna	Pezizomycetes	QOKS01000022	c293102-291686	c293035-292910		
Morchella septimelata	Pezizomycetes	PYSJ01000233	42681-44092	42748-42869		
Mortierella alpina ATCC 32222_1	Mortierellomycotina	ADAG01000981	106500-107637			
Mortierella alpina ATCC 32222_2	Mortierellomycotina	ADAG01000948	c48082-45993			
Mortierella alpina B6842_1	Mortierellomycotina	AZCI01000995	c294539-293387			
Mortierella alpina B6842_2	Mortierellomycotina	AZCI01001087	c78382-76309			
Mortierella elongata_1	Mortierellomycotina	LYLZ01000347	46217-47647			
Mortierella elongata_2	Mortierellomycotina	LYLZ01000294	c95098-92509			
Mortierella verticillata	Mortierellomycotina	AEVJ01000267	c23983-21358			
Myceliophthora thermophila	Sordariomycetes	CP003007	1202528-1203894	1202583-1202727		
Mycosphaerella arachidis	Dothideomycetes	LIHB01000120	42128-43593	42240-42572		
Mycosphaerella eumusae	Dothideomycetes	LFZN01000048	c110668-109230	c110556-110277		
Mycosphaerella populi	(Eurotiomycetes)	MWPW01000141	c51102-49592	c51062-50782		
Mycosphaerella sp. PB-2012b Mex 2-1-2	Dothideomycetes	MWSN01002427	17506-19205	17660-17997		
Mycosphaerella sp. Ston1	Dothideomycetes	AWYF02000354	5744-7406	5871-6210		
Mycosphaerelloides madeirae	Dothideomycetes	NHNV01000097	13325-14773	13425-13753		
Myriosclerotinia curreyana	Leotiomycetes	NGKJ01000093	52258-53578	52346-52482		
Myriosclerotinia duriaeana	Leotiomycetes	NGKI01000012	916894-918166	916982-917110		
Myriosclerotinia scirpicola	Leotiomycetes	NGKG01000031	c103782-102509	c103694-103567		
Nakataea oryzae	Sordariomycetes	PYSQ01000036	c87093-85613	c87038-86805		
Nannizzia gypsea	Eurotiomycetes	ABQE01000105	123034-124846	123221-123375		123074-123500
Nectria haematococca	Sordariomycetes	ACJF01000015	c2192201-2190792	c2192143-2191974		
Nectria sp. B-13	Sordariomycetes	NOKP01000002	c2957021-2955631	c2956963-2956804		
Neofusicoccum parvum	Dothideomycetes	AORE01000275	52496-54227	52680-52843	52904-53026	
Neolecta irregularis	Taphrinomycotina	LXFE01000241	c16428-15283			
Neonectria ditissima	Sordariomycetes	LDPK01000026	c111741-110351	c111683-111527		
Neonectria hederae	Sordariomycetes	QGQB01000175	c331385-329992	c331327-331169		
Neonectria punicea	Sordariomycetes	QGQA01000326	2220-3609	2278-2436		
Neosartorya udagawae	Eurotiomycetes	BBXM01000130	25084-26512	25136-25260		
Neoscytalidium dimidiatum	Dothideomycetes	FLVB01001070	20871-22569	21040-21197	21258-21382	
Neurospora africana	Sordariomycetes	CAPO02000081	13712-15058	13764-13941		
Neurospora crassa	Sordariomycetes	AABX03000218	172762-174077	172814-172992		
Neurospora pannonica	Sordariomycetes	CAPQ020002778	523-1865	575-750		
Neurospora sublineolata	Sordariomycetes	CAPP020008295	c1937-615	c1885-1708		
Neurospora tetrasperma	Sordariomycetes	AFBT01000097	53683-54998	53735-53913		
Nigrograna mackinnonii	Dothideomycetes	JGVQ01000101	146341-147937	146480-146597	146670-146785	
Nothophaeocryptopus gaeumannii	Dothideomycetes	MWSF01000668	52066-53544	52169-52489		
Ochroconis constricta	Dothideomycetes	AZYM01000068	c80849-79300	c80722-80509	c80436-80312	
Oidiodendron maius	Leotiomycetes	JMDP01000016	269133-270430	269242-269363		
Oidium neolycopersici	Leotiomycetes	MCFK01001696	3476-4749	3546-3658		
Onygena corvina	Eurotiomycetes	JWPT01000019	223396-225093	223558-223678		223445-223806
Ophidiomyces ophiodiicola	Eurotiomycetes	MWKM01000008	1205057-1206338			
Ophioceras dolichostomum	Sordariomycetes	PYST01000004	c2710-1104	c2664-2469		
Ophiocordyceps australis	Sordariomycetes	NJET01000129	c27756-26355	c27689-27495		
Ophiocordyceps camponoti-rufipedis	Sordariomycetes	NJES01000037	c24541-23161	c24474-24335		
Ophiocordyceps polyrhachis-furcata	Sordariomycetes	LKCN01000014	c890507-889198	c894625-894498		
Ophiocordyceps sinensis	Sordariomycetes	ANOV01006606	11182-12503	11249-11380		
Ophiocordyceps sinensis	Sordariomycetes	ANOV01006606	11182-12503	11249-11380		
Ophiocordyceps sinensis	Sordariomycetes	ANOV01006606	11182-12503	11249-11380		
Ophiocordyceps sp. 'camponoti-leonardi'	Sordariomycetes	PDHP01000013	19597-20929	19664-19807		
Ophiocordyceps sp. 'camponoti-saundersi'	Sordariomycetes	PDHQ01000066	42203-43519	42270-42405		
Ophiocordyceps unilateralis	Sordariomycetes	LAZP02000024	20263-21589	20330-20482		
Ophiognomonia clavigignenti-juglandacearum	Sordariomycetes	MUZL01003477	c28918-27438	c28875-28695		
Ophiostoma ips	Sordariomycetes	NTMB01000036	c122175-121025			
Ophiostoma novo-ulmi	Sordariomycetes	AMZD01000152	887419-888564			
Ophiostoma piceae	Sordariomycetes	AQHS01000287	c63340-62166			
Paecilomyces hepiali	Sordariomycetes	LNDK02000066	3519940-3521214	3520001-3520133		
Pallidocercospora crystallina	Dothideomycetes	QQNG01000167	297596-299070	297699-298019		
Paracoccidioides brasiliensis	Eurotiomycetes	ABHV02000385	c118745-117056	c118617-118435		c118702-118303
Paracoccidioides lutzii	Eurotiomycetes	ABHH02000191	c52498-50809	c52379-52200		c52455-52054
Paramyrothecium roridum	Sordariomycetes	PXOD01000077	32827-34170	32894-33026		
Paraphaeosphaeria sporulosa	Dothideomycetes	LXPO01000033	40221-41801	40381-40381	40589-40819	
Paraphoma sp. B47-9	Dothideomycetes	BCLE01000179	c192535-191059	c192360-192246	c192173-192060	
Parastagonospora avenae f. sp. avenae	Dothideomycetes	QVAA01000280	6837-8285	7012-7137	7207-7318	
Parastagonospora avenaria f. sp. tritici	Dothideomycetes	QVWS01000667	c4396-2950	c4221-4097	c4027-3917	
Parastagonospora nodorum	Dothideomycetes	AAGI01000135	c68244-66797	c68069-67947	c67874-67763	
Passalora fulva	Dothideomycetes	AMRR01002404	c13949-12505	c13837-13527		
Penicilliopsis zonata	Eurotiomycetes	MRBM01000055	110258-111738	110304-110492		
Penicillium antarcticum	Eurotiomycetes	MDYN01000065	11840-13296	11877-12016		
Penicillium arizonense	Eurotiomycetes	LXJU01000021	269648-271093	269685-269824		
Penicillium biforme	Eurotiomycetes	CBXO01000048	119311-120795	119348-119487		
Penicillium brasilianum	Eurotiomycetes	CDHK01000004	c426843-425322	c426803-426645		
Penicillium camemberti	Eurotiomycetes	CBVV01000194	c14300-12816	c14263-14124		
Penicillium capsulatum	Eurotiomycetes	JPLQ01000053	2896805-2898250	2896842-2896980		
Penicillium carneum	Eurotiomycetes	CBXS01001538	c14111-12651	c14074-13943		
Penicillium chrysogenum P2niaD18	Eurotiomycetes	JMSF01000011	5229256-5230716	5229293-5229418		
Penicillium chrysogenum Wisconsin 54-1255	Eurotiomycetes	AM920437	c170239-168779	c170202-170077		
Penicillium citrinum	Eurotiomycetes	LKUP01000269	c58793-57190	c58750-58567		
Penicillium coprophilum	Eurotiomycetes	MDDG01000003	2643232-2644783	2643269-2643409		
Penicillium decumbens	Eurotiomycetes	MDYL01000020	c130149-128712	c130109-129967		
Penicillium digitatum	Eurotiomycetes	AKCU01000320	c61369-59923	c61332-61205		
Penicillium expansum MD-8	Eurotiomycetes	JQFZ01000089	c364792-363317	c364755-364624		
Penicillium expansum T01	Eurotiomycetes	AYHP01000414	c389481-388006	c389444-389313		
Penicillium flavigenum	Eurotiomycetes	MLQL01000023	c194000-192529	c193963-193839		
Penicillium freii	Eurotiomycetes	LLXE01000141	9797-11277	9834-9970		
Penicillium fuscoglaucum	Eurotiomycetes	CBXP01000272	17294-18779	17331-17470		
Penicillium griseofulvum	Eurotiomycetes	LHQR01000014	1063553-1065029	1063590-1063719		
Penicillium italicum	Eurotiomycetes	JMDK01000306	30021-31490	30058-30185		
Penicillium janthinellum (TSA)	Eurotiomycetes	GBSP01005888	c2255-1230			
Penicillium nalgiovense	Eurotiomycetes	CBXQ01000017	c162677-161175	c162640-162509		
Penicillium nordicum	Eurotiomycetes	JNNR01000826	c90342-88848	c90305-90156		
Penicillium occitanis	Eurotiomycetes	NPFK01000234	c26548-25095	c26502-26250		
Penicillium oxalicum	Eurotiomycetes	AGIH01000279	c37802-36143	c37762-37587		
Penicillium paneum	Eurotiomycetes	CBXN01000071	c85322-83855	c85285-85154		
Penicillium paxilli	Eurotiomycetes	AOTG01000475	c211308-209732	c211268-211106		
Penicillium polonicum	Eurotiomycetes	MDYM01000019	316695-318178	316732-316868		
Penicillium raistrickii (TSA)	Eurotiomycetes	GFHR01001422	110-512			
		GFHR01004293	1-653			
Penicillium roqueforti	Eurotiomycetes	CBMR01000354	c14658-13198	c14621-14490		
Penicillium sclerotiorum	Eurotiomycetes	MJCA01000076	c37620-36075	c37580-37378		
Penicillium solitum	Eurotiomycetes	JYNM02000004	4524448-4525931	4524485-4524621		
Penicillium sp. CF05	Eurotiomycetes	PKQL01000214	c26834-25332	c26797-26666		
Penicillium sp. HKF2	Eurotiomycetes	MUXA01000089	1812829-1814289	1812866-1812991		
Penicillium steckii	Eurotiomycetes	MLKD01000018	381751-383342	38179-381984		
Penicillium subrubescens	Eurotiomycetes	MNBE01000758	161029-162519	161069-161226		
Penicillium verrucosum	Eurotiomycetes	LAKW02000446	33534-35029	33571-33721		
Penicillium vulpinum	Eurotiomycetes	MDYP01000024	319281-320783	319318-319454		
Periconia macrospinosa	Dothideomycetes	PCYO01000316	c90294-88683	c90176-90050	c89977-89703	
Periconia macrospinosa (paralogue)	Dothideomycetes	PCYO01002500	1281-2747			
Periglandula ipomoeae	Sordariomycetes	AFRD01000215	c20274-18886	c20222-20075		
Pestalotiopsis fici	Sordariomycetes	ARNU01000005	392051-393301			
Pestalotiopsis sp. JCM 9685	Sordariomycetes	BCGF01000006	c2009558-2008305			
Pezicula radicicola	Leotiomycetes	PDUG01000034	c58937-57557	c58819-58688		
Peziza ericae	Leotiomycetes	LYBP01000173	136441-137721	136535-136650		
Phaeoacremonium minimum	Sordariomycetes	AORD01001178	c52034-50682	c51967-51804		
Phaeomoniella chlamydospora	Eurotiomycetes	LCWF01000002	c98821-97363	c98658-98413		
Phialocephala scopiformis	Leotiomycetes	LKNI01000110	177995-179288	178086-178195		
Phialocephala subalpina	Leotiomycetes	FJOG01000041	c32735-31459	c32665-32554		
Phialophora americana	Eurotiomycetes	JYCC01000048	c1524344-1522828	c1524232-1524120		
Phialophora attae	Eurotiomycetes	LFJN01000012	321545-322931	321681-321778		
Phialophora cf. hyalina	Leotiomycetes	NPIC01000011	25466-26808	25569-25688		
Phialophora verrucosa	Eurotiomycetes	MSED01000002	101325-102851	101446-101558		
Phialosimplex sp. HF37	Eurotiomycetes	RAQS01000060	c41170-39803	c41124-40976		
Phoma herbarum	Dothideomycetes	BCGR01000004	c1231003-1229521	c1230810-1230689	c1230616-1230508	
Phyllosticta capitalensis	Dothideomycetes	LOEO01000873	c10271-8648	c10090-9940	c9879-9765	
Phyllosticta citricarpa	Dothideomycetes	AOTE01002557	3769-5371	3941-4089	4150-4264	
Pleosporales sp. UM 1110	Dothideomycetes	AJMS01006724	2866-3670	3044-3157	3230-3334	
		AJMS01008339	c5771-5109			
Pneumocystis carinii	Taphrinomycotina	LFVZ01000006	c399432-398285			
Pneumocystis jirovecii	Taphrinomycotina	LFWA01000004	236757-237921			
Pneumocystis murina	Taphrinomycotina	AFWA02000002	160375-161520			
Pochonia chlamydosporia	Sordariomycetes	LSBJ02000006	c1621008-1619599	c1620956-1620956		
Podosphaera xanthii (TSA)	Leotiomycetes	GEUO01003046	134-1234			
Podospora anserina	Sordariomycetes	FO904936	c8034981-8033657	c8034920-8034772		
Podospora comata	Sordariomycetes	LR026964	c7966658-7965334	c7966597-7966449		
Polytolypa hystricis	Eurotiomycetes	PDNA01000022	c207429-205864	c207297-207155		c207389-207030
Preussia sp. BSL10	Dothideomycetes	LJJI01000004	c172778-170850	c172705-172344		
Protomyces gravidus	Taphrinomycotina	QXDP01000107	177507-178717			
Protomyces inouyei	Taphrinomycotina	QXDQ01000097	c66143-64850			
Protomyces inundatus	Taphrinomycotina	QXDR01000440	c32233-30979			
Protomyces lactucaedebilis	Taphrinomycotina	MCFI01000019	266180-267474			
Protomyces macrosporus	Taphrinomycotina	QXDT01000037	c29087-27815			
Protomyces pachydermus	Taphrinomycotina	QXDU01000264	115232-116529			
Protomyces sp. C29	Taphrinomycotina	QXMI01000157	663049-664340			
Pseudevernia furfuracea	Lecanoromycetes	NKYQ01000004	c557559-555853	c557438-557324		
Pseudocercospora fijiensis	Dothideomycetes	AIHZ01000262	c748354-746861	c748242-747904		
Pseudocercospora musae	Dothideomycetes	LFZO01000324	c39500-38037	c39388-39084		
Pseudocercospora pini-densiflorae	Dothideomycetes	AWYD02003060	4183-5633	4295-4586		
Pseudogymnoascus destructans	Leotiomycetes	AYKP01003862	c25263-23696	c25169-25054		
Pseudogymnoascus pannorum	Leotiomycetes	AYKR01000619	122835-124387	122932-123056		
Pseudogymnoascus verrucosus	Leotiomycetes	LAJO01000188	c36767-35200	c36685-36554		
Pseudohalonectria lignicola	Sordariomycetes	PYSP01000101	26621-28214	26679-26866		
Pseudomassariella vexata	Sordariomycetes	MCFJ01000008	c592768-591473	c592713-592579		
Pseudonectria foliicola	Sordariomycetes	LMTV01000065	13947-15412	14005-14135		
Pseudopyrenochaeta lycopersici	Dothideomycetes	ASRS01005778	12736-14250	12884-13000	13073-13184	
Pseudopyrenochaeta lycopersici (paralogue)	Dothideomycetes	ASRS01011778	c1852-341			
Purpureocillium lilacinum	Sordariomycetes	LOFA01000006	899914-901328	899981-900123		
Pyrenochaeta sp. DS3sAY3a	Dothideomycetes	LXSZ01000058	300317-301799	300465-300582	300655-300767	
Pyrenochaeta sp. UM 256	Dothideomycetes	AOUM02000007	c398682-397210	c398534-398417	c398344-398231	
Pyrenophora seminiperda	Dothideomycetes	ATLS01000610	116614-118069	116786-116906	116979-117090	
Pyrenophora teres	Dothideomycetes	AEEY01002752	390-1871	568-687	760-871	
Pyrenophora tritici-repentis	Dothideomycetes	AAXI01000108	133539-135008	133717-133836	133909-134020	
Pyricularia pennisetigena	Sordariomycetes	PQBJ01002259	c25850-24399	c25792-25631		
Pyronema omphalodes	Pezizomycetes	CATG01000865	c12242-10933	c12175-12063		
Rachicladosporium antarcticum	Dothideomycetes	NAJO01000009	c922324-920810	c922182-921908		
Rachicladosporium sp. CCFEE 5018	Dothideomycetes	NAEU01001661	c6165-4644	c6023-5749		
Raffaelea albimanens	Sordariomycetes	PCDJ01000001	c3867960-3866372	c3867898-3867542		
Raffaelea ambrosiae	Sordariomycetes	PCDI01000013	1154251-1155747	1154309-1154620		
Raffaelea arxii	Sordariomycetes	PCDH01000003	c4113007-4111741	c4112967-4112770		
Raffaelea lauricola	Sordariomycetes	PCDG01000001	4141083-4142217			
Raffaelea quercivora	Sordariomycetes	BCFZ01000001	3508641-3509944	3508693-3509015		
Raffaelea quercus-mongolicae	Sordariomycetes	NIPS01000037	c739390-738105	c739338-739027		
Raffaelea sp. RL272	Sordariomycetes	PCDF01000003	17739-18945	17803-17973		
Raffaelea sulphurea	Sordariomycetes	PCDD01000024	c88199-86962	c88147-87850		
Ramalina intermedia	Lecanoromycetes	PEKF01000031	c8966-7679	c8905-8792		
Ramalina peruviana	Lecanoromycetes	MSTJ01001185	c2138-851	c2077-1964		
Ramichloridium luteum	Dothideomycetes	MTSC01000003	c395266-393437	c395154-394503		
Ramularia collo-cygni	Dothideomycetes	CZLF01000172	c4445-2920	c4348-3996		
Ramularia endophylla	Dothideomycetes	MWSQ01002958	c20565-19007	c20471-20085		
Rasamsonia emersonii	Eurotiomycetes	LASV01000321	c32332-30925	c32283-32082		
Reticulascus tulasneorum	Sordariomycetes	LSAY01000001	c2046158-2044829	c2046082-2045935		
Rhinocladiella mackenziei	Eurotiomycetes	JYBU01000066	c74261-72825	c74137-74017		
Rhynchosporium agropyri	Leotiomycetes	FJUX01000011	54079-55543	54200-54338		
Rhynchosporium commune	Leotiomycetes	FJUW01000020	527501-528961	527622-527756		
Rhynchosporium secalis	Leotiomycetes	FJVC01000151	8775-10232	8890-9027		
Rhytidhysteron rufulum	Dothideomycetes	AJFL01000430	c43289-41714	c43135-42989	c42916-42801	
Rosellinia necatrix	Sordariomycetes	BBSO02000611	c9204-7513	c9149-8652		
Rutstroemia echinophila	Leotiomycetes	JWJA01004559	2153-3460	2241-2399		
Rutstroemia sp. NJR-2017a	Leotiomycetes	NJPR01000036	c707877-706604	c707789-707660		
Rutstroemia sydowiana	Leotiomycetes	JWJB01011415	c2143-865	c2055-1920		
Saitoella complicata	Taphrinomycotina	AEUO01000027	c34893-33520			
Sarocladium oryzae	Sordariomycetes	LOPT01004497	12525-13851			
Sarocladium strictum	Sordariomycetes	OKRM01000002	c1072554-1071213			
Scedosporium apiospermum	Sordariomycetes	JOWA01000090	c710203-708754	c710160-709799		
Scedosporium aurantiacum	Sordariomycetes	JUDQ01000477	c18164-16716	c18121-17780		
Scedosporium boydii	Sordariomycetes	NJFT01000111	18659-20107	18702-19061		
Scedosporium dehoogii	Sordariomycetes	PGIR01000093	123490-124917	123533-123884		
Sclerotinia borealis	Leotiomycetes	AYSA01000024	c24240-22963	c24152-24026		
Sclerotinia glacialis	Leotiomycetes	NGKH01000005	1516192-1517474	1516280-1516404		
Sclerotinia homoeocarpa	Leotiomycetes	LLKF01001382	239-1524	327-457		
Sclerotinia sclerotiorum	Leotiomycetes	AAGT01000069	24121-25390	24209-24330		
Scytalidium lignicola	Leotiomycetes	NCSJ02000227	c2975-1429	c2863-2670		
Scytalidium sp. 3C	Leotiomycetes	JMRO03000013	356708-358252	356802-356999		
Setosphaeria turcica	Dothideomycetes	AIHT01000056	c25595-24085	c25387-25271	c25198-25085	
Shiraia sp. Slf14	Dothideomycetes	AXZN01000002	80296-81765	80471-80591	80664-80778	
Sordaria macrospora	Sordariomycetes	CABT02000002	c1316091-1314675	c1315982-1315798		
Sphaceloma murrayae	Dothideomycetes	NKHZ01000081	671716-673179	671834-672038		
Sphaerulina musiva	Dothideomycetes	AEFD01000097	c53490-51792	c53336-52994		
Sphaerulina populicola	Dothideomycetes	AIDU01001351	c51300-49597	c51134-50795		
Sporothrix brasiliensis	Sordariomycetes	AWTV01000003	907997-909421	908040-908219		
Sporothrix globosa	Sordariomycetes	LVYW01000002	c955340-953937	c955297-955117		
Sporothrix insectorum	Sordariomycetes	AZHD01000021	c404986-403489	c404943-404762		
Sporothrix pallida	Sordariomycetes	JNEX02000032	127546-128906	127589-127775		
Sporothrix schenckii	Sordariomycetes	AWEQ01000105	c200680-199272	c200637-200454		
Stachybotrys chartarum	Sordariomycetes	APIU01001930	c68323-67004	c68268-68121		
Stachybotrys chlorohalonata	Sordariomycetes	APWP01004580	35482-36792	35537-35681		
Stachybotrys echinata	Sordariomycetes	BCHF01000007	c1950539-1949226	c1950484-1950347		
Stagonospora sp. SRC1lsM3a	Dothideomycetes	LXTA01000084	c430781-429348	c430603-430490	c430417-430312	
Stagonosporopsis tanaceti	Dothideomycetes	JUDZ01007937	c4459-3021	c4314-4190	c4117-4007	
Stemphylium lycopersici	Dothideomycetes	LGLR01000200	c300697-299201	c300492-300376	c300303-300199	
Stenocarpella maydis	Sordariomycetes	NQMI01000186	41422-42717			
Symbiotaphrina buchneri	Xylonomycetes	BCIG01000004	c461674-460088	c461544-461422	c461322-461205	
Talaromyces adpressus	Eurotiomycetes	NHZS01000206	c118972-117519	c118926-118674		
Talaromyces amestolkiae	Eurotiomycetes	MIKG01000003	c1247661-1246234	c1247615-1247378		
Talaromyces atroroseus	Eurotiomycetes	LFMY01000004	832774-834236	832820-833072		
Talaromyces borbonicus	Eurotiomycetes	NBSA01000024	c15220-13785	c15174-14952		
Talaromyces cellulolyticus	Eurotiomycetes	BBPS01001423	949-2402	995-1247		
Talaromyces islandicus	Eurotiomycetes	CVMT01000008	c1318967-1317492	c1318921-1318649		
Talaromyces leycettanus	Eurotiomycetes	JSYV01000806	c7371-5898	c7316-7182		
Talaromyces marneffei	Eurotiomycetes	ABAR01000010	376704-378147	376750-377009		
Talaromyces piceae	Eurotiomycetes	JHNX01000003	436842-438304	436888-437157		
Talaromyces pinophilus	Eurotiomycetes	QNQR01000080	103122-104575	103168-103420		
Talaromyces purpureogenus	Eurotiomycetes	LTAB01000188	c169470-167972	c169430-169272		
Talaromyces stipitatus	Eurotiomycetes	ABAS01000003	232924-234394	232970-233212		
Talaromyces verruculosus	Eurotiomycetes	LHCL01000009	552061-553514	552107-552360		
Talaromyces wortmannii	Eurotiomycetes	MJVA01000858	c559178-557720	c559132-558869		
Taphrina deformans	Taphrinomycotina	CAHR02000056	c33048-31701			
Taphrina flavorubra	Taphrinomycotina	BAVW01000029	c30762-29406			
Taphrina populina	Taphrinomycotina	BAVX01000074	c33446-32080			
Taphrina wiesneri	Taphrinomycotina	BAVU01000030	c88410-87060			
Terfezia boudieri	Pezizomycetes	PZQW01000903	127611-128732			
Thermoascus crustaceus	Eurotiomycetes	BCIC01000004	3086033-3087446	3086088-3086219		
Thermomyces lanuginosus	Eurotiomycetes	ANHP01000262	c53439-52044	c53396-53215		
Thielavia terrestris	Sordariomycetes	CP003013	c3677030-3675620	c3676975-3676798		
Thielaviopsis basicola	Sordariomycetes	PJAC01000009	1842442-1843831	1842461-1842871		
Thielaviopsis ethacetica	Sordariomycetes	BCFY01000041	c158112-156352	c158093-157573		
Thielaviopsis euricoi	Sordariomycetes	BCHJ01000005	c1184154-1182378	c1184135-1183619		
Thielaviopsis musarum	Sordariomycetes	LKBB01000059	c68738-67051	c68719-68208		
Thielaviopsis paradoxa	Sordariomycetes	BCFY01000041	c158112-156352	c158093-157573		
Thielaviopsis punctulata	Sordariomycetes	LAEV01001330	63188-64943	63207-63762		
Tolypocladium capitatum	Sordariomycetes	NRSZ01000683	8412-9657	8479-8607		
Tolypocladium inflatum	Sordariomycetes	AOHE01000120	c350484-349138	c350417-350279		
Tolypocladium ophioglossoides	Sordariomycetes	LFRF01000004	1024441-1025744	1024508-1024640		
Tolypocladium paradoxum	Sordariomycetes	PKSG01000035	7386-8689	7453-7577		
Tolypocladium sp. Salcha MEA-2	Sordariomycetes	JPIJ02000018	c61727-60381	c61660-61522		
Tolypocladium sp. Sup5 PDA-1	Sordariomycetes	JPHH03000003	876978-878318	877045-877179		
Torrubiella hemipterigena	Sordariomycetes	CDHN01000002	661761-663292	661828-662026		
Trichoderma arundinaceum	Sordariomycetes	PXOA01000036	c33226-32032	c33192-32985		
Trichoderma asperellum	Sordariomycetes	JNNP01001120	c20737-19443	c20700-20449		
Trichoderma atroviride	Sordariomycetes	ABDG02000026	c2321862-2320532	c2321825-2321561		
Trichoderma brevicompactum	Sordariomycetes	PXNZ01000104	31180-32384	31214-31421		
Trichoderma citrinoviride	Sordariomycetes	MBDI01000008	88939-90192	88973-89186		
Trichoderma gamsii	Sordariomycetes	JPDN02000004	1266260-1267552	1266297-1266565		
Trichoderma guizhouense	Sordariomycetes	LVVK01000020	c1817692-1816450	c1817655-1817444		
Trichoderma hamatum	Sordariomycetes	ANCB02000201	10056-11341	10084-10336		
Trichoderma harzianum	Sordariomycetes	JOKZ01000073	c70306-69067	c70269-70058		
Trichoderma koningii	Sordariomycetes	BCGH01000003	c2685298-2684046	c2685264-2685049		
Trichoderma koningiopsis	Sordariomycetes	MRBD01000145	c658400-657115	c658363-658099		
Trichoderma longibrachiatum	Sordariomycetes	ANBJ01000133	307620-308872	307654-307869		
Trichoderma parareesei	Sordariomycetes	LFMI01000594	c9620-8377	c9586-9389		
Trichoderma pleuroti	Sordariomycetes	MDJU01000093	59298-60553	59335-59546		
Trichoderma reesei	Sordariomycetes	AAIL02000008	215361-216617	215395-215611		
Trichoderma virens	Sordariomycetes	ABDF02000006	c1012805-1011563	c1012768-1012558		
Trichophyton benhamiae	Eurotiomycetes	ABSU01000020	c175208-173434	c175028-174882		c175168-174747
Trichophyton equinum	Eurotiomycetes	ABWI01001079	6297-8106	6481-6631		6337-6768
Trichophyton interdigitale H6	Eurotiomycetes	AOKS01000628	10210-12033	10394-10546		10250-10683
Trichophyton mentagrophytes	Eurotiomycetes	BFBS01016091	c29610-27787	c29424-29274		c29570-29137
Trichophyton rubrum	Eurotiomycetes	ACPH01000075	c33629-31863	c33462-33315		33589-33180
Trichophyton tonsurans	Eurotiomycetes	ACPI01000601	3497-5306	3681-3831		3537-3968
Trichophyton verrucosum	Eurotiomycetes	ACYE01000215	69961-71726	70142-70290		70001-70425
Trichothecium ovalisporum	Sordariomycetes	PXOC01000620	13253-14553	13314-13433		
Trichothecium roseum	Sordariomycetes	PXNY01000003	c11511-10193	c11450-11331		
Trichothecium sympodiale	Sordariomycetes	PXOB01000183	56936-58238	56997-57116		
Tuber aestivum	Pezizomycetes	CZPR01000106	c60100-58633	c60045-59896		
Tuber borchii	Pezizomycetes	NESQ01000077	26179-27629	26234-26393		
Tuber calosporum	Pezizomycetes	QFET01001887	7986-9444	8041-8197		
Tuber magnatum	Pezizomycetes	PYWC01000059	462582-464048	462637-462790		
Tuber melanosporum	Pezizomycetes	CABJ01001140	c48971-47496	c48916-48754		
Tuber microsphaerosporum	Pezizomycetes	QOVD01000160	7953-9406	8008-8165		
Tuber umbilicatum	Pezizomycetes	QLOI01000966	885-2348	940-1094		
Umbilicaria muehlenbergii	Lecanoromycetes	JFDN01000002	c194415-192847	c194273-194161	c194088-193982	
Uncinocarpus reesii	Eurotiomycetes	AAIW01000200	c101829-100335	c101701-101572		c101789-101454
Ustilaginoidea virens	Sordariomycetes	JHTR01000008	c448308-446892	c448256-448106		
Valsa mali	Sordariomycetes	JUIY01000998	c99786-98519			
Valsa malicola	Sordariomycetes	LKEA01000014	c226713-225390			
Valsa sordida	Sordariomycetes	LJZO01000007	150431-151746			
Venturia asperata	Dothideomycetes	QWXK01000011	675821-677343	675924-676070	676143-676256	
Venturia aucupariae	Dothideomycetes	QWZN01000002	324412-325931	324527-324663	324736-324842	
Venturia carpophila	Dothideomycetes	MECS01000023	c80532-79013	c80429-80283	c80210-80102	
Venturia effusa	Dothideomycetes	MJAE01000004	202786-204399	202979-203138	203211-203319	
Venturia inaequalis	Dothideomycetes	JRPV01001000	c609629-608114	c609517-609381	c609308-609203	
Venturia pyrina	Dothideomycetes	JEMP01000078	c40534-38836	c40428-40289	c40216-40110	
Verruconis gallopava	Dothideomycetes	JYBX01000018	668216-669931	668394-668670	668743-668870	
Verticillium alfalfae	Sordariomycetes	ABPE01000459	4874-6266	4950-5121		
Verticillium dahliae	Sordariomycetes	ABJE01000130	8273-9665	8349-8519		
Verticillium isaacii	Sordariomycetes	NMXN01000004	1391053-1392476	1391129-1391311		
Verticillium klebahnii	Sordariomycetes	NMXL01000004	c1708079-1706653	c1708003-1707818		
Verticillium longisporum	Sordariomycetes	CVQI01002447	c34986-33597	c34910-34740		
Verticillium nonalfalfae	Sordariomycetes	PDEZ01000017	c418533-417140	c418457-418286		
Verticillium nubilum	Sordariomycetes	NMXI01000003	c130987-129605	c130911-130733		
Verticillium tricorpus	Sordariomycetes	JPET01000004	c1675416-1673985	c1675340-1675150		
Verticillium zaregamsianum	Sordariomycetes	NMXM01000003	2491688-2493102	2491764-2491949		
Xeromyces bisporus	Eurotiomycetes	CCCX01000690	44863-46368	44900-45170		
Xylaria longipes	Sordariomycetes	NQIL01000250	c41473-39906	c41418-41032		
Xylaria polymorpha	Sordariomycetes	NQIM01000018	c135970-134398	c135909-135508		
Xylaria sp. JS573	Sordariomycetes	JWIU01000090	c140024-138663	c139963-139780		
Xylaria sp. MSU_SB201401	Sordariomycetes	NPFG01000043	c22282-20741	c22226-21866		
Xylona heveae	Xylonomycetes	JXCS01000017	c48594-47012	c48458-48347	c48274-48160	
Zasmidium angulare	Dothideomycetes	NHNX01000006	1075850-1077322	1075956-1076293		
Zasmidium citri-griseum	Dothideomycetes	NHNW01000018	730275-731720	730387-730697		
Zymoseptoria ardabiliae	Dothideomycetes	AFIU01000450	c7672-6040	c7509-7109		
Zymoseptoria brevis	Dothideomycetes	LAFY01000271	c118852-117293	c118689-118363		
Zymoseptoria passerinii	Dothideomycetes	AFIY01000418	4745-6364	4896-5298		
Zymoseptoria pseudotritici	Dothideomycetes	AFIO01000497	117336-118962	117499-117893		
Zymoseptoria tritici	Dothideomycetes	ACPE01000001	1590760-1592491	1590923-1591314		

Supplementary Table S2

List of *Neurospora crassa* RNA reads from the Sequence Read Archive that cover the 5’ end of the coding region of the *rtnA* gene from which an 174-nt long, intronic sequence is absent. This latter sequence is the 3’-extended internal intron of the secondary stwintron in *N. crassa*.

gnl|SRA|SRR100067.24224772.1
gnl|SRA|SRR447480.22816244
gnl|SRA|SRR400652.22816244
gnl|SRA|SRR400651.14232190
gnl|SRA|SRR627943.22816244
gnl|SRA|SRR676517.22816244
gnl|SRA|SRR1043593.6864561
gnl|SRA|SRR1043593.4143944
gnl|SRA|SRR4175085.2160537
gnl|SRA|SRR1333836.150471571
gnl|SRA|SRR1562550.10669097
gnl|SRA|SRR2007035.15474111
gnl|SRA|SRR2952639.40540779
gnl|SRA|SRR2952639.26696046
gnl|SRA|SRR2952641.37218215
gnl|SRA|SRR3207377.3255250.1
gnl|SRA|SRR3185913.10534254
gnl|SRA|SRR3207375.11766960.1 & gnl|SRA|SRR3207375.11766960.2
gnl|SRA|SRR3207381.10585195.2
gnl|SRA|SRR3207381.9230968.1 & gnl|SRA|SRR3207381.9230968.2
gnl|SRA|SRR3207386.8254619.1
gnl|SRA|SRR5000482.10002120.2
gnl|SRA|SRR5065718.15311487.1
gnl|SRA|SRR5065717.13448515.1
gnl|SRA|SRR5065756.8412314.1
gnl|SRA|SRR5065752.20395615.1
gnl|SRA|SRR5065748.16195714.1
gnl|SRA|SRR5065746.10360892.1
gnl|SRA|SRR5065744.7353275.1

gnl|SRA|SRR5065734.8355460.1
gnl|SRA|SRR5065756.18406579.1
gnl|SRA|SRR5065736.17896328.1
gnl|SRA|SRR5065728.7036935.1
gnl|SRA|SRR5065745.698348.1
gnl|SRA|SRR5065773.17527927.1
gnl|SRA|SRR5065770.21368466.1
gnl|SRA|SRR5065763.4330997.1
gnl|SRA|SRR5065762.14576880.1
gnl|SRA|SRR5065761.16327525.1
gnl|SRA|SRR5065760.17546118.1
gnl|SRA|SRR5065785.5295083.1
gnl|SRA|SRR5065771.8427136.1
gnl|SRA|SRR5065763.13727681.1
gnl|SRA|SRR5065764.21057547.1
gnl|SRA|SRR5065804.5709903.1
gnl|SRA|SRR5065804.3819406.1
gnl|SRA|SRR5065802.5018119.1
gnl|SRA|SRR5065800.9073677.1
gnl|SRA|SRR5065817.19283095.1
gnl|SRA|SRR5065817.13925599.1
gnl|SRA|SRR5065820.18767923.1
gnl|SRA|SRR5065837.23129569.1
gnl|SRA|SRR5065853.8575525.1
gnl|SRA|SRR5065845.12075012.1
gnl|SRA|SRR5065829.22439897.1
gnl|SRA|SRR5065876.719241.1
gnl|SRA|SRR5065858.6761123.1
gnl|SRA|SRR5065873.5745058.1
gnl|SRA|SRR5065865.15562570.1
gnl|SRA|SRR5065875.18423323.1
gnl|SRA|SRR5065875.6098472.1
gnl|SRA|SRR5065871.11265992.1
gnl|SRA|SRR5065916.10492158.1

gnl|SRA|SRR5065894.1517370.1

gnl|SRA|SRR5065916.1649128.1

gnl|SRA|SRR5065916.2907170.1

gnl|SRA|SRR5065907.8932683.1

gnl|SRA|SRR5065904.8145552.1

gnl|SRA|SRR5065894.692726.1

gnl|SRA|SRR5065926.5506790.1

gnl|SRA|SRR5065941.22459160.1

gnl|SRA|SRR5065941.19487232.1

gnl|SRA|SRR5065927.10298644.1

gnl|SRA|SRR5065922.7923815.1

gnl|SRA|SRR5065959.22419547.1

gnl|SRA|SRR5065955.20775695.1 & gnl|SRA|SRR5065955.20775695.2

gnl|SRA|SRR5065962.7867922.1

gnl|SRA|SRR5065962.19360373.1

gnl|SRA|SRR5065952.15752630.1

gnl|SRA|SRR5065985.4917179.1

gnl|SRA|SRR5065973.10941494.1

gnl|SRA|SRR5065993.17894068.1

gnl|SRA|SRR5065991.5567394.1

gnl|SRA|SRR5066015.26456923.1

gnl|SRA|SRR5066010.29170153.1

gnl|SRA|SRR5066003.17942857.1

gnl|SRA|SRR5066001.8095338.1

gnl|SRA|SRR5066001.13362631.1

gnl|SRA|SRR5066014.4955449.1

gnl|SRA|SRR5066011.15734836.1

Supplementary Table S3
Accession numbers of ESTs and TSAs that demonstrate the existence of the “dual intervening sequence” in the transcript of the reticulon-like gene in species of *Onygenales*.

Species	Accession numbers of EST/TSA
Amauroascus mutatus	Accession GDQZ01000714
Amauroascus niger	Accession GDRA01006022
Byssoonygena ceratinophila (canonical U2)	Accession GDRB01009133
Chrysosporium queenslandicum	Accession GDRC01001023
Coccidioides immitis	Accessions GH391836; GH382243; GH384133; GH376123; GH384898; GH337422
Coccidioides posadasii	Accessions GH414948; GH428981; GH446754; CO007951; GH435960; GH428556; BF253074; GH437168; GH428674; GH448138; BF251975; BF252780; CO001624; CO021040; BF251988; CO003313; GH407932; GH423405; GH448484; CO023342; GH449542
Paracoccidioides brasiliensis	Accession CA583413
Trichophyton rubrum	Accessions DW696795; AJ884154; AJ883503; AJ881778; AJ882612; AJ881400; DW687691

Supplementary Table S4
Strains and growth media used in this study. The genome sequence of the *M. cinnamomea rtnA* gene was verified (GenBank MK421638).

Strain	Reference of genome sequence	Reference of media for maintanance and growth
Aspergillus nidulans R21 (ATCC 48756)	Wortman et al., 2009	Pontecorvo et al., 1953
Aspergillus niger ATCC 1015	Andersen et al., 2011	de Vries et al.,2004
Trichoderma reesei QM 9414 (ATCC 26921)	Vitikainen et al., 2010	Mandels & Andreotti, 1978
Helminthosporium solani B-AC-16A	Mattupalli et al., 2014	Mimee et al., 2011
Botrytis cinerea B05.10	Amselem et al., 2011	Fekete et al., 2012
Neurospora crassa OR74A	Galagan et al., 2003	Vogel, 1956
Malbranchea cinnamomea CBS 343.55	Morgenstern et al., 2012	Morgenstern et al., 2012

References to the Supplementary information

Pontecorvo, G., Roper, J. A., Hemmons, L. M., MacDonald, K. D. & Bufton, A. W. J. The genetics of *Aspergillus nidulans*. *Adv. Genet.* **5**, 141–238 (1953).

Andersen, M. R. *et al.* Comparative genomics of citric-acid-producing *Aspergillus niger* ATCC 1015 versus enzyme-producing CBS 513.88. *Genome Res.* **21**, 885–897 (2011).

de Vries, R. P. *et al.* A new black *Aspergillus* species, *A. vadensis*, is a promising host for homologous and heterologous protein production. *Appl. Environ. Microbiol.* **70**, 3954–3959 (2004).

Vitikainen, M. *et al.* Array comparative genomic hybridization analysis of *Trichoderma reesei* strains with enhanced cellulase production properties. *BMC Genomics* **11**, 441; 10.1186/1471-2164-11-441 (2010).

Mandels, M. M. & Andreotti, R. E. Problems and challenges in the cellulose to cellulase fermentation. *Process Biochem.* **13**, 6–13 (1978).

Mattupalli, C., Glasner, J. D. & Charkowski, A. O. A draft genome sequence reveals the *Helminthosporium solani* arsenal for cell wall degradation. *Am. J. Potato Res.* **91**, 517–524 (2014).

Mimee, B., Avis, T. J., Boivin, S., Jabaji, S. & Tweddell, R. J. Effect of iron and nitrogen on the development of *Helminthosporium solani* and potato silver scurf. *Can. J. Plant Pathol.* **33**, 506–511 (2011).

Amselem, J. *et al.* Genomic analysis of the necrotrophic fungal pathogens *Sclerotinia sclerotiorum* and *Botrytis cinerea*. *PLoS Genet.* **7**, e1002230; 10.1371/journal.pgen.1002230 (2011).

Fekete, É. *et al.* Genetic diversity of a *Botrytis cinerea* cryptic species complex in Hungary. *Microbiol. Res.* **167**, 283–291 (2012).

Galagan, J. E. *et al.* The genome sequence of the filamentous fungus *Neurospora crassa*. *Nature* **422**, 859–868 (2003).

Vogel, H. J. A convenient growth medium for *Neurospora crassa*. *Microb. Genet. Bull.* **13**, 42–47 (1956).

Supplementary Table S5

Oligonucleotide primers for RT-PCR verification of stwintron splicing intermediates

Strain	Locus	Primer	Sequence (5'-)
Aspergillus nidulans R21 (ATCC 48756)	AN5404	Anid_D5.6_P1	CTTGCAGTTTCATCGCTTCT
Aspergillus nidulans R21 (ATCC 48756)	AN5404	Anid_D5.6_P2	CGACTCCTTGATGTCTGCAA
Aspergillus nidulans R21 (ATCC 48756)	AN5404	Anid_D5.6_P3	GTGGTGGTCGAAGGAATCAC
Aspergillus niger ATCC 1015	ASPNIDRAFT_198312	Anig_D5.6_P1	CTTTGCGAGAGCGCCTAAGC
Aspergillus niger ATCC 1015	ASPNIDRAFT_198312	Anig_D5.6_P2	GCAGTGGTAGATGAAGGGCTA
Aspergillus niger ATCC 1015	ASPNIDRAFT_198312	Anig_D5.6_P3	TTGAAGAACCAACGGAGGAG
Trichoderma reesei QM9414 (ATCC 26921)	TRIREDRAFT_44705	Trire_D5.6_P1	CACAGCTCTCGACCTCTCTT
Trichoderma reesei QM9414 (ATCC 26921)	TRIREDRAFT_44705	Trire_D5.6_P2	CGGCTTCTAGAGCTCATCGT
Trichoderma reesei QM9414 (ATCC 26921)	TRIREDRAFT_44705	Trire_D5.6_P3	TCTCCCAGGAGAAGAGATGG
Botrytis cinerea B05.10	BCIN_07g03170	Bcin_D5.6_P1	ACACAACCGCCTTTCCATCA
Botrytis cinerea B05.10	BCIN_07g03170	Bcin_D5.6_P2	GGCAGCTGCAGATGCATCTAAA
Botrytis cinerea B05.10	BCIN_07g03170	Bcin_D5.6_P3	CGTTCACGACACTTTGAGTGA
Helminthosporium solani B-AC-16A	HELSOL_RTNA	Hsol_D5.6_P1	TTGCGGTTGTTAGAGTGTGC
Helminthosporium solani B-AC-16A	HELSOL_RTNA	Hsol_D5.6_P2	CCGTTTCAAACCACTCGACCA
Helminthosporium solani B-AC-16A	HELSOL_RTNA	Hsol_D5.6_P3	CAGGACCTGTGGGGAATTTA

Helminthosporium solani B-AC-16A	HELSOL_RTNA	Hsol_D5.6_P4	GGGCATTTGCAAGATCAGAA
Neurospora crassa strain OR74A	NCU06361	Ncra_D5.6_P1	GACTTGACAGCTTCATCCTC
Neurospora crassa strain OR74A	NCU06361	Ncra_D5.6_P2	GTCTATTGGGATGCAAGTCAG
Neurospora crassa strain OR74A	NCU06361	Ncra_D5.6_P3	TCACGGCATCATATGCAGAAG
Neurospora crassa strain OR74A	NCU06361	Ncra_D5.6_P4	CTAACAGAGAAGATGGCAGCG
Malbranchea cinnamomea CBS 343.55	Malci1p7_008099	Malci_D5.6_P1	GTACGTCCGTCTCCTTTGTTT
Malbranchea cinnamomea CBS 343.55	Malci1p7_008099	Malci_D5.6_P2	GAATGTGTGAGACGACTATC
Malbranchea cinnamomea CBS 343.55	Malci1p7_008099	Malci_D5.6_P3	AGAGACAACAGACGCATCGA

Oligonucleotides for cDNA sequencing

**Strain**	**Locus**	**Primer**	**Sequence (5'-)**
Aspergillus nidulans R21 (ATCC 48756)	AN5404	AN5404_seq_F1	GCTTCCGCACAGACTCAAAC
Aspergillus nidulans R21 (ATCC 48756)	AN5404	AN5404_seq_R1	TCATGTCAAAGAAGCAGATT
Aspergillus niger ATCC 1015	ASPNIDRAFT_198312	Anig_seq_F1	GGTCGTTCCCTTCGTCTTCC
Aspergillus niger ATCC 1015	ASPNIDRAFT_198312	Anig_seq_R1	CCGCAATTCTCACAGTTCCATC
Trichoderma reesei QM9414 (ATCC 26921)	TRIREDRAFT_44705	Trire_seq_F1	CTCCCACAGCCAGAAGAAAG
Trichoderma reesei QM9414 (ATCC 26921)	TRIREDRAFT_44705	Trire_seq_R1	GAAGGAAAGAACGTGTCGATA
Botrytis cinerea B05.10	BCIN_07g03170	Bcin_seq_F1	TCGACTGTTTCCCTTCTTCC
Botrytis cinerea B05.10	BCIN_07g03170	Bcin_seq_R1	CGTGATTCTCGCTGACTCCTC
Helminthosporium solani B-AC-16A	HELSOL_RTNA	Hsol_seq_F1	GAAACCCCTCTGTCCGCATTG

Helminthosporium solani B-AC-16A	HELSOL_RTNA	Hsol_seq_R1	AGCCCAGCCTGCGTTGATACT
Neurospora crassa strain OR74A	NCU06361	Ncra_seq_F1	GACTTGACAGCTTCATCCTC
Neurospora crassa strain OR74A	NCU06361	Ncra_seq_R1	GGTATCGTGATCATGGAATCC
Malbranchea cinnamomea CBS 343.55	Malci1p7_008099	Malci_seq_F1	TCGAGTCTCCCGCTCAGCAGC
Malbranchea cinnamomea CBS 343.55	Malci1p7_008099	Malci_seq_R1	TCATCCGATAGTCACAGGGCA

Supplementary Sequence Data. Nucleotide sequences determined during the course of this work (cDNA/genomic DNA) have been deposited at GenBank under accession numbers MK410458–MK410473 and MK421638–MK421641. For the convenience of the reader, below these deposited sequences are listed (in fasta format), ordered by accession number.

Aspergillus nidulans

```
>MK410458 [ASPNID_RTNA_CDNA] [Aspergillus nidulans] [strain=ATCC 48756]
reticulon-like (rtnA) mRNA, 1122 bp
ATGGCCGACAACAGTGAAGTCAGGTATCCCGACCCAAATGCCAACATCAAGGAGTCGATCACAAACGGCCCGGTT
GCTGAAACCATCCGATCCGAGGCCTCCCGCACTGGACAAGAATTTCGCGACCTGAAGAGCGCCAAAGTGATTCCT
TCGACCACCACGGCCACCGGCCAGCCCCTGACTTACTACCACTCTCTGTTCTACAGCCTCCTCAGCTGGGAACAA
CCCCGTGCCACTGCCGCTTCCTTCGCCAGCGTCGTTGCTTTCATTTTCGCGGCCCGTTACCTCCCTCTCCTTCGC
TGGTTCTTCAAGTTCCTCTATCTCGTCCTCGGGGTTACCGCTGCAGCAGAGATTGGCGGCAGGGTCGTTCTCTCT
CAGGGACTCACCAGCTCATTCCGTCCCCGCAAATACTATACCCTGCCTAGGGAAACGATCGAGGCTGTGCTGGAG
GACTTCCAGCAGCTCGTCGATTTCCTCTTCATTGAGTTTCAGCGCATTCTGTTTGCGGAGAATGCCGTTCATACC
ACAGCTGCCGCTGCTGCCGCGTTCTCTGCCTACTGGTTGATTAAGATTCTTCCCTTCTGGGGCCTGTCTCTTTTG
GCCGTCACTATTGCCTACCTTGGACCCTTCGTTTATCTCAACAACCAAGAAATCATTGATGCTCAGATTGAAAAC
GTCCGCCAGATGGTTGGCTCTCAGGCTAACCAACTGAAGGATCTAGCGGAGGAGCGCACCTACCATGCTACCGGT
GTGGTGAAACAGTATGTTGGAGACTACAGCAACAAAGCCCAGGAATATATCGGCCGTCGTTCCGCCTCTCCCGAG
GTCGCCAAAGGCCCTGCTGCAGGGCCTGTAGTCAAGAGGGAACCCGAGCCCGAGGCTGTCGTCAAGACGTCTGAT
TTCCCTGAAGCACCTAAGGTTGAGCCGGTGGCGCAATCAATCGAGACTCAATCAGAGAAGGAGCCTCTCCTGGCT
ATTTAAATGAGTCTGCACGACTCCTGCGGTCCTAATCTCGCCTGGTTTCACGGATTAGACGGGTTGCGACGCAGG
ACCAAACCCAGCTATCTGGCTTCAACGTCCAGGAAAAATTGAGACCCAGTTTTGTGCACTAAAGCAGTCGTA

>MK410459 [ASPNID_RTNA_SPLINT] [Aspergillus nidulans] [strain=ATCC 48756]
reticulon-like (rtnA) precursor RNA, partial, 238 bp
ATCTTCTCCTTTACCAAGTATCTTTGAACTCTCTGCAGTTTGATCATCCCTTGCCCTCTCCACGGCTCTTATCGG
TCCCAAAAAAAAAGCTTTGTTTAGATTGAAGCTTCCGCACAGACTCAAACATGGCCGACAACAGTGAAGTCAGGT
ATCCCGACCCAAATGCCAGTAAGTAATACCATCATCGGCTCAGATTCCGGCGCCCTTGCGAAAGGTCACACAGAC
TAATCGTTTTCGT
```

Aspergillus niger

```
>MK410460 [ASPNIG_RTNA_CDNA] [Aspergillus niger] [strain=ATCC 1015]
reticulon-like (rtnA) mRNA, 1166 bp
CTTGTATCATTTAATTTCAACCCGCCTTGAGCTTCCCCTGAAAACCCGCTCTCTACTCCCGGCCCTTTCCTCCTT
ATCTTTTCCCTCCCGAAAATCCTCCAGACCTTCCGAAACGCACACACACACGACTCTCCGCCATGGCTGACGTGA
GCTATCCCGACACAAATGGCCACAGCGCTCTCGCAAAGGGCCCCATTGCCGATAACATCAAGTCCGAGGCGTCCC
GCACTGGTCAAGAATTCCGCGAATTGAGGAACACTAGCCCTTCATCTACCACTGCGACCGGCCAACCCTTGACCT
ACTACCACTCGCTGCTGTACAGCCTTTTGAGCTGGGAGCAACCCCGAGCTACGGCCATCTCCTTCGCGAGTGTTG
TCACATTCATCTTCGCCGCCCGTTACCTTCCTCTCCTCCGTTGGTTCTTCAAGTTCATCTATGTGGTTCTGGGGT
TCACCGCTATTGTCGAGGTCGGTGGCAGACTTGCTCTGTCACAGAGCGTCGTGAGCTCATTCCGGCCTCGCAAAT
ACTACACCGTGCCCAAGGAAACCATCGAGGCTGTTTTGGAGGACCTTGAGCAGCTCATCGACTTTGTCCTCATTG
AGTTCCAGCGTGTTCTGTTCGCCGAGAACATTGTCCACACCATTGCATCCTTCTTTGCCGCTTTCACCGCCTACT
GGTTGATCAAGTTCCTTCCATTCTGGGGACTGTCCTTGATTGCGGTCACCATCGCGTACATGGGACCCTTGGTCT
ACATCAACAACCGTGAAGTCATCGATGCCCACATTGAGCACGCGCAGGAAGTTGTCAACAGCCAGGCTCACCAGC
TAAAGGATCTTGCCGAAGAGCGCACTGCCCATGCCACGGGTGTTGTGAGGCAGTATGTTGGTGACTACAGCGCCA
AGGCCCAGGGATACATGCGTCGCTCTGCCACCCCCGAGATGGCTAGGGCACCCGCCCCCGTTATCAAGAAGGAGC
CTGAGGCCGAGCCCGAAATCAAGACGACTGACTTCCCGGAGGCACCTAAGGAGGAACCCGTGGCCGCCCAGGAGC
CCGTGGTGGAGACGATCGAACAGCCTGCTGAGCAGGTGAAGTCGGAGCCTCTTCTGGCAGCTTAGGGGGCTGGGG
CCTTTCATACATGATTGGCTATCGGTCGGGATTACTCGGAT

>MK410461 [ASPNIG_RTNA_SPLINT] [Aspergillus niger] [strain=ATCC 1015]
reticulon-like (rtnA) precursor RNA, partial, 240 bp
```

CTTGTATCATTTAATTTCAACCCGCCTTGAGCTTCCCCTGAAAACCCGCTCTCTACTCCCGGCCCTTTCCTCCTT
ATCTTTTCCCTCCCGAAAATCCTCCAGACCTTCCGAAACGCACACACACACGACTCTCCGCCATGGCTGACGTGA
GCTATCCCGACACAAATGGCCACAGTAAGTTCTGAATCTCCCACGCACATACTCCCAGTCTAGGTTACACGAACT
GACCGTTACTCCCTC

>MK410462 [ASPNIG_RTNA_EXONSKIP] [Aspergillus niger] [strain=ATCC 1015]
reticulon-like (rtnA) alternatively spliced mRNA, 1151 bp
CTTGTATCATTTAATTTCAACCCGCCTTGAGCTTCCCCTGAAAACCCGCTCTCTACTCCCGGCCCTTTCCTCCTT
ATCTTTTCCCTCCCGAAAATCCTCCAGACCTTCCGAAACGCACACACACACGACTCTCCGCCATGGCTGACGTGA
GCTATCCCGACACAAATGGCCACAGCCCCATTGCCGATAACATCAAGTCCGAGGCGTCCCGCACTGGTCAAGAAT
TCCGCGAATTGAGGAACACTAGCCCTTCATCTACCACTGCGACCGGCCAACCCTTGACCTACTACCACTCGCTGC
TGTACAGCCTTTTGAGCTGGGAGCAACCCCGAGCTACGGCCATCTCCTTCGCGAGTGTTGTCACATTCATCTTCG
CCGCCCGTTACCTTCCTCTCCTCCGTTGGTTCTTCAAGTTCATCTATGTGGTTCTGGGGTTCACCGCTATTGTCG
AGGTCGGTGGCAGACTTGCTCTGTCACAGAGCGTCGTGAGCTCATTCCGGCCTCGCAAATACTACACCGTGCCCA
AGGAAACCATCGAGGCTGTTTTGGAGGACCTTGAGCAGCTCATCGACTTTGTCCTCATTGAGTTCCAGCGTGTTC
TGTTCGCCGAGAACATTGTCCACACCATTGCATCCTTCTTTGCCGCTTTCACCGCCTACTGGTTGATCAAGTTCC
TTCCATTCTGGGGACTGTCCTTGATTGCGGTCACCATCGCGTACATGGGACCCTTGGTCTACATCAACAACCGTG
AAGTCATCGATGCCCACATTGAGCACGCGCAGGAAGTTGTCAACAGCCAGGCTCACCAGCTAAAGGATCTTGCCG
AAGAGCGCACTGCCCATGCCACGGGTGTTGTGAGGCAGTATGTTGGTGACTACAGCGCCAAGGCCCAGGGATACA
TGCGTCGCTCTGCCACCCCCGAGATGGCTAGGGCACCCGCCCCCGTTATCAAGAAGGAGCCTGAGGCCGAGCCCG
AAATCAAGACGACTGACTTCCCGGAGGCACCTAAGGAGGAACCCGTGGCCGCCCAGGAGCCCGTGGTGGAGACGA
TCGAACAGCCTGCTGAGCAGGTGAAGTCGGAGCCTCTTCTGGCAGCTTAGGGGGCTGGGGCCTTTCATACATGAT
TGGCTATCGGTCGGGATTACTCGGAT

>MK410463 [ASPNIG_RTNA_ALTSPLINT] [Aspergillus niger] [strain=ATCC 1015]
reticulon-like (rtnA) alternatively spliced precursor RNA, partial, 211 bp
CTTGTATCATTTAATTTCAACCCGCCTTGAGCTTCCCCTGAAAACCCGCTCTCTACTCCCGGCCCTTTCCTCCTT
ATCTTTTCCCTCCCGAAAATCCTCCAGACCTTCCGAAACGCACACACACACGACTCTCCGCCATGGCTGACGTGA
GCTATCCCGACACAAATGGCCACAGTAAGTCTAGGTTACACGAACTGACCGTTACTCCCTC

Helminthosporium solani

>MK410464 [HELSOL_RTNA_CDNA] [Helminthosporium solani] [strain=B-AC-16A]
reticulon-like (rtnA) mRNA, 1151 bp
CTACTACGACTACTACTACTACTACTACTGCTAGTAGCTCTGGAACCATACGTATTGTAGCATGTCCGACCTCAG
CCTCGACAACAATCGCCCCGTCGACATGGACGCCAACGGCAGCTCGACTGTGACTGACCACGCCGTCAACGCAAA
GGACAGCCTGTTCCAATCAGCGTCGTCCGCCATGGCCGCCGTTTCAAACCACTCGACCACCCAGAATCTAAAGTC
CAACATTGCAAATGGTCCTGTTGCAGAAAAGGCCAAAGCTGAGGGCGCAAAGACAAAGGCTGAGTTTTCTGATCT
TGCAAATGCCCGAACAACACCAGATCAGCCCGCCGCGACTGGCCAGCCTTTGACCCACTATCACTCCATGTTCTA
CCGCCTCTTGAGTTGGAAGAACCCCCGTGCGACTGCCATCTCCTTTGCTGCTGCCGTGATCTTCATATTCGCCGC
CCGTTACCTCAACGTCATCCGCTACATCTTCAAGGCTCTTTACATGACTCTGGGCATCACGGCTACCGCCGAACT
TGCCGGATCGATGACCATTGGCAAGGGCTTGACCTCTCAATTCAGGCCGCGACAGTATTTCACAATTCCCAAGGC
TTCGCTTGAACGCCTTTTGGACGATGTGGAGCAGTTTATCAACTTCTTCGTCATCGAGTCGCAGCGGATAGTCTT
TGCCGAGAATGTGTACGCAACGGTTGCTGCATTCTTCGCGTCGCTCATCTCGTACTTCCTTATCAAGTTTGTTCC
CCTCTGGGGCTTGTCTCTGATTGCCACGGTTATTGCCTATCTGGGCCCGCTCGTCTACATCAAGAACAAAGAGGT
TATCGATGCCCAACTTGAGAAGGGCTATCACATTGCCAACCAGCAGGCGACGCAGCTCAAAAACCTCGCCTCTCA
GCACACTGGGAATGCAGCCAAGACTGTTCAGGGTTATACTCAACAGTACACCGCGAAGGCGCAGGAGACGATCAA
CTCCTACCGTGGACGCTCTGCTTCCCCCGAAGCCAAGAAGCAAGACCTCCCTTCTGCACCAACACATGAGCCTGC
TCCTGTTATCAAGGAGGAGCCTGTAGCCGAGCCTGCCTTGTAAGCGCTCGAAACAGTCGCTGCCCAGTCTGTAAG
CCGGCAGTGAATCAAAGGCCTTGAAA

>MK410465 [HELSOL_RTNA_SPLINT1] [Helminthosporium solani] [strain=B-AC-16A]
reticulon-like (rtnA) precursor RNA, partial, 205 bp
CTACTACGACTACTACTACTACTACTACTGCTAGTAGCTCTGGAACCATACGTATTGTAGCATGTCCGACCTCAG
CCTCGACAACAATCGCCCCGTCGACATGGACGCCAACGGCAGCTCGACTGTGACTGACCACGCCGTCAACGCAAA
GGACAGCCTGTTCCAATGTGAGTCCTTATCTGCACCTCGCACACCACATCCCCTC

>MK410466 [HELSOL_RTNA_SPLINT2] [Helminthosporium solani] [strain=B-AC-16A]
reticulon-like (rtnA) precursor RNA, partial, 210 bp

CCCAGAATCTAAAGTCCAACATTGCAAATGGTGAGTGCCCAAAAGCCCAAAATTCCCTCCAGCGTCCCCCCACCC
CCGCAGGCTCTTCACGTCCTCCGAGTGCCGTACCCACCCGTGCAAATTCTCTTCATCTGAATAAAAGTGGCCAAC
ACTCAGTAATGTTGACTGGGCGGGATTTGGCGGAAGAAATGAGAATACGGGATAGCCTGC

Trichoderma reesei

>MK410467 [TRIREE_RTNA_CDNA] [Trichoderma reesei] [strain=QM9414]
reticulon-like (rtnA) mRNA, 935 bp
CCCTCTTCTACCACCGAATTTTCTCGACGACAGAACCAATTCAAAACCAGAGCATCATGGCTGATAGCGGCGCGG
TCAACGGCCACTCCAAAGCCGCCAACGGCAGCACTGGAGAGTCTCTGGCACCCTACCGTTCGCTCTTTTCCCATC
TCTTCTCCTGGGAGAATCCTCGTGATTCGGCCGTCGCCTATGTCTCCGTCGTTGCTAGCATCGCCGCCGTCCGGT
ATCTTAGCCTCGTTCGCTGGGCCCTCGGATTGTCCTGGATGGTCCTGGCCACCACCGTCGCTGCCGAGGTCGCGG
GCAAAGCAGTTCTCAACAACGGCCTGGCTTCCCAGCTTCGCCCTCGCGAGTACTATACCCTGCCTCGCGAAGCAT
TCGACAGGCTAGTTAGCGAGGCTCACGGACTCATCAACTTCTTCTTGGTTGAAGCCCAGCGCATTGTCTTTGTCG
AGAATGTCTACGCCTCGTCTGCTGCCTTCGCCGTTACCCTTCTTTCCAGCCTTCTCATCAAGGTTCTGCCCTACT
GGGTCCTTGCCATCGTTGCAACCACCATTGCCTTCTTCGCGCCTTTGATCTATTCCAGAAACCAGGAGTTCATCG
ACCAGCAGCTCAAGTCTGCTGCCGACGTCATCGGCGCCCAGACTGCCCAAGTCCGAGATGCCGCTCAGAAGCAGG
CCGATCACCTCGCTTCCGTGTCGAAGCAGTATGCTGGAGACTACACTGAGAAGGTTCACGAGATTCTGCGCACCC
GCAGCCTGTCTCCCTCGGCCGCCAAGAAGTCGCCTGTCGATTTCCCCTCCCCTCCTACTGAGGAGCCCAAGGCCG
CCGAACAACCCACCCCTCAGGAGCCTATCGTTGCTTAGATGAGCTCAATTGACCCGCACGTGTGATTATGGTCAA
GAAACGATAAACGCCAAGTTGAGCTCCACAGCAAC

>MK410468 [TRIREE_RTNA_SPLINT] [Trichoderma reesei] [strain=QM9414]
reticulon-like (rtnA) precursor RNA, partial, 216 bp
TTCCGCCGCCCGTATCACAACTTGCGCGACCGACAAAGCCCCTCCCACAGCCAGAAGAAAGCCCTCTTCTACCAC
CGAATTTTCTCGACGACAGAACCAATTCAAAACCAGAGCATCATGGCTGATAGCGGCGCGGTCAACGGCCACTCC
AGTAAGTTTTTACATGTGACAGACAAGGCCTCAGAGCCCGCCACCTTGCCCCTTCTGTATAGCTG

Botrytis cinerea

>MK410469 [BOTCIN_RTNA_CDNA] [Botrytis cinerea] [strain=B05.10] reticulon-like (rtnA) mRNA, 1125 bp
CAAGCTTATCATCACAAGCTTCCTCGACTGTTTCCCTTCTTCCACACACAACCATCAATTTCCATACATACTATG
TCAGATTTTCCTTCCAACGCCAGCACAACTTCCACAGAAGGGACTGCCAGCACAATGGAGAACGCAAAGAATGCA
GCCTATGACAATGCATCTGCAGCTGCCAACGCTGTTGGAAATCACCCTATCACTCAAAGTGTCGTGAACGGCCCA
ATGGCAGATAATGTACGAGACCAACATCAAAAGACTCAGGTCGAATTTTCCAACCTCGCAAACTCTCGCACCACT
CCTACCACTCAAACAGCTACCGGCCAACAACTCACACATTACCATTCCTTCTTCTACAATTTGTTGTCATGGGAG
AATCCTCGTGCGTCTGGAATTGCATACCTTTCTACCGTTGCATTCATCTTCGCTGCCAGATATCTTGACATTCTC
CGATATTCATTCAAGATTACATACATGACTTTGTTTGTGACGGTTCTTGCAGAAGGTCTCGGAAAGCTAGCTTTC
TCGCATGGACTCACTTCTCAATTTAGACCTAAGAAGTATTACACGGTTTCTAAAGAAACTCTTGATAGCTTACTC
GGTGACGTCCATGAGCTTATCAACTTCTTCGTCATTGAGTCACAACGTGTTCTCTTTGCCGAGAACGTATTTGTT
TCTCTCACTGCCTTGATTGGATCTTTCATTTCCTACTACTTGATCAAACTTATCCCATTCTGGGGTCTCTCTCTC
ATCGCTACTTCCGTTCTCTTCATCTCCCCACTTGTTTACAAGACCAACAAGGAGCTTATTGACAACACTCTTGCT
CAAATCTCAAGCATTGTCAACCAACAAACCGAACAAGTCAAGCAACTTGCCAGCCAACACGCTTCGCATGTTGCC
GAGACCACGAAGCAATACGCTGGAGAGTATGCTCACAAGGCACAAGAAATGATCGGAAGTGCTCGAAGCCGCTCT
GGATCTCCAGAGGCTAGCAGAGTTGCCTCCCCAACCGCCAAGGCCAACGGTATCAAGGAGGAGGATTTCCCAACA
GCACCAAAGGAAGAGTTCAAATCCGGAATTGCTGCTGAGGAGGAGCCACTCATTGCTGCATAAATCTAGCAGAAA

>MK410470 [BOTCIN_RTNA_SPLINT] [Botrytis cinerea] [strain=B05.10]
reticulon-like (rtnA) precursor RNA, partial, 216 bp
CAAGCTTATCATCACAAGCTTCCTCGACTGTTTCCCTTCTTCCACACACAACCATCAATTTCCATACATACTATG
TCAGATTTTCCTTCCAACGCCAGCACAACTTCCACAGAAGGGACTGCCAGCACAATGGAGAACGCAAAGAATGCA
GCCTATGACAGTAAGTTCACAACTTCGCACAATAATATCAATACAGAAAATCAACTAATCAATCAA

Neurospora crassa

>MK410471 [NEUCRA_RTNA_CDNA] [Neurospora crassa] [strain=OR74A] reticulon-like (rtnA) mRNA, 1180 bp
CATCCGCGAATCTTTCCTCTGGTCAACAACCACCAAGAAACAAGCAACAAACTTTGTACCACACGTTATCAATAG
CAGGGACACCCAAATACCTTTACAATGGCCGACGACTACTCCAACGGCTCGACTAACCTCGATGCCCTCAAAAAC
AACGCTGCTTCTGCATATGATGCCGTGACGAAGGGCCCCGTCGCTCAGAACGTTGCCGACCAGCACGCCAAGACT

GTTGACGAGCTCTCCAACCTTTCTGCGTCTCGTCGCACTCCCTCGACCCCCGCGGCTACTGGCCAACCCTTGACA
CACTACCACTCGTTCTTTTCCGAGCTGTTGTCATGGAACAACCCTCGTGCCTCTGCCATTGCTTACCTGAGCATC
GTCGCTGCCATCTTCTCTGTTAGGTACCTTGATATGCTCCGCTTGGGTTTCAAGCTCACTTGGATGGTCCTCGGT
GTTACCGTTACCGCCGAAGTTGCCGGCAAGACCGTTCTCAACCAGGGCTTGGCTACCCAGCTGCGCCCGCGCAAG
TATTACACTGTTTCCAAGGAGACTCTGGATAGCGCTATTGGCGATGTTCATGAGCTCGTCAACTTCTTCGTCATT
GAGGCTCAGCGTATTCTCTTCGCTGAAAATGTCTACGTGTCCGGCCTCGTTGCCATCGCCGCTTTCTTCGCCTAT
TATCTCGTCAAGATCGTTCCCTACTGGGGTCTTGCCCTGATTGCCACCACTCTTGCCTTCCTCGGGCCCCTCGTC
TACAAGACCAACAAGGAGTTTATCGATGCCCAGCTTCACCGTGCTAGCGAGATCATCGGCGCCCAGACTGCTCAG
CTCAAGACCGCGGCTCAGCAGAACAGCGAGAAGGCTACCCAGTTGACCAAGCAGTTTGTTGGCGAGTATACCGCC
AAGGCCCAGACGCTGGTCAGCAAGGCTACTCCTGCCTCTCTCCACGGTGAGGAGCACACCAACGGTCATGCCAAC
AAGTCCGAGCTCCGCGACGCCGACTTTCCTCCCGCCCCCCAGGCCGACATCCAGACCGAGCCTGTCGTTCCCGTC
ACTGTCAAGAGCGGTGAGCAGCAGCCTCTGCTGATTTAAGCGAGCTTCATTGGTTAAACCTTCTACGGCAAAAAT
CATGCTGTGGTCGGTTTCACACTTTACCTGAATACACCATGGCCGGCAAAGTCCG

>MK410472 [NEUCRA_RTNA_SPLINT] [Neurospora crassa] [strain=OR74A] reticulon-like (rtnA) precursor RNA, partial, 227 bp
CATCCGCGAATCTTTCCTCTGGTCAACAACCACCAAGAAACAAGCAACAAACTTTGTACCACACGTTATCAATAG
CAGGGACACCCAAATACCTTTACAATGGCCGACGACTACTCCAACGGCTCGACTAACCTCGATGCCCTCAAAAAC
AGTAAGTGTTCCACAACAGCTTCCAATGCCACCAACGCCAGCGACTCCTTTCGCCATGACATCTCACAATGCAGA
AA

Malbranchea cinnamomea

>MK410473 [MALCIN_RTNA_CDNA] [Malbranchea cinnamomea] [strain=CBS 343.55] reticulon-like (rtnA) mRNA, 983 bp
AAGCGCCCCTCCGACGCCATGGCCAGACCTGCGTCCTCTCCCCAGCCAGGGGCTGCTCAGAAACCCTTCGTCAAT
GGCTTCACCGTCACTGGCCAGCCTTTGACCCCATACCATTCCCGCCTGTATAGCCTGCTGAGCTGGCAACGTCCC
CGTCACACGGCCATCTCCTATGTCACCGTCGTCTTCACCATCCTCGCCGCCCGCTTCCTTCCGCTGCTGCGCTGG
GCGCTCAAGTTTGTCTATCTGTCTCTTGGAATTGCCGCCAGCATGGAAGTCCTCGGAAGGGCGGTCTTCTCCCGT
GGAATCACCACCGATGCCCGTCCACGCAAGTACTACACCATCCCCAAGGACACCGTCGAGAGCGTCTTGGAGGAC
ATGGAGCAGCTGGTCGACTTTTTCCTCATCGAGTTCCAGCGCATTCTGTTTGTCGAGAATCTGACTTACACCATT
GGGGCATTCTTCGCGGCCTTGGTGACCTACTCACTCGTCAGGTTTTTGCCTCTCTGGGGTCTTACCCTGCTGGGC
GTCACCGTCGCGTACATGGGCCCCCTGATCTACATCAACAACAAGGAGTTCATCGATGAGCAGATCAATGCTCTG
CAGGACATCATCAACGCCCAGACCGTCCATGTCAAGGAGATGGCCGAAACCCGCACCGCCCACGCCACCGAGGTC
GTGAGGCACTATGTCAACGACTACCGTGCCCGGGCTCACGACTACGTCGTTTCGACTCGCGCTCGCCATGCCACC
CCCGAGGCGGAGAGGCGCCTGGCCAAGCCCGAAGAACTCGCGGGTCTCCAGCCTGAACGCGGCCGCGAGCCCGGC
ACCGGACTCCAGCATGCGGACTTTCCCGCGGCCCCGCAGACCGACATTGTCCCCGAGGTGAGAGCGGCCACTCCG
ATCACGGAAGTGAAGAGGGAGGAGACGCGCACTCCCCCAATTGCCGCCCTTTGATGTTTCCCTTTTTTTCACGCT
CGGGGGCC

>MK421638 [MALCIN_RTNA_GENOMIC] [Malbranchea cinnamomea] [strain=CBS 343.55] reticulon-like (rtnA) gene, 1757 bp
AAGCGCCCCTCCGACGCCATGGCCAGACCTGCGTCCTCTCCCCAGCCAGGTACGTCCGTCTCCTTTGTTTCTTTC
TTTCTCCTTGATTTCTATTATATATACCTCCTTGAGAGAGACAGAGTGTGTGGGTGAATTGGTTGATGATCAGTA
AGGTGAGTTGCCATTTTCTTTTTCTGTTCTACTCCTTTTCCGAAAAAAAAAAAAAAAAACAAAACATTGACTGAC
CACCGTCCTTTCTCTCGTTCTTTTGCTACGTCCAGTGCTCTCCCTCCTCCTCCTCCTCCTCCTCGAAAACGACCA
CGGGGGATATCGATAGTCGTCTCACACATTCGACAGGCACGCAGTCGGTTGCCTCGACACTGATCATGGCCAGTA
CTGACAATGGTACGTTGCGGAGACGGATCTGGGGGTGTGTGGGAGCTGGCGGATGTCTGGTTTCGCCACCCTGAC
CTCATAGTGGTTTTACTTGACTGACTCGATGCGTCTGTTGTCTCTTAGATCTCGGGCTCGCAGGGGCTGCTCAGA
AACCCTTCGTCAATGGCTTCACCGTCACTGGCCAGCCTTTGACCCGTAGGTTTCCCCCCTGCCTCGTCCCTACGT
CCATCCTCCTTCCTCTGGACAACTCGCGACTGACCTCCTTTCCTTCTTCTTCTTTTCTTCGGTTGGCCCGTCTGT
AGCATACCATTCCCGCCTGTATAGCCTGCTGAGCGTGAGTGCACTCCCCGTCCCCCTCCCGCCCCCCCCAAATAA
CTCGAGGGGCTCGATGCTGAGAGCCGTGTGTTTCGCCAGTGGCAACGTCCCCGTCACACGGCCATCTCCTATGTC
ACCGTCGTCTTCACCATCCTCGCCGCCCGCTTCCTTCCGCTGCTGCGCTGGGCGCTCAAGTTTGTCTATCTGTCT
CTTGGAAGTATGTCCAAGCTTCCATTCCATGTCTACCTGACCGGCGGACGGGGCGACGTGTCTTAACTAATCCTT
GCGTAGTTGCCGCCAGCATGGAAGTCCTCGGAAGGGCGGTCTTCTCCCGTGGAATCACCACCGATGCCCGTCCAC
GCAAGTACTACACCATCCCCAAGGACACCGTCGAGAGCGTCTTGGAGGACATGGAGCAGCTGGTCGACTTTTTCC
TCATCGAGTTCCAGCGCATTCTGTTTGTCGAGAATCTGACTTACACCATTGGGGTAAGTAACGTCATTTCTGAAT
CGACGGACGGGATTGACGCAGGACTAGGCATTCTTCGCGGCCTTGGTGACCTACTCACTCGTCAGGTTTTTGCCT
CTCTGGGGTCTTACCCTGCTGGGCGTCACCGTCGCGTACATGGGCCCCCTGATCTACATCAACAACAAGGAGTTC
ATCGATGAGCAGATCAATGCTCTGCAGGACATCATCAACGCCCAGACCGTCCATGTCAAGGAGATGGCCGAAACC

CGCACCGCCCACGCCACCGAGGTCGTGAGGCACTATGTCAACGACTACCGTGCCCGGGCTCACGACTACGTCGTT
TCGACTCGCGCTCGCCATGCCACCCCCGAGGCGGAGAGGCGCCTGGCCAAGCCCGAAGAACTCGCGGGTCTCCAG
CCTGAACGCGGCCGCGAGCCCGGCACCGGACTCCAGCATGCGGACTTTCCCGCGGCCCCGCAGACCGACATTGTC
CCCGAGGTGAGAGCGGCCACTCCGATCACGGAAGTGAAGAGGGAGGAGACGCGCACTCCCCCAATTGCCGCCCTT
TGATGTTTCCCTTTTTTTCACGCTCGGGGGCC

>MK421639 [MALCIN_RTNA_SPLINT1] [Malbranchea cinnamomea] [strain=CBS 343.55] reticulon-like (rtnA) splicing intermediate-1 precursor RNA, partial, 217 bp
AAGCGCCCCTCCGACGCCATGGCCAGACCTGCGTCCTCTCCCCAGCCAGGTACGTCCGTCTCCTTTGTTTCTTTC
TTTCTCCTTGATTTCTATTATATATACCTCCTTGAGAGAGACAGAGTGTGTGGGTGAATTGGTTGATGATCAGTA
AGTGCTCTCCCTCCTCCTCCTCCTCCTCCTCGAAAACGACCACGGGGGATATCGATAGTCGTCTCAC

>MK421640 [MALCIN_RTNA_SPLINT2] [Malbranchea cinnamomea] [strain=CBS 343.55] reticulon-like (rtnA) splicing intermediate-2 precursor RNA, partial, 286 bp
AAGCGCCCCTCCGACGCCATGGCCAGACCTGCGTCCTCTCCCCAGCCAGGTACGTCCGTCTCCTTTGTTTCTTTC
TTTCTCCTTGATTTCTATTATATATACCTCCTTGAGAGAGACAGAGTGTGTGGGTGAATTGGTTGATGATCAGCA
CGCAGTCGGTTGCCTCGACACTGATCATGGCCAGTACTGACAATGGTACGTTGCGGAGACGGATCTGGGGGTGTG
TGGGAGCTGGCGGATGTCTGGTTTCGCCACCCTGACCTCATAGTGGTTTTACTTGACTGAC

>MK421641 [MALCIN_RTNA_SPLINT3] [Malbranchea cinnamomea] [strain=CBS 343.55] reticulon-like (rtnA) splicing intermediate-3 precursor RNA, partial, 1079 bp
TGTTTCTTTCTTTCTCCTTGATTTCTATTATATATACCTCCTTGAGAGAGACAGAGTGTGTGGGTGAATTGGTTG
ATGATCAGCACGCAGTCGGTTGCCTCGACACTGATCATGGCCAGTACTGACAATGATCTCGGGCTCGCAGGGGCT
GCTCAGAAACCCTTCGTCAATGGCTTCACCGTCACTGGCCAGCCTTTGACCCCATACCATTCCCGCCTGTATAGC
CTGCTGAGCTGGCAACGTCCCCGTCACACGGCCATCTCCTATGTCACCGTCGTCTTCACCATCCTCGCCGCCCGC
TTCCTTCCGCTGCTGCGCTGGGCGCTCAAGTTTGTCTATCTGTCTCTTGGAATTGCCGCCAGCATGGAAGTCCTC
GGAAGGGCGGTCTTCTCCCGTGGAATCACCACCGATGCCCGTCCACGCAAGTACTACACCATCCCCAAGGACACC
GTCGAGAGCGTCTTGGAGGACATGGAGCAGCTGGTCGACTTTTTCCTCATCGAGTTCCAGCGCATTCTGTTTGTC
GAGAATCTGACTTACACCATTGGGGCATTCTTCGCGGCCTTGGTGACCTACTCACTCGTCAGGTTTTTGCCTCTC
TGGGGTCTTACCCTGCTGGGCGTCACCGTCGCGTACATGGGCCCCCTGATCTACATCAACAACAAGGAGTTCATC
GATGAGCAGATCAATGCTCTGCAGGACATCATCAACGCCCAGACCGTCCATGTCAAGGAGATGGCCGAAACCCGC
ACCGCCCACGCCACCGAGGTCGTGAGGCACTATGTCAACGACTACCGTGCCCGGGCTCACGACTACGTCGTTTCG
ACTCGCGCTCGCCATGCCACCCCCGAGGCGGAGAGGCGCCTGGCCAAGCCCGAAGAACTCGCGGGTCTCCAGCCT
GAACGCGGCCGCGAGCCCGGCACCGGACTCCAGCATGCGGACTTTCCCGCGGCCCCGCAGACCGACATTGTCCCC
GAGGTGAGAGCGGCCACTCCGATCACGGAAGTGAAGAGGGAGGAGACGCGCACTCCCCCAATTGCCGCCCTTTGA
TGTTTCCCTTTTTTTCACGCTCGGGGGCC
